# Accuracy of the Wound Healing Questionnaire in the diagnosis of surgical-site infection after abdominal surgery in low- and middle-income countries

**DOI:** 10.1093/bjs/znad446

**Published:** 2024-02-26

**Authors:** NIHR Global Health Research Unit on Global Surgery, James Glasbey, Adesoji Ademuyiwa, Alisha Bhatt, Bruce Biccard, Jane Blazeby, Peter Brocklehurst, Sohini Chakrabortee, J C Allen Ingabire, Francis Moïse Dossou, Irani Durán, Rohini Dutta, Dhruva Ghosh, Frank Gyamfi, Parvez Haque, Pollyanna Hardy, Mike Horton, Gabriella Hyman, Ritu Jain, Oluwaseun Ladipo-Ajayi, Ismail Lawani, Souliath Lawani, Mwayi Kachapila, Rachel Lillywhite, Rhiannon Macefield, Laura Magill, Janet Martin, Jonathan Mathers, Kenneth McLean, Punam Mistry, Rohin Mittal, Mark Monahan, Rachel Moore, Dion Morton, Moyo Ojo, Faustin Ntirenganya, Emmanuel Ofori, Rupert Pearse, Alberto Peón, Thomas Pinkney, Antonio Ramos de la Medina, Tubasiime Ronald, David Roman, Emmy Runingamugabo, Alice Sitch, Anita Slade, Stephen Tabiri, Donna Smith, Aneel Bhangu, James Glasbey, Alice Sitch, Anita Slade, Duc Khanh To, Aneel Bhangu, Pollyanna Hardy, Adesoji O Ademuyiwa, Lawani Ismail, Dhruva Ghosh, Antonio Ramos de la Medina, Rachel Moore, Faustin Ntirenganya, Stephen Tabiri, Emmy Runingamugabo, Simin Patrawala, Angela Prah, Christian Oko, Karolin Kroese, Ismaïl Lawani, Francis Moïse Dossou, Corinne Dzemta, Covalic Melic Bokossa Kandokponou, Souliath Lawani, Hulrich Behanzin, Cyrile Kpangon, Bernard Appiah Ofori, Stephen Tabiri, Abdul-Hafiz Saba, Gbana Limann, Daniel Kwesi Acquah, Shamudeen Mohammed Alhassan, Sheriff Mohammed, Owusu Abem Emmanuel, Yakubu Musah, Yenli Edwin, Sheba Kunfah, Yakubu Mustapha, Abantanga Atindaana Francis, Emmanuel Ayingayure, Gbana Limann, Forster Amponsah-Manu, Eric Agyemang, Vera Agyekum, Esther Adjei-Acquah, Emmanuel Yaw Twerefour, Barbra Koomson, Ruby Acheampong Boateng, Ato Oppong Acquah, Richard Ofosu-Akromah, Leslie Issa Adam-Zakariah, Nii Armah Adu-Aryee, Theodore Wordui, Coomson Christian Larbi, Akosa Appiah Enoch, Mensah Elijah, Kyeremeh Christian, Addo Gyambibi Kwame, Boakye Percy, Kontor Effah Bismark, Gyamfi Brian, Manu Ruth, Romeo Hussey, Samuel Dadzie, Akosua Dwamena Appiah, Grace Yeboah, Cynthia Yeboah, James Amoako, Regina Acquah, Naa Anyekaa Sowah, Atta Kusiwaa, Esther Asabre, Cletus Ballu, Charles Gyamfi Barimah, Frank Owusu, Clement Sie-Broni, Vivian Adobea, Prince Yeboah Owusu, Marshall Zume, Abdul-Hamid Labaran, Raphael Adu-Brobbey, Martin Tangnaa Morna, Samuel A Debrah, Patrick Opoku Manu Maison, Michael Nortey, Donald Enti, Mabel Pokuah Amoako-Boateng, Anthony Baffour Appiah, Emmanuel Owusu Ofori, Richard Kpankpari, Benedict Boakye, Elizabert Mercy Quartson, Patience Koggoh, Anita Eseenam Agbeko, Frank Enoch Gyamfi, Joshua Arthur, Joseph Yorke, Christian Kofi Gyasi-Sarpong, Charles Dally, Agbenya Kobla Lovi, Michael Amoah, Boateng Nimako, Robert Sagoe, Anthony Davor, Fareeda Galley, Michael Adinku, Jonathan Boakye-Yiadom, Jane Acquaye, Juliana Appiah, Dorcas Otuo Acheampong, Iddrisu Haruna, Edward Amoah Boateng, Emmanuel Kafui Ayodeji, Samuel Tuffuor, Naa Kwarley, Yaa Tufuor, Ramatu Darling Abdulai, Fred Dankwah, Ralph Armah, Doris Ofosuhene, Dorcas Osei-Poku, Arkorful Ebenezer Temitope, Delali Akosua Gakpetor, Victoria Sena Gawu, Christopher Asare, Enoch Tackie, James Ankomah, Isaac Omane Nyarko, Zelda Robertson, Serbeh Godwin, Appiah Anthony Boakye, Godfred Fosu, Frank Assah-Adjei, Parvez Haque, Ritu Jain, Alisha Bhatt, Jyoti Dhiman, Rohini Dutta, Dhruva Ghosh, Esther Daniel, Priyadarshini K, Latha Madankumar, Rohin Mittal, Ida Nagomy, Soosan Prasad, Arpit Jacob Mathew, Danita Prakash, Priya Jacob, Jeremiah P Anachy, Amy Mathew, Josy Thomas, Philip V Alexander, Pradeep Zechariah, Neerav D Aruldas, Asif Mehraj, Hafsa Imtiyaz Ahmed, Rauf A Wani, Fazl Q Parray, Nisar A Chowdri, Antonio Ramos De la Medina, Laura Martinez Perez Maldonado, Diana S Gonzalez Vazquez, Iran I Durán Sánchez, Maria J Martínez Lara, Alejandra Nayen Sainz de la Fuente, Ana O Cortes Flores, Mariana E Barreto Gallo, Alejandro Gonzalez Ojeda, Monica E Jimenez Velasco, Luis Hernández Miguelena, Reyes J Cervantes Ortiz, Gonzalo I Hernandez Gonzalez, Marco Hurtado Romero, Rosa I Hernandez Krauss, Luis A Dominguez Sansores, Alejandro Cuevas Avendaño, Celina Cuellar Aguirre, Isaac Baltazar Gomez, Hector Ortiz Mejia, Alejandro González Ojeda, Oscar E Olvera Flores, Erick A González García de Rojas, Kevin J Pintor Belmontes, Francisco J Barbosa Camacho, Aldo Bernal Hernández, Laura Reyes Aguirre, Rubén E Morán Galaviz, Clotilde Fuentes Orozco, Wenceslao G Ángeles Bueno, Fernando S Ramirez Marbello, Diego E Luna Acevedo, Michel Hernández Valadez, Ana L Bogurin Arellano, Luis R Ramírez-González, Bertha G Guzmán Ramírez, Eduardo Valtierra Robles, Ramona I Rojas García, José V Pérez Navarro, Edgar J Cortes Torres, David R Dominguez Solano, Alberto N Peón, Roque D Lincona Menindez, Rozana Reyes Gamez, Maria C Paz Muñoz, Orimisan Belie, Victoria Adeleye, Adesoji Ademuyiwa, Oluwafunmilayo Adeniyi, Opeyemi Akinajo, David Akinboyewa, Felix Alakaloko, Oluwole Atoyebi, Olanrewaju Balogun, Christopher Bode, Olumide Elebute, Francis Ezenwankwo, Adesiyakan Adedotun, George Ihediwa, Jubril Kuku, Oluwaseun Ladipo-Ajayi, Ayomide Makanjuola, Samuel Nwokocha, Olubunmi Ogein, Rufus Ojewola, Abraham Oladimeji, Thomas Olajide, Iyabo Alasi, Oluwaseun Oluseye, Justina Seyi-Olajide, Adaiah Soibi-Harry, Emmanuel Williams, Agbulu Moses Vincent, Nnamdi Jonathan Duru, Kenneth Uche Onyekachi, Christiana Ashley, Chinelo Victoria Mgbemena, Moyosoluwa Ojo, Olowu Oluyemisi, Iyabode Ikuewunmi, Adeoluwa Adebunmi, Edet Glory Bassey, Ephraim Okwudiri Ohazurike, Olayide Michael Amao, Osunwusi Benedetto Oluwaseun, Emily Doris, Olutola Stephen, Christianah Gbenga-Oke, Olawunmi Olayioye, Olowu Oluyemisi, Kayode Oluremi, Esther Abunimye, Christianah Oyegbola, Olayade Kayode, Adeola Ayoola Orowale, Omolara M Williams, Olufunmilade A Omisanjo, Omolara M Faboya, Zainab O Imam, Olabode A Oshodi, Yusuf A Oshodi, Ayokunle A Ogunyemi, Olalekan T Ajai, Francisca C Nwaenyi, Adewale O Adisa, Adewale A Aderounmu, Funmilola O Wuraola, Oludayo Sowande, Lukman Olajide Abdur-Rahman, Jibril Oyekunle Bello, Hadijat Olaide Raji, Nurudeen Abiola Adeleke, Saheed Abolade Lawal, Rafiat Tinuola Afolabi, Abdulwahab Lawal, Okechukwu Hyginus Ekwunife, Ochomma Amobi Egwuonwu, Chisom Faith Uche, Abubakar Bala A B Muhammad, Saminu S Muhammad, Idris Usman I U Takai, Mohammed A S Aliyu Salele, Onyekachi G Ukata, Mahmoud Kawu M K Magashi, Lawal Barau L B Abdullahi, Bello Abodunde B A Muideen, Khadija A Ado, Lofty-John Chukwuemeka L J C Anyawu, Samson Olori, Samuel A Sani, Olabisi O Osagie, Ndubuisi Mbajiekwe, Oseremen Aisuodionoe-Shadrach, Godwin O Akaba, Lazarus Ameh, Lazarus Ameh, Francis o Adebayo, Martins Uanikhoba, Felix O Ogbo, Nancy O Tabuanu, Taiwo A Lawal, Rukiyat A Abdus-Salam, Akinlabi E Ajao, Augustine O Takure, Omobolaji O Ayandipo, Hyginus O Ekwuazi, Olukayode Abayomi, Olatunji O Lawal, Solomon Olagunju, Kelvin I Egbuchulem, Sikiru Adekola Adebayo, Peter Elemile, Usang E Usang, Joseph E Udosen, Expo E Edet, Akan W Inyang, Edima M Olory, Gabriel U Udie, Godwin O Chiejina, Adams D Marwa, Faith J Iseh, Sunday A Ogbeche, Mary O Isa, Uchechukwu O Ezomike, Sebastian O Ekenze, Matthew I Eze, Emmanuel O Izuka, Jude K Ede, Vincent C Enemuo, Okezie M Mbadiwe, Ngozi G Mbah, Alphonsine Imanishimwe, Sosthene Habumuremyi, Faustin Ntirenganya, J C Allen Ingabire, Isaie Ncogoza, Emmanuel Munyaneza, Jean de Dieu Haragirimana, Christian Jean Urimubabo, Violette Mukanyange, Jeannette Nyirahabimana, Emmanuel Mutabazi, Christophe Mpirimbanyi, Olivier Mwenedata, Hope Lydia Maniraguha, Emelyne Izabiriza, Moses Dusabe, Job Zirikana, Francine Uwizeyimana, Josiane Mutuyimana, Elisee Rwagahirima, Alphonsine Imanishimwe, Ronald Tubasiime, Aphrodis Munyaneza, Sosthene Habumuremyi, Salathiel Kanyarukiko, Gibert Ndegamiye, Francine Mukaneza, Jean Claude Uwimana, Pierrine Nyirangeri, Deborah Mukantibaziyaremye, Aime Dieudonne Hirwa, Salomee Mbonimpaye, Piolette Muroruhirwe, Christine Mukakomite, Elysee Kabanda, Rachel Moore, Ncamsile Anthea Nhlabathi, Maria Fourtounas, Mary Augusta Adams, Gabriella Hyman, Hlengiwe Samkelisiwe Nxumalo, Nnosa Sentholang, Mmule Evelyn Sethoana, Mpho Nosipho Mathe, Zain Ally, Margot Flint, Bruce Biccard, Adesoji O Ademuyiwa, Adewale O Adisa, Aneel Bhangu, Peter Brocklehurst, Sohini Chakrabortee, Pollyanna Hardy, Ewen Harrison, J C Allen Ingabire, Parvez D Haque, Lawani Ismail, James Glasbey, Dhruva Ghosh, Frank Enoch Gyamfi, Elizabeth Li, Rachel Lillywhite, Antonio Ramos de la Medina, Rachel Moore, Laura Magill, Dion Morton, Dmitri Nepogodiev, Faustin Ntirenganya, Thomas Pinkney, Omar Omar, Joana Simoes, Donna Smith, Stephen Tabiri, Adesoji O Ademuyiwa, Lawani Ismail, Dhruva Ghosh, Antonio Ramos de la Medina, Rachel Moore, Faustin Ntirenganya, Stephen Tabiri, Adesoji Ademuyiwa, Aneel Bhangu, Felicity Brant, Peter Brocklehurst, Sohini Chakrabortee, Dhruva Ghosh, James Glasbey, Pollyanna Hardy, Ewen Harrison, Emily Heritage, Lawani Ismail, Karolin Kroese, Carmela Lapitan, Rachel Lillywhite, David Lissauer, Laura Magill, Antonio Ramos de la Medina, Punam Mistry, Mark Monahan, Rachel Moore, Dion Morton, Dmitri Nepogodiev, Faustin Ntirenganya, Omar Omar, Thomas Pinkney, Tracy Roberts, Donna Smith, Stephen Tabiri, Neil Winkles, Pollyanna Hardy, Omar Omar, Emmy Runigamugabo, Azmina Verjee, Pierre Sodonougbo, Pamphile Assouto, Michel Fiogbe, Houenoukpo Koco, Serge Metchinhoungbe, Hodonou Sogbo, Hulrich Behanzin, Djifid Morel Seto, Yannick Tandje, Sosthène Kangni, Cyrile Kpangon, Marcelin Akpla, Hugues Herve Chobli, Blaise Kovohouande, Gérard Agboton, Rene Ahossi, Raoul Baderha Ngabo, Nathan Bisimwa, Covalic Melic Bokossa Kandokponou, Mireille Dokponou, Francis Moïse Dossou, Corinne Dzemta, Antoine Gaou, Roland Goudou, Emmanuel Hedefoun, Sunday Houtoukpe, Felix Kamga, Eric Kiki-Migan, Souliath Lawani, Ismaïl Lawani, René Loko, Afissatou Moutaïrou, Pencome Ogouyemi, Fouad Soumanou, Pia Tamadaho, Mack-Arthur Zounon, Luke Aniakwo Adagrah, Bin Baaba Alhaji Alhassan, Mabel Pokuah Amoako-Boateng, Anthony Baffour Appiah, Alvin Asante-Asamani, Benedict Boakye, Samuel A Debrah, Donald Enti, Rahman Adebisi Ganiyu, Patience Koggoh, Richard Kpankpari, Isabella Naa M Opandoh, Meshach Agyemang Manu, Maison Patrick Opoku Manu, Samuel Mensah, Martin Tangnaa Morna, John Nkrumah, Michael Nortey, Emmanuel Owusu Ofori, Elizaberth Mercy Quartson, Esther Adjei-Acquah, Vera Agyekum, Eric Agyemang, Rebecca Adjeibah Akesseh, Forster Amponsah-Manu, Richard Ofosu-Akromah, Ato Oppong Acquah, Leslie Issa Adam-Zakariah, Esther Asabre, Ruby Acheampong Boateng, Barbara Koomson, Ataa Kusiwaa, Emmanuel Yaw Twerefour, James Ankomah, Frank Assah-Adjei, Anthony Appiah Boakye, Godfred Fosu, Godwin Serbeh, Kofi Yeboah Gyan, Isaac Omane Nyarko, Zelda Robertson, Ralph Armah, Christopher Asare, Delali Akosua Gakpetor, Victoria Sena Gawu, Ambe Obbeng, Doris Ofosuhene, Dorcas Osei-Poku, Diana Puozaa, Enoch Tackie, Arkorful Ebenezer Temitope, Regina Acquah, James Amoako, Akosua Dwamena Appiah, Mark Aseti, Charles Banka, Samuel Dadzie, Derick Essien, Frank Enoch Gyamfi, Romeo Hussey, Jemima Kwarteng, Naa Anyekaa Sowah, Grace Yeboah, Cynthia Yeboah, Kwame Gyambibi Addo, Enoch Appiah Akosa, Percy Boakye, Christian Larbi Coompson, Brian Gyamfi, Bismark Effah Kontor, Christian Kyeremeh, Ruth Manu, Elijah Mensah, Friko Ibrahim Solae, Gideon Kwasi Toffah, Dorcas Otuo Acheampong, Jane Acquaye, Michael Adinku, Kwabena Agbedinu, Anita Eseenam Agbeko, Emmanuel Gyimah Amankwa, Michael Amoah, George Amoah, Juliana Appiah, Joshua Arthur, Alex Ayim, Emmanuel Kafui Ayodeji, Jonathan Boakye-Yiadom, Edward Amoah Boateng, Charles Dally, Anthony Davor, Christian Kofi Gyasi-Sarpong, Naabo Nuhu Noel Hamidu, Iddrisu Haruna, Naa Kwarley, Agbenya Kobla Lovi, Boateng Nimako, Bertina Beauty Nyadu, Dominic Opoku, Anita Osabutey, Robert Sagoe, Samuel Tuffour, Yaa Tufour, Francis Akwaw Yamoah, Abiboye Cheduko Yefieye, Joseph Yorke, Nii Armah Adu-Aryee, Faisal Adjei, Erica Akoto, Elikem Ametefe, Joachim Kwaku Amoako, Godsway Solomon Attepor, George Darko Brown, Benjamin Fenu, Philemon Kwame Kumassah, David Olatayo Olayiwola, Theodore Wordui, Nelson Agboadoh, Fatao Abubakari, Cletus Ballu, Charles Gyamfi Barimah, Guy Casskey Boateng, Prosper Tonwisi Luri, Abraham Titigah, Frank Owusu, Raphael Adu-Brobbey, Christian Larbi Coompson, Abdul-Hamid Labaran, Junior Atta Owusu, Vivian Adobea, Amos Bennin, Fred Dankwah, Stanley Doe, Ruth Sarfo Kantanka, Ephraim Kobby, Kennedy Kofi Korankye Hanson Larnyor, Edwin Osei, Prince Yeboah Owusu, Clement Ayum Sie-Broni, Marshall Zume, Francis Atindaana Abantanga, Darling Ramatu Abdulai, Daniel Kwesi Acquah, Emmanuel Ayingayure, Imoro Osman, Sheba Kunfah, Gbana Limann, Shamudeen Alhassan Mohammed, Sheriff Mohammed, Yakubu Musah, Bernard Ofori, Emmanuel Abem Owusu, Abdul-Hafiz Saba, Anwar Sadat Seidu, Stephen Tabiri, Mustapha Yakubu, Edwin Mwintiereh Taang Yenli, Arun Gautham, Alice Hepzibah, Grace Mary, Deepak Singh, Dimple Bhatti, William Bhatti, Karan Bir, Swati Daniel, Tapasya Dhar, Jyoti Dhiman, Dhruva Ghosh, Sunita Goyal, Ankush, Goyal, Monika Hans, Parvez Haque, Samuel Konda, Anil Luther, Amit Mahajan, Shalini Makkar, Kavita Mandrelle, Vishal Michael, Partho Mukherjee, Reuben Rajappa, Prashant Singh, Atul Suroy, Ravinder Thind, Alen Thomas, Arti Tuli, Sreejith Veetil, Esther Daniel Mark Jesudason, Priyadarshini K, Latha Madankumar, Rohin Mittal, Ida Nagomy, Rajesh Selvakumar, Bharat Shankar, Moonish Sivakumar, Rajeevan Sridhar, Cecil Thomas, Devabalan Titus, Manisha Aggarwal, Parth Dhamija, Himani Gupta, Vinoth Kanna, Ashwani Kumar, Gurtaj Singh, Philip Alexander, Josy Thomas, Pradeep Zechariah, Amos Dasari, Priya Jacob, Elizabeth Kurien, Arpit Mathew, Danita Prakash, Anju Susan, Rose Varghese, Rahul Alpheus, Ashish Choudhrie, Hemanth Kumar, Nitin Peters, Subrat Raul, Rajeev Sharma, Rakesh Vakil, Wenceslao Ángeles Bueno, Francisco Barbosa Camacho, Aldo Bernal Hernández, Ana Bogurin Arellano, Edgar Cortes Torres, Clotilde Fuentes Orozco, Erick González García de Rojas, Alejandro González Ojeda, Bertha Guzmán Ramírez, Michel Hernández Valadez, Diego Luna Acevedo, Rubén Morán Galaviz, Oscar Olvera Flores, José Pérez Navarro, Kevin Pintor Belmontes, Fernando Ramirez Marbello, Luis Ramírez-González, Laura Reyes Aguirre, Ramona Rojas García, Eduardo Valtierra Robles, Reyes Cervantes Ortiz, Gonzalo Hernandez Gonzalez, Rosa Hernandez Krauss, Luis Hernández Miguelena, Marco Hurtado Romero, Isaac Baltazar Gomez, Celina Cuellar Aguirre, Alejandro Cuevas Avendaño, Luis Dominguez Sansores, Hector Ortiz Mejia, Laura Urdapilleta Gomez del Campo, Claudia Caballero Cerdan, David Dominguez Solano, Rafael Toriz Garcia, Mariana Barreto Gallo, Ana Cortes Flores, Alejandro Gonzalez Ojeda, Monica Jimenez Velasco, Rozana Reyes Gamez, Roque Lincona Menindez, Alberto Navarrete Peón, Maria Paz Muñoz, Irán Irani Durán Sánchez, Diana Samantha González Vázquez, María José Martínez Lara, Laura Martinez Perez Maldonado, Alejandra Nayen Sainz de la Fuente, Antonio Ramos De la Medina, Lawal Abdullahi, Khadija Ado, Mohammed Aliyu, Lofty-John Anyanwu, Mahmoud Magashi, Abubakar Muhammad, Saminu Muhammad, Bello Muideen, Idris Takai, Onyekachi Ukata, Opeoluwa Adesanya, David Awonuga, Olushola Fasiku, Chidiebere Ogo, Moruf Abdulsalam, Abimbola Adeniran, Olalekan Ajai, Olukemi Akande, Kazeem Atobatele, Grace Eke, Omolara Faboya, Zainab Imam, Esther Momson, Francisca Nwaenyi, Ayokunle Ogunyemi, Mobolaji Oludara, Olufunmilade Omisanjo, Olabode Oshodi, Yusuf Oshodi, Yemisi Oyewole, Omotade Salami, Omolara Williams, Victoria Adeleye, Adesoji Ademuyiwa, Oluwafunmilayo Adeniyi, Opeyemi Akinajo, David Akinboyewa, Iyabo Alasi, Felix Alakaloko, Oluwole Atoyebi, Olanrewaju Balogun, Orimisan Belie, Christopher Bode, Andrew Ekwesianya, Olumide Elebute, Francis Ezenwankwo, Adedeji Fatuga, George Ihediwa, Adesola Jimoh, Jubril Kuku, Oluwaseun LadipoAjayi, Ayomide Makanjuola, Olayanju Mokwenyei, Samuel Nwokocha, Olubunmi Ogein, Rufus Ojewola, Abraham Oladimeji, Thomas Olajide, Oluwaseun Oluseye, Justina Seyi-Olajide, Adaiah Soibi-Harry, Aloy Ugwu, Emmanuel Williams, Ochomma Egwuonwu, Okechukwu Ekwunife, Victor Modekwe, Chukwuemeka Okoro, Chisom Uche, Kenneth Ugwuanyi, Chuka Ugwunne, Akeem Adeleke, Wilson Adenikinju, Olumide Adeniyi, Akinfolarin Adepiti, Adewale Aderounmu, Abdulhafiz Adesunkanmi, Adewale Adisa, Samuel Ajekwu, Olusegun Ajenjfuja, Jerrie Akindojutimi, Akinbolaji Akinkuolie, Olusegun Alatise, Olubukola Allen, Lukmon Amosu, Micheal Archibong, Olukayode Arowolo, Deborah Ayantona, Ademola Ayinde, Olusegun Badejoko, Tajudeen Badmus, Amarachukwu Etonyeaku, Emeka Igbodike, Omotade Ijarotimi, Adedayo Lawal, Fayowole Nana, Tunde Oduanafolabi, Olalekan Olasehinde, Olaniyi Olayemi, Stephen Omitinde, Owolabi Oni, Chigozie Onyeze, Ernest Orji, Adewale Rotimi, Abdulkadir Salako, Olufemi Solaja, Oluwaseun Sowemimo, Ademola Talabi, Mohammed Tajudeen, Funmilola Wuraola, Francis Adebayo, Oseremen Aisuodionoe-Shadrach, Godwin Akaba, Lazarus Ameh, Ndubuisi Mbajiekwe, Felix Ogbo, Samson Olori, Olabisi Osagie, Abu Sadiq, Samuel Sani, Nancy Tabuanu, Martins Uanikhoba, Godwin Chiejina, Ekpo Edet, Akan Inyang, Mary Isa, Faith Iseh, Adams Marwa, Sunday Ogbeche, Edima Olory, Gabriel Udie, Joseph Udosen, Usang Usang, Olukayode Abayomi, Rukiyat Abdus-Salam, Sikiru Adebayo, Akinlabi Ajao, Olanrewaju Amusat, Omobolaji Ayandipo, Kelvin Egbuchulem, Hyginus Ekwuazi, Peter Elemile, Taiwo Lawal, Olatunji Lawal, Solomon Olagunju, Peter Osuala, Bamidele Suleman, Augustine Takure, Lukman Abdur-Rahman, Nurudeen Adeleke, Muideen Adesola, Rafiat Afolabi, Sulaiman Agodirin, Isiaka Aremu, Jibril Bello, Saheed Lawal, Abdulwahab Lawal, Hadijat Raji, Olayinka Sayomi, Asimiyu Shittu, Jude Ede, Sebastian Ekenze, Vincent Enemuo, Matthew Eze, Uchechukwu Ezomike, Emmanuel Izuka, Okezie Mbadiwe, Ngozi Mbah, Uba Ezinne, Matthew Francis, Iweha Ikechukwu, Okoi Nnyonno, Philemon Okoro, Igwe Patrick, John Raphael, Oriji Vaduneme, Abhulimen Victor, Salathiel Kanyarukiko, Francine Mukaneza, Deborah Mukantibaziyaremye, Aphrodis Munyaneza, Gibert Ndegamiye, Ronald Tubasiime, Moses Dusabe, Emelyne Izabiriza, Hope Lydia Maniraguha, Christophe Mpirimbanyi, Josiane Mutuyimana, Olivier Mwenedata, Elisee Rwagahirima, Francine Uwizeyimana, Job Zirikana, Aime Dieudonne Hirwa, Elysee Kabanda, Salomee Mbonimpaye, Christine Mukakomite, Piolette Muroruhirwe, Georges Bucyibaruta, Gisele Juru Bunogerane, Sosthene Habumuremyi, Jean de Dieu Haragirimana, Alphonsine Imanishimwe, J C Allen Ingabire, Violette Mukanyange, Emmanuel Munyaneza, Emmanuel Mutabazi, Isaie Ncogoza, Faustin Ntirenganya, Jeannette Nyirahabimana, Christian Urimubabo, Mary Augusta Adams, Richard Crawford, Chikwendu Jeffrey Ede, Maria Fourtounas, Gabriella Hyman, Zafar Khan, Morapedi Kwati, Mpho Nosipho Mathe, Rachel Moore, Ncamsile Anthea Nhlabathi, Hlengiwe Samkelisiwe Nxumalo, Paddy Pattinson, Nnosa Sentholang, Mmule Evelyn Sethoana, Maria Elizabeth Stassen, Laura Thornley, Paul Wondoh, Cheryl Birtles, Mathete Ivy, Cynthia Mbavhalelo, Zain Ally, Abdus-sami Adewunmi, Jonathan Cook, David Jayne, Soren Laurberg, Julia Brown, Simon Cousens, Neil Smart

## Abstract

**Introduction:**

Telemedicine is being adopted for postoperative surveillance but requires evaluation for efficacy. This study tested a telephone Wound Healing Questionnaire (WHQ) to diagnose surgical site infection (SSI) after abdominal surgery in low- and middle-income countries.

**Method:**

A multi-centre, international, prospective study was embedded in the FALCON trial; a factorial RCT testing measures to reduce SSI in seven low- and middle-income countries (NCT03700749). It was conducted according to a pre-registered protocol (SWAT126) and reported according to STARD guidelines. The reference test was in-person review by a trained clinician at 30 postoperative days according to US Centres for Disease Control criteria. The index test was telephone administration of an adapted WHQ at 27 to 30 postoperative days by a researcher blinded to the outcome of in-person review. The sum of item response scores generated an overall score between 0 and 29. The primary outcome was the diagnostic accuracy of the WHQ, defined as the proportion of SSI correctly identified by the telephone WHQ, and summarized using the area under the receiving operator characteristic curve (AUROC) and diagnostic test accuracy statistics.

**Results:**

Patients were included from three upper-middle income (396 patients, 13 hospitals), three lower-middle income (746 patients, 19 hospitals), and one low-income country (54 patients, 4 hospitals). 90.3% (1088 of 1196) patients were successfully contacted. Those with non-midline incisions (adjusted odds ratio: 0.36, 95% c.i. 0.17 to 0.73, *P*=0.005) or a confirmed diagnosis of SSI on in-person assessment (odds ratio: 0.42, 95% c.i. 0.20 to 0.92, *P*=0.006) were harder to reach. The questionnaire correctly discriminated between most patients with and without SSI (AUROC 0.869, 95% c.i. 0.824 to 0.914), which was consistent across subgroups. A representative cut-off score of ≥4 displayed a sensitivity of 0.701 (0.610-0.792), specificity of 0.911 (0.878-0.943), positive predictive value of 0.723 (0.633-0.814) and negative predictive value of 0.901 (0.867-0.935).

**Conclusion:**

SSI can be diagnosed using a telephone questionnaire (obviating in-person assessment) in low resource settings.

## Teaser

This collaborative international study within a trial demonstrated feasibility and high diagnostic accuracy of a telephone pathway for remote post-discharge wound assessment after major abdominal surgery for patients in 7 low and middle-income countries.

## Introduction

Surgical-site infection (SSI) is the most common postoperative complication, with a cross-societal impact on patients, communities, and economies worldwide^1–3^. It has been recognized as the highest-priority research area in global surgery^4^ and SSI prevention is the subject of several ongoing global randomized trials and quality improvement programmes^5,6^.

Whilst some SSI occurs while patients are in-hospital, the majority occurs after discharge^7^. Post-discharge surveillance of SSI is therefore considered a key quality marker in wound infection research^8^. The accepted reference standard of assessment for SSI during the 30 days after surgery is an in-person review according to US Centers for Disease Control and Prevention (CDC) criteria^9^. However, in-person assessment is labour and time intensive, and requires patients to take additional time off work and incur costs of travel. This is particularly challenging in resource-limited environments, where there are shortages in the surgical workforce and patients are already at risk of catastrophic expenditure as a direct and indirect result of their surgical care^10^.

Remote follow-up methods were rapidly adopted during the SARS-CoV-2 pandemic to reduce the risk of in-hospital transmission and to conserve resources for surges in COVID-19 admissions and to address elective surgical backlogs^11,12^. Whilst telephone follow-up may offer greater efficiency and cost savings, missed SSI events may lead to patient harm directly through care delays or indirectly through inefficiencies in SSI-prevention research^13^.

The overall aim of this study was to evaluate the feasibility and diagnostic accuracy of telephone administration of a Wound Healing Questionnaire (WHQ) for remote detection of SSI after abdominal surgery in low- and middle-income countries (LMICs). The results of this study will inform efficient design and the conduct of future randomized trials and postoperative surveillance programmes.

## Methods

This was a prospective, multicentre, international, non-randomized cohort Study Within A Trial (SWAT) exploring the feasibility and accuracy of remote follow-up pathways for SSI assessment (TALON). It was embedded within a pragmatic, multicentre, factorial randomized controlled trial testing measures to reduce SSI in LMICs (FALCON). FALCON was a stratified, pragmatic, multicentre, 2 × 2 factorial trial testing two measures (skin preparation and antimicrobial sutures) to reduce superficial or deep-skin infection after abdominal surgery for 5788 patients in 54 hospitals in 7 LMICs (NCT03700749)^14^. In this trial, superiority of the intervention groups over the control group, was not demonstrated overall, alone or in combination, or in any pre-planned subgroup^15^.

The study protocol was pre-registered online on the MRC Hubs for Trial Methodology Research SWAT store database^16^ (Queen’s University Belfast) (SWAT ID 126) and published in *Trials*^17^. This report was prepared with reference to the Statistical Analyses and Methods in the Published Literature (‘SAMPL’) guidelines^18^, the methodology standards of the Patient-Centred Outcomes Research Institute (‘PCORI’)^19^, the STARD guidelines for diagnostic test accuracy studies^20^, and the COSMIN guidelines for patient-reported outcomes research^21^.

### Ethics approval and consent to participate

A protocol amendment to embed TALON in the host trial (FALCON) was obtained from the University of Birmingham International Ethics Committee (Reference: ERN_18-0230A). All individual participating countries obtained local or national ethical approval for TALON in accordance with local protocols. Written (or fingerprint) informed consent to participate was obtained from all participants.

### Inclusion and exclusion criteria

Consecutive adult patients (greater than 16 years old) recruited to the FALCON trial between 10 December 2018 and 6 September 2020 were eligible for recruitment to TALON. Any centre participating in FALCON was eligible to participate. Centres were given flexibility to include patients over different date ranges depending on their local capacity and infrastructure, so long as sampling was consecutive. This included a broad range of abdominal operations with a predicted clean/clean-contaminated, contaminated, or dirty operating field and a planned skin incision of greater than 5 cm, for benign disease, malignant disease, trauma, and obstetric indications. This aimed to be representative of patients undergoing emergency or elective surgery in LMICs. Patients who were unlikely to be contactable for the 30-day follow-up were excluded from the FALCON trial. Patients with a missing FALCON 30-day follow-up assessment (either in person or by telephone) or who died before 30 days after surgery were excluded from analysis in this study.

### Reference and index diagnostic tests

The reference diagnostic test for SSI during the 30 days after surgery was in-person review according to US CDC criteria^9^. This is widely accepted as a quality standard in SSI research and has been used by most major international RCTs^8^, and included both in-hospital and post-discharge diagnosis of SSI. A full description of the definition used in the FALCON trial is available in *Appendix B*.

The index diagnostic test under evaluation was a telephone-administered Bluebelle WHQ^17^, adapted for use in LMICs. The WHQ was originally developed and validated in the UK (English language) to assess post-discharge infections after abdominal surgery^22,23^. The WHQ was designed to be completed either by healthcare professionals or patients^24^, and, as such, has been described as a ‘universal-reporter’ outcome measure (‘UROM’)^25^. In a UK validation study, the WHQ demonstrated good reliability and high sensitivity and specificity when discriminating between SSI and no SSI in comparison with an in-person US CDC assessment^22,23^. The original WHQ was adapted for use in global surgery trials for use across language and resource settings using recognized practices for translating outcome measures, reported in detail separately and summarized in *Appendix C*. Briefly, this involved two phases. First, an adaptation phase with structured interviewing and translatability assessment with local researchers, triangulated with analysis of the scaling and measurement properties of the WHQ in pilot data, and informed by Rasch unidimensional measurement modelling^26,27^. Second, a nine-phase translation phase for each language of delivery following Mapi recommendations^26^. In the adapted version of the WHQ, the response options and subsequent scoring were also modified. Here, ‘WHQ’ refers to this adapted questionnaire. In the adapted WHQ scale, items assessing SSI signs and symptoms were scored between 0 and 2 (not at all, a little, and a lot) and items assessing wound care interventions were scored between 0 and 1 (no and yes). These were added together to create an overall score between 0 and 29. The full adapted WHQ instrument is available in *Appendix D*.

According to the TALON protocol, the WHQ was to be administered over the telephone by a non-surgeon (consultant, attending, or equivalent) researcher (that is, a junior doctor, research nurse, or other non-clinical member of staff) between 27 and 30 days after surgery (that is before the reference diagnostic test) as the index diagnostic test in this study. The researcher administering the questionnaire was independent of the 30-day wound assessment in the FALCON trial (that is each was blinded to the reference and index test result respectively) and underwent standardized training from the Study Management Group (‘SMG’). Details of monitoring and quality assurance can be found in *Appendix E*. Pathways for questionnaire administration were co-designed between patient partners, site investigators, and research managers. Methodological adaptations for delivery during the SARS-CoV-2 pandemic are summarized in *Appendix F*.

### FALCON trial follow-up

Due to personal (mobility, deterioration, and psychological) and environmental (cost, transport links, and SARS-CoV-2 transmission risk) reasons, not all patients were able to return to hospital for the reference test assessment in the FALCON trial (in-person 30-day follow-up according to US CDC criteria). Eligible patients were therefore categorized according to their corresponding FALCON trial follow-up as having: in-person FALCON trial follow-up; or telephone FALCON trial follow-up only. Only patients with in-person FALCON trial follow-up were considered to have had a reference test completed.

### Outcomes

The primary outcome measure was the diagnostic accuracy of the telephone WHQ in identification of SSI up to 30 days after surgery. The performance of the test was summarized using discrimination (area under the receiving operator characteristic curve (AUROC)) and diagnostic test accuracy statistics (accuracy, sensitivity, specificity, positive predictive value, and negative predictive value).

The secondary outcome measure was the feasibility of the telephone WHQ follow-up, which was characterized using: telephone contact (successful contact of a patient on the telephone by the research team); return rate (successful completion of the WHQ where telephone contact was made); patient satisfaction (the patient’s self-reported satisfaction with the telephone WHQ follow-up); and data completion rate (complete item response data). The estimated ‘retention benefit’ of using a telephone pathway *versus* in-person follow-up was estimated as the difference between the proportion of patients for whom the telephone WHQ was successfully completed and/or a telephone FALCON trial follow-up was completed, and the proportion for whom an in-person FALCON trial follow-up was completed^28^.

### Sample size

A range of sample sizes and their impact on the precision of estimates of sensitivity and specificity, from 95% confidence intervals, were investigated. Calculations assumed a 30-day SSI prevalence of 21.0% using the binomial exact formula and were pre-specified^29^ (*Appendix G*). Sample sizes were adjusted to allow for 15.0% predicted loss to follow-up from the FALCON trial, and 15.0% of patients predicted not to undergo in-person follow-up. In patients with successful telephone contact and in-person FALCON trial follow-up, 87 events and 325 non-events would estimate sensitivity of 0.92 (95% c.i. 0.84 to 0.97) and specificity of 0.95 (95% c.i. 0.92 to 0.97). A target of 100 or more patients per country were recommended to be recruited; however, no minimum or maximum sample size limitations per site or per country were imposed.

### Telephone Wound Healing Questionnaire administration pathway

Data were collected about the pathway for telephone WHQ follow-up to describe the variability in administration across contexts, including: questionnaire translation (pre-translated questionnaire/ad hoc, translated by questionnaire administrator/ad hoc, translated by formal translator); language of delivery; telephone owner (patient themselves/healthcare worker/friend or relative/other); telephone type (landline/mobile telephone with a camera/mobile telephone without a camera); questionnaire administrator (consultant (doctor)/junior doctor/research nurse/other non-clinical); and duration (min).

### Statistical analysis

A full statistical analysis plan (SAP) was published online on 8 March 2021^30^. Any changes from the SAP are summarized in *Appendix H*. All analyses were performed using R Studio version 4.1.1 (R Foundation for Statistical Computing, Vienna, Austria) packages: tidyverse, finalfit, reportROC, predictr, and bcROCcurve. Country income level was defined according to the World Bank’s 2018 definitions and countries classified into upper-middle-income countries, lower-middle-income countries, or low-income countries based on annual gross domestic product per capita ($). The overall rate of missing data was anticipated to be low. A sensitivity analysis for the primary validation model was pre-planned to be performed with missing item response data imputed using Multiple Imputation by Chained Equations if the level of missingness was above 5% overall (that is per questionnaire) or for any individual item. Data from patients with in-person or telephone FALCON trial follow-up only were included in the evaluation of feasibility outcome measures. Data only from patients with in-person and telephone FALCON trial follow-up (that is both the reference and index test available) were included in the evaluation of diagnostic accuracy. A potential risk of partial verification bias by including only patients with in-person FALCON trial follow-up in the diagnostic accuracy analysis was identified a priori and addressed in a sensitivity analysis.

Baseline demographics and feasibility outcomes were presented overall, by country, by patient home location, and by FALCON trial follow-up group. Distributions of continuous variables were visually inspected for normality. Differences between these groups were explored using Student’s *t* test for normal data and the Mann–Whitney *U* test for non-normal data. The chi-squared test was used for categorical data, with Fisher’s exact modification, where required. The proportion of patients included by FALCON trial follow-up group over the study interval was summarized graphically. An exploratory mixed-effects binary regression model was used to explore the factors associated with successful telephone contact, with patients nested within countries. The causal pathway for telephone contact was mapped and patient-, disease-, operation-, and location-specific factors were selected a priori for inclusion in risk adjustment. Cross-tabulations of the reference test diagnosis (‘no SSI’ or ‘SSI’) against a binary outcome variable derived from the total score of the index test (created by a cut-off score; a WHQ total score of less than or equal to specified values between 1 and 10) were presented. The total WHQ score was examined against the reference test to evaluate the performance of the WHQ in discriminating between individuals with and without SSI. A receiver operating characteristic (ROC) curve was plotted showing test performance across all thresholds, with overall discrimination presented as the AUROC, with 95% confidence intervals, overall and across several subgroups. Diagnostic test accuracy statistics (accuracy, sensitivity, specificity, positive predictive value, and negative predictive value) were presented at statistically ‘optimized’ cut-off points (using Youden’s index, which maximizes the sum of sensitivity and specificity) and at several other cut-off points ‘to rule in’ (that is score greater than or equal to X) or ‘rule out’ (that is score less than or equal to X) to support clinical application.

Several sensitivity analyses were performed for the primary model:

- To allow flexibility during the SARS-CoV-2 pandemic, administration of the WHQ was permitted after FALCON follow-up. The effect of a longer duration after surgery between the WHQ and telephone assessment was explored in a sensitivity analysis including both per-protocol and out-of-protocol patients.
- To evaluate the diagnostic accuracy of the WHQ in post-discharge SSI diagnosis only, a second analysis excluded patients with an in-hospital, pre-discharge SSI diagnosis.
- To address a risk of partial verification bias, an inverse probability weighted (IPW) sensitivity analysis was conducted for the primary model. In brief, this bias represents a missing data problem, where the reference test is missing for a subset of the sample^31^. Under an assumption of missing data at random (‘MAR’), the IPW method weights each observation in the verified sample by the inverse of the probability of verification to provide a corrected estimate of sensitivity and specificity. The estimated probability of verification is then obtained using a logistic regression model^32,33^.

Subgroups included urban *versus* rural home location, upper-middle-income countries *versus* lower-middle-income countries *versus* low-income countries, patient age greater than 60 years *versus* less than or equal to 60 years, and elective *versus* emergency surgery, which were pre-specified, and pre-translated questionnaire *versus* ad hoc translation and no reoperation (mild SSI only), which were added post-hoc for exploratory analysis. Calibration of the WHQ was presented as the proportion of patients with an SSI diagnosis in the reference test at each WHQ point score interval.

### Community engagement and involvement

The aim of community engagement and involvement (CEI) in this study was to optimize the pathway for telephone WHQ administration to ensure cultural and contextual acceptability and to maximize both the telephone contact rate and questionnaire completion rate. Patient and community partners were involved in study prioritization, design, steering, and reporting using three methods. First, through direct involvement in the SMG. Second, through a UK-based advisory group with expatriate partners from collaborating countries. Third, an extended network of patient and community partners were consulted through the NIHR Unit on Global Surgery network. CEI in this study was reported according to the GRIPP2 short form^34^.

## Results

**Fig. 1:**
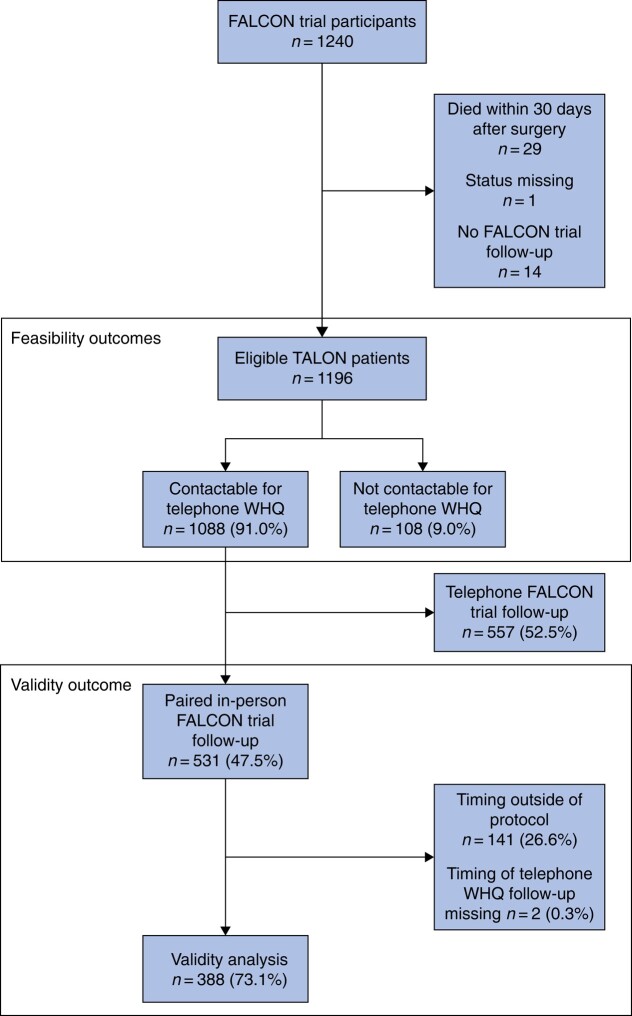
Study flow chart WHQ, Wound Healing Questionnaire.

Overall, 1240 patients were included, with telephone WHQ follow-up attempted, of whom 29 patients had died by 30 days after surgery, the status was missing for 1 patient, and 14 patients had no FALCON trial follow-up. A total of 1196 patients were therefore eligible for inclusion in the analyses (*Fig. 1*). Patients were from three upper-middle-income countries (396 patients in 13 hospitals), three lower-middle-income countries (746 patients in 19 hospitals), and one low-income country (54 patients in 4 hospitals). The countries contributing the largest numbers of patients were Ghana (532 of 1196 patients; 44.5%), Mexico (216 of 1196 patients; 18.1%), and India (120 of 1196 patients; 10.0%) (*Table S1*). A total of 209 of 1196 patients (17.5%) had an SSI diagnosis within 30 days after surgery in the FALCON trial. A comparison of patients included in the TALON study *versus* the FALCON trial overall is presented in *Table S2*. Of note, there were fewer patients undergoing elective surgery, fewer female patients, more intermediate/minor operations, and more contaminated/dirty surgery in TALON than in FALCON overall.

### Feasibility outcomes

**Fig. 2:**
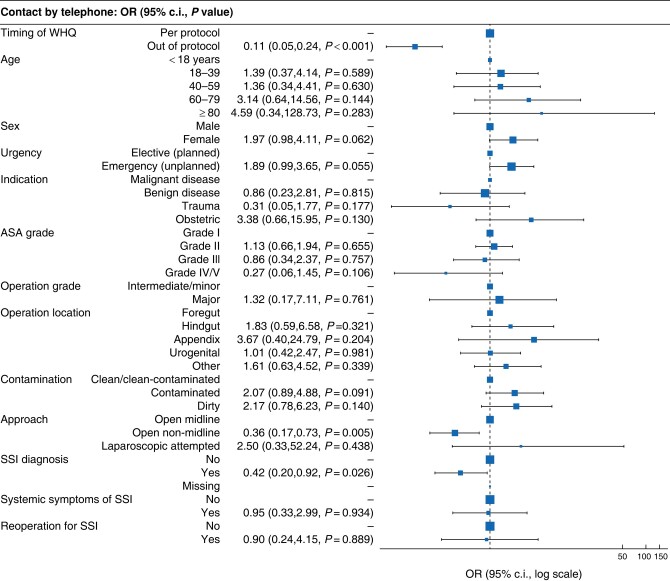
Factors associated with successful telephone contact in a multivariable model A lower OR conveyed a lower likelihood of telephone contact successfully being made by telephone to complete the TALON questionnaire. Full model presented in *Table S4*. WHQ, Wound Healing Questionnaire; SSI, surgical-site infection.

Baseline demographics grouped by whether telephone contact was made or not are presented in *Table 1*. Overall, the telephone contact rate was high at 90.3% (1088 of 1196 patients), with 9.7% (108 of 1196 patients) lost to follow-up, with some variability by country (*Table S3*). The WHQ was completed for all but one patient where successful contact was made (1087 of 1088 patients; 99.9%). The rate of telephone contact reduced as the time from date of surgery increased (*Fig. S1*). The most significant factor associated with lower odds of telephone contact in the multivariable model was time from surgery (*Fig. 2* and *Table S4*). Importantly, patients with non-midline incisions (adjusted odds ratio 0.36 (95% c.i. 0.17 to 0.73); *P* = 0.005) or with a confirmed reference test diagnosis of SSI (OR 0.42 (95% c.i. 0.20 to 0.92); *P* = 0.006) were less likely to be contactable. Where data were available, most patients were followed up with one (267 of 560 patients; 47.7%) or two to three (185 of 560 patients; 33.0%) attempts at telephone follow-up (data missing for 636 patients). Patients overall felt very satisfied (393 of 550 patients; 71.5%) or satisfied (152 of 550 patients; 27.6%) with undergoing telephone WHQ follow-up (data missing for 646 patients).

**Table 1:** Baseline demographics of patients with and without successful telephone contact (*n* = 1196)

Factor	Levels	Successful telephone contact		Total	*P* for difference
		No (*n* = 108)	Yes (*n* = 1088)		
Age (years)	<18	4 (3.7)	90 (8.3)	94 (7.9)	0.213
	18–39	64 (59.3)	606 (55.7)	670 (56.0)	
	40–59	32 (29.6)	262 (24.1)	294 (24.6)	
	60–79	7 (6.5)	115 (10.6)	122 (10.2)	
	≥80	1 (0.9)	15 (1.4)	16 (1.3)	
Sex	Male	33 (30.6)	582 (53.5)	615 (51.4)	<0.001
	Female	75 (69.4)	506 (46.5)	581 (48.6)	
Known diabetes	Yes	7 (6.5)	33 (3.0)	40 (3.3)	0.105
	No	101 (93.5)	1055 (97.0)	1156 (96.7)	
HIV status	Known negative	25 (23.1)	319 (29.3)	344 (28.8)	0.395
	Known positive	2 (1.9)	21 (1.9)	23 (1.9)	
	Status not known	81 (75.0)	748 (68.8)	829 (69.3)	
Smoking status	Never smoked	93 (86.1)	996 (91.5)	1089 (91.1)	0.006
	Ex-smoker	13 (12.0)	53 (4.9)	66 (5.5)	
	Current smoker	2 (1.9)	39 (3.6)	41 (3.4)	
Urgency	Elective (planned)	71 (65.7)	197 (18.1)	268 (22.4)	<0.001
	Emergency (unplanned)	37 (34.3)	891 (81.9)	928 (77.6)	
Indication	Malignant disease	5 (4.6)	80 (7.4)	85 (7.1)	0.006
	Benign disease	73 (67.6)	834 (76.7)	907 (75.8)	
	Trauma	4 (3.7)	40 (3.7)	44 (3.7)	
	Obstetric	26 (24.1)	133 (12.2)	159 (13.3)	
	Missing	0 (0.0)	1 (0.1)	1 (0.1)	
Operation location	Foregut	43 (39.8)	272 (25.0)	315 (26.3)	<0.001
	Hindgut	6 (5.6)	106 (9.7)	112 (9.4)	
	Appendix	8 (7.4)	324 (29.8)	332 (27.8)	
	Urogenital	42 (38.9)	162 (14.9)	204 (17.1)	
	Other	9 (8.3)	219 (20.1)	228 (19.1)	
	Missing	0 (0.0)	5 (0.5)	5 (0.4)	
ASA grade	I	41 (38.0)	519 (47.7)	560 (46.8)	0.003
	II	55 (50.9)	415 (38.1)	470 (39.3)	
	III	8 (7.4)	142 (13.1)	150 (12.5)	
	IV/V	4 (3.7)	11 (1.0)	15 (1.3)	
	Missing	0 (0.0)	1 (0.1)	1 (0.1)	
WHO checklist	Yes	105 (97.2)	1006 (92.5)	1111 (92.9)	0.101
	No	3 (2.8)	82 (7.5)	85 (7.1)	
Operation grade	Intermediate/minor	10 (9.3)	350 (33.0)	360 (30.8)	<0.001
	Major	98 (90.7)	711 (67.0)	809 (69.2)	
Contamination	Clean/clean-contaminated	85 (78.7)	322 (29.6)	407 (34.0)	<0.001
	Contaminated	14 (13.0)	399 (36.7)	413 (34.5)	
	Dirty	9 (8.3)	365 (33.5)	374 (31.3)	
	Missing	0 (0.0)	2 (0.2)	2 (0.2)	
Approach	Open midline	26 (24.1)	707 (65.0)	733 (61.3)	<0.001
	Open non-midline	81 (75.0)	371 (34.1)	452 (37.8)	
	Laparoscopic attempted	1 (0.9)	9 (0.8)	10 (0.8)	
	Missing	0 (0.0)	1 (0.1)	1 (0.1)	
Stoma formation	Yes	4 (3.7)	55 (5.1)	59 (4.9)	0.703
	No	103 (95.4)	1025 (94.2)	1128 (94.3)	
	Missing	1 (0.9)	8 (0.7)	9 (0.8)	

Values are *n* (%).

Telephone WHQ administration was performed across diverse settings and patient groups, in 22 languages and 36 hospitals (*Table 2*). Overall, 65.0% of contactable patients (707 of 1087) lived in urban settings and 34.9% of contactable patients (380 of 1087) lived in rural settings; data missing for 1 patient. In addition, 64.4% of contactable patients (701 of 1087) received the call using their own telephone, whereas 33.7% of contactable patients (367 of 1087) received the call using a family member’s telephone. A total of 699 patients (64.2%) used a smartphone with video capability. Importantly, the WHQ was mainly delivered by non-consultant (attending) grade researchers (other doctor for 367 patients (33.7%), research nurse for 327 patients (30.1%), and other non-clinical for 385 patients (35.4%)) and largely took less than 20 min to complete for 96.0% (528 of 550 patients; data missing for 538 patients). There were several differences in the implementation of telephone WHQ follow-up across the participating countries (*Table 2*).

**Table 2:** Follow-up for patients contactable for telephone WHQ (*n* = 1088)

Factor	Levels	Country							Total (*n* = 1088)
		Ghana (*n* = 520)	South Africa (*n* = 54)	India (*n* = 120)	Benin (*n* = 100)	Mexico (*n* = 130)	Rwanda (*n* = 54)	Nigeria (*n* = 111)	
Questionnaire translation	Pre-translated questionnaire	150 (28.8)	9 (16.7)	14 (11.7)	87 (87.0)	120 (93.0)	43 (79.6)	65 (58.6)	424 (39.0)
	Ad hoc, translated from English by questionnaire administrator	368 (70.8)	30 (55.6)	101 (84.2)	13 (13.0)	9 (7.0)	11 (20.4)	46 (41.4)	579 (53.2)
	Ad hoc, translated from English by formal translator	0 (0.0)	1 (1.9)	5 (4.2)	0 (0.0)	0 (0.0)	0 (0.0)	0 (0.0)	6 (0.6)
	Missing	2 (0.4)	14 (25.9)	0 (0.0)	0 (0.0)	0 (0.0)	0 (0.0)	0 (0.0)	79 (7.3)
Language of delivery	English	79 (15.2)	24 (44.4)	5 (4.2)	0 (0.0)	0 (0.0)	0 (0.0)	65 (58.6)	173 (15.9)
	Fante	20 (3.8)	0 (0.0)	0 (0.0)	0 (0.0)	0 (0.0)	0 (0.0)	0 (0.0)	20 (1.8)
	Fon	0 (0.0)	0 (0.0)	0 (0.0)	6 (6.0)	0 (0.0)	0 (0.0)	0 (0.0)	6 (0.6)
	Goun	0 (0.0)	0 (0.0)	0 (0.0)	6 (6.0)	0 (0.0)	0 (0.0)	0 (0.0)	6 (0.6)
	Hausa	0 (0.0)	0 (0.0)	0 (0.0)	0 (0.0)	0 (0.0)	0 (0.0)	2 (1.8)	2 (0.2)
	Igbo	0 (0.0)	0 (0.0)	0 (0.0)	0 (0.0)	0 (0.0)	0 (0.0)	16 (14.4)	16 (1.5)
	Malayalam	0 (0.0)	0 (0.0)	2 (1.7)	0 (0.0)	0 (0.0)	0 (0.0)	0 (0.0)	2 (0.2)
	Sotho	0 (0.0)	10 (18.5)	0 (0.0)	0 (0.0)	0 (0.0)	0 (0.0)	0 (0.0)	10 (0.9)
	Swati	0 (0.0)	1 (1.9)	0 (0.0)	0 (0.0)	0 (0.0)	0 (0.0)	0 (0.0)	1 (0.1)
	Telegu	0 (0.0)	0 (0.0)	1 (0.8)	0 (0.0)	0 (0.0)	0 (0.0)	0 (0.0)	1 (0.1)
	Tswana	0 (0.0)	3 (5.6)	0 (0.0)	0 (0.0)	0 (0.0)	0 (0.0)	0 (0.0)	3 (0.3)
	Xhosa	0 (0.0)	3 (5.6)	0 (0.0)	0 (0.0)	0 (0.0)	0 (0.0)	0 (0.0)	3 (0.3)
	Yoruba	0 (0.0)	0 (0.0)	0 (0.0)	0 (0.0)	0 (0.0)	0 (0.0)	28 (25.2)	28 (2.6)
	Zulu	0 (0.0)	13 (24.1)	0 (0.0)	0 (0.0)	0 (0.0)	0 (0.0)	0 (0.0)	13 (1.2)
	Dagbani	56 (10.8)	0 (0.0)	0 (0.0)	0 (0.0)	0 (0.0)	0 (0.0)	0 (0.0)	56 (5.1)
	French	0 (0.0)	0 (0.0)	0 (0.0)	88 (88.0)	0 (0.0)	0 (0.0)	0 (0.0)	88 (8.1)
	Hindi	0 (0.0)	0 (0.0)	69 (57.5)	0 (0.0)	0 (0.0)	0 (0.0)	0 (0.0)	69 (6.3)
	Kinyarwanda	0 (0.0)	0 (0.0)	0 (0.0)	0 (0.0)	0 (0.0)	54 (100.0)	0 (0.0)	54 (5.0)
	Punjabi	0 (0.0)	0 (0.0)	20 (16.7)	0 (0.0)	0 (0.0)	0 (0.0)	0 (0.0)	20 (1.8)
	Spanish	0 (0.0)	0 (0.0)	0 (0.0)	0 (0.0)	129 (100.0)	0 (0.0)	0 (0.0)	129 (11.9)
	Tamil	0 (0.0)	0 (0.0)	23 (19.2)	0 (0.0)	0 (0.0)	0 (0.0)	0 (0.0)	23 (2.1)
	Twi	364 (70.0)	0 (0.0)	0 (0.0)	0 (0.0)	0 (0.0)	0 (0.0)	0 (0.0)	364 (33.5)
	Missing	1 (0.2)	0 (0.0)	0 (0.0)	0 (0.0)	0 (0.0)	0 (0.0)	2 (1.8)	1 (0.1)
Phone owner	Patient themselves	335 (64.4)	43 (79.6)	44 (36.7)	84 (84.0)	91 (70.5)	23 (42.6)	81 (73.0)	701 (64.4)
	Healthcare worker	0 (0.0)	1 (1.9)	1 (0.8)	0 (0.0)	0 (0.0)	3 (5.6)	0 (0.0)	5 (0.5)
	Friend or relative	183 (35.2)	9 (16.7)	74 (61.7)	16 (16.0)	38 (29.5)	20 (37.0)	27 (24.3)	367 (33.7)
	Other	1 (0.2)	1 (1.9)	1 (0.8)	0 (0.0)	0 (0.0)	8 (14.8)	3 (2.7)	14 (1.3)
	Missing	1 (0.2)	0 (0.0)	0 (0.0)	0 (0.0)	0 (0.0)	0 (0.0)	0 (0.0)	1 (0.1)
Phone type	Landline telephone	0 (0.0)	0 (0.0)	1 (0.8)	0 (0.0)	12 (9.3)	0 (0.0)	0 (0.0)	13 (1.2)
	Mobile telephone (with a camera)	295 (56.7)	41 (75.9)	94 (78.3)	77 (77.0)	104 (80.6)	6 (11.1)	82 (73.9)	699 (64.2)
	Mobile telephone (without a camera)	225 (43.3)	13 (24.1)	25 (20.8)	23 (23.0)	13 (10.1)	48 (88.9)	29 (26.1)	376 (34.6)
Questionnaire administrator	Consultant (doctor)	0 (0.0)	0 (0.0)	0 (0.0)	0 (0.0)	6 (4.7)	0 (0.0)	2 (1.8)	8 (0.7)
	Other doctor	175 (33.7)	0 (0.0)	2 (1.7)	100 (100.0)	86 (66.7)	0 (0.0)	4 (3.6)	367 (33.7)
	Research nurse	104 (20.0)	48 (88.9)	102 (85.0)	0 (0.0)	0 (0.0)	46 (85.2)	27 (24.3)	327 (30.1)
	Other	240 (46.2)	6 (11.1)	16 (13.3)	0 (0.0)	37 (28.7)	8 (14.8)	78 (70.3)	385 (35.4)
	Missing	1 (0.2)	0 (0.0)	0 (0.0)	0 (0.0)	0 (0.0)	0 (0.0)	0 (0.0)	1 (0.1)
Duration of telephone assessment (min)*	<10	222 (42.7)	43 (79.6)	37 (30.8)	0 (0.0)	9 (7.0)	8 (14.8)	56 (50.5)	375 (34.5)
	11–20	66 (12.7)	11 (20.4)	7 (5.8)	0 (0.0)	2 (1.6)	29 (53.7)	38 (34.2)	153 (14.1)
	21–30	1 (0.2)	0 (0.0)	0 (0.0)	0 (0.0)	0 (0.0)	15 (27.8)	1 (0.9)	17 (1.6)
	>30	0 (0.0)	0 (0.0)	0 (0.0)	0 (0.0)	0 (0.0)	2 (3.7)	3 (2.7)	5 (0.5)
	Missing	231 (44.4)	0 (0.0)	76 (63.3)	100 (100.0)	118 (91.5)	0 (0.0)	13 (11.7)	538 (49.4)

Values are *n* (%). *Question added after pilot phase in response to Community Engagement and Involvement group feedback, so not available for patients recruited in the pilot phase.

### Patterns of FALCON trial follow-up

An overview of the grouping of patients included in this study is included in *Fig. 1*.

Of the 1209 patients who were contactable for telephone WHQ follow-up, 531 (47.5%) had a FALCON trial in-person follow-up. The proportion of patients with in-person follow-up over the study interval is shown in *Fig. S2*. Having a telephone follow-up pathway (telephone FALCON trial follow-up and/or telephone WHQ) led to 52.5% additional patients (557 of 1088) with complete outcome assessment (estimated ‘retention benefit’) compared with in-person FALCON trial follow-up alone. No adverse events were reported related to completion of in-person FALCON trial follow-up or the telephone WHQ.

There were several differences in the patients who had in-person FALCON trial follow-up, and telephone FALCON trial follow-up only and no trial follow-up (*Table S5*). Of note, there were fewer patients in rural settings (30.7% *versus* 39.0%; *P* =0.006) and fewer male patients (48.0% *versus* 58.7%; *P* < 0.001), as well as more patients with an obstetric indication (18.8% *versus* 5.9%; *P* < 0.001) and urogenital (22.0% *versus* 8.1%; *P* < 0.001), clean/clean-contaminated (36.7% *versus* 22.8%; *P* < 0.001), and open non-midline (44.6% *versus* 24.1%; *P* < 0.001) surgery that returned for in-person *versus* telephone FALCON follow-up. However, patients from all participating countries and having a mixture of baseline risk and operation types were included in both groups. Of the patients who had in-person FALCON trial follow-up (531 patients), the timing of the telephone WHQ was per protocol for 388 patients (73.1%) and outside of protocol for 141 patients (26.6%) (*Fig. S3*).

### Diagnostic accuracy

The level of data missingness overall for all item responses was low (13 of 10 089; 0.1%) and similarly for each individual item (range 0.0–0.1%), so complete case analysis was conducted without imputation. Patients’ total WHQ scores for those with and without a diagnosis of SSI made at the FALCON trial assessment 30 days after surgery are presented in *Fig. S4* and *Table S6*. The proportion of patients with an SSI at each WHQ point score interval is presented in *Fig. S5.* In patients with a WHQ point score of zero (that is they did not report any symptoms of SSI over the telephone; 147 patients) who did go on to have an SSI diagnosis made on 30-day follow-up (7 patients), the features that were most commonly detected in person were purulent fluid (6 of 7 patients), pain at the wound site (6 of 7 patients), and diagnosis of SSI by a clinician or on imaging (6 of 7 patients) (*Table S7*).

**Fig. 3:**
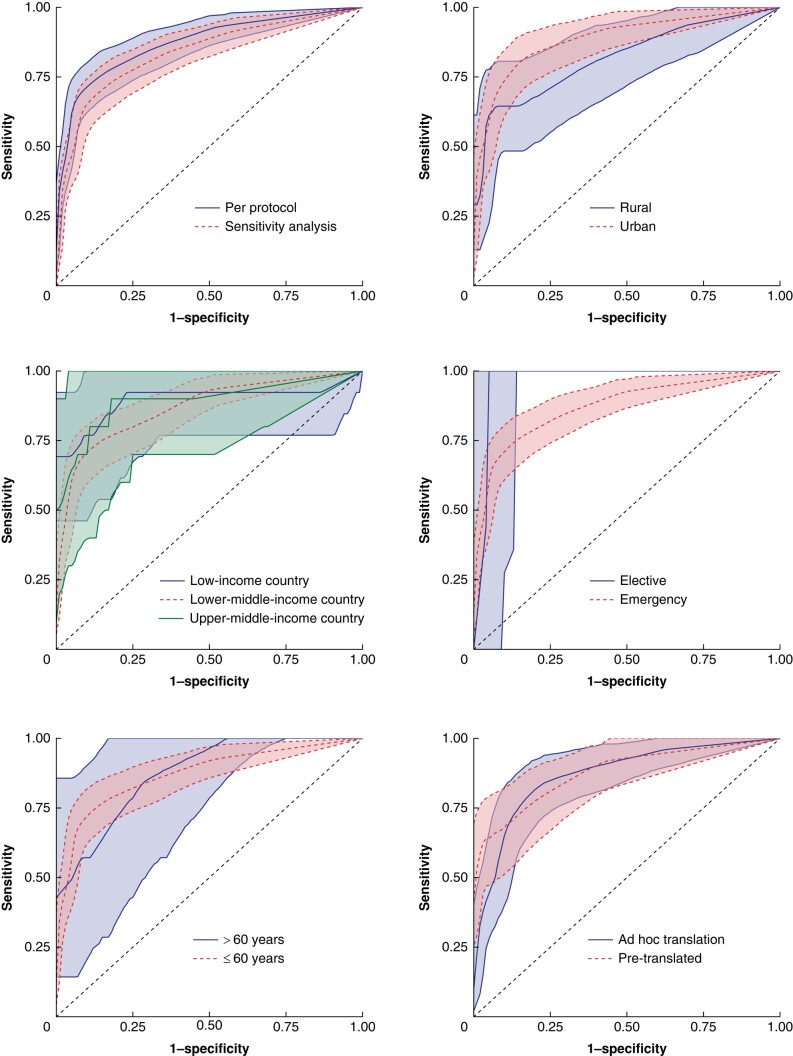
Receiver operating characteristic curves for the Wound Healing Questionnaire in detecting surgical-site infection up to 30 days after surgery SSI, surgical-site infection.

A summary of the performance metrics and diagnostic test accuracy statistics is shown in *Table 3*, *Table S8*, and *Fig. 3*. In the per-protocol analysis (388 patients), the WHQ demonstrated excellent overall discrimination (AUROC 0.869 (95% c.i. 0.824 to 0.914)). The discrimination was similar in sensitivity analyses including out-of-protocol patients (531 patients; AUROC 0.836 (95% c.i. 0.788 to 0.883)), including post-discharge SSI only (300 patients; AUROC 0.863 (95% c.i. 0.790 to 0.937)), and with inverse probability weighting (AUROC 0.866 (95% c.i. 0.805 to 0.927)). Diagnostic test accuracy statistics at different cut-off points ‘to rule in’ or ‘rule out’ SSI are presented in *Table S9*. The cut-off point identified using Youden’s index was 3.5 (WHQ total score greater than or equal to 4), which diagnosed post-discharge SSI with a sensitivity of 0.701 (95% c.i. 0.610 to 0.792), a specificity of 0.911 (95% c.i. 0.878 to 0.9430), a positive predictive value of 0.723 (95% c.i. 0.633 to 0.814), and a negative predictive value of 0.901 (95% c.i. 0.867 to 0.935).

**Table 3:** Diagnostic test accuracy characteristics overall and across subgroups

Patient group	Number of patients	SSI prevalence‡	AUROC (95% c.i.)	Accuracy (95% c.i.)	Sensitivity (95% c.i.)	Specificity (95% c.i.)	WHQ score cut-off§
Overall*	388	97 (25.0)	0.869 (0.824,0.914)	0.858 (0.858,0.859)	0.701 (0.610,0.792)	0.911 (0.878,0.9430	3.5
**Sensitivity analyses**							
Including out-of-protocol patients	531	104 (19.6)	0.836 (0.788,0.883)	0.842 (0.841,0.842)	0.673 (0.583,0.763)	0.883 (0.852,0.913)	3.5
No discharge SSI	300	32 (10.1)	0.863 (0.790,0.937)	0.923 (0.923,0.924)	0.625 (0.457,0.793)	0.959 (0.935,0.983)	4.5
**Subgroup analyses**							
Patient home location							
Urban	266	66 (24.8)	0.886 (0.836,0.937)	0.838 (0.837,0.839)	0.818 (0.725,0.911)	0.845 (0.795,0.895)	2.5
Rural	122	31 (25.4)	0.818 (0.721,0.914)	0.877 (0.875,0.879)	0.613 (0.441,0.784)	0.967 (0.930,1.004)	4.5
Country income							
Upper-middle	39	10 (25.6)	0.888 (0.741,1.000)	0.846 (0.840,0.853)	0.900 (0.714,1.086)	0.828 (0.690,0.965)	2.5
Lower-middle	314	74 (23.6)	0.868 (0.817,0.918)	0.866 (0.866,0.867)	0.689 (0.584,0.795)	0.921 (0.887,0.955)	3.5
Low	35	13 (37.1)	0.892 (0.748,1.000)	0.829 (0.837,0.837)	0.923 (0.778,1.068)	0.773 (0.598,0.948)	1.5
Patient age (years)							
>60	43	7 (16.3)	0.867 (0.729,1.000)	0.721 (0.712,0.730)	0.857 (0.589,1.116)	0.694 (0.544,0.845)	1.5
≤60	345	90 (26.1)	0.869 (0.821,0.916)	0.852 (0.851,0.853)	0.722 (0.630,0.815)	0.898 (0.861,0.935)	3.5
Urgency of surgery							
Emergency	364	95 (26.1)	0.871 (0.826,0.916)	0.830 (0.829,0.830)	0.758 (0.672,0.844)	0.855 (0.813,0.897)	2.5
Elective	21	2 (8.3)	0.966 (0.895,1.000)	0.958 (0.955,0.962)	1.000 (1.000,1.000)	0.955 (0.868,1.042)	4.5
Questionnaire translation							
Formal translation	184	36 (19.6)	0.875 (0.803,0.946)	0.913 (0.912,0.914)	0.611 (0.452,0.770)	0.986 (0.968,1.005)	4.5
Ad hoc translation†	178	50 (28.1)	0.866 (0.805,0.927)	0.798 (0.796,0.796)	0.840 (0.738,0.942)	0.781 (0.710,0.853)	2.5
Severity of SSI							
No re-operation (mild SSI only)	360	73 (20.3)	0.855 (0.801,0.908)	0.822 (0.821,0.823)	0.726 (0.624,0.828)	0.847 (0.805,0.888)	2.5

Values are *n* (%) unless otherwise indicated. Full set of test accuracy statistics presented in *Table S8*. *Overall analysis included only patients with per-protocol WHQ administration. †Includes ad hoc, translated from English by questionnaire administrator and ad hoc, translated from English with formal translator. ‡Surgical-site infection recorded using reference test of 30-day in-person FALCON trial follow-up. §Cut-off scores defined using Youden’s index, which maximizes the sum of sensitivity and specificity in the cohort of interest. Implementation of the WHQ should be supported by clinical decision-making using cut-off scores in *Table S9*. SSI, surgical-site infection; AUROC, area under the receiver operating characteristic curve (used as an overall measure of discrimination); WHQ, Wound Healing Questionnaire.

The performance of the WHQ was maintained across key subgroups (*Fig. 3*). Some differences were observed (reduced overall discrimination in rural settings (AUROC 0.818 (95% c.i. 0.721 to 0.914)) *versus* urban settings (AUROC 0.886 (95% c.i. 0.836 to 0.937)) and poorer discrimination after emergency surgery (AUROC 0.871 (95% c.i. 0.826 to 0.916)) *versus* elective surgery (AUROC 0.966 (95% c.i. 0.895 to 1.000))), although the 95% confidence intervals overlapped for both of these comparisons and interpretation of the analysis of elective surgery was limited by a low SSI rate in the elective surgery subgroup.

### Community engagement and involvement

Patients had a direct impact on study delivery and reporting. First, variables related to acceptability, the number of attempts needed, and time taken were added to the telephone WHQ pathway item set in response to pilot testing and early exploration of the data during study monitoring. Second, several suggestions were provided to iteratively improve the implementation of telephone WHQ administration. To summarize this shared learning, a toolkit (*Appendix I*) was co-produced with study authors and provided to sites to share best practice for acceptable and inclusive delivery of a telephone follow-up pathway. This was presented as a slide presentation (Microsoft Powerpoint^®^, Microsoft Corporation, Redmond, WA, USA) and infographic poster (Adobe Illustrator^®^, Adobe, San Jose, CA, USA). Finally, subgroup analysis was added for mild SSI only, due to concerns that patients with less severe problems may be missed and so delay receiving care.

## Discussion

This prospective validation study within a large international pragmatic trial demonstrated high feasibility and validity of telephone assessment for diagnosis of SSI in low-resource environments using the adapted WHQ. The WHQ was demonstrated to be suitable for use across a diverse range of settings, countries, and languages in three continents with high completion and low missing data rates. The diagnostic accuracy of the WHQ score was good when delivered per-protocol and was robust regarding several sensitivity analyses. However, it was less discriminative in certain subgroups, such as patients living in rural areas. Several cut-off points of the WHQ score and their corresponding diagnostic accuracy statistics were presented to facilitate application of the WHQ to different contexts. Co-production of the telephone WHQ administration pathway facilitated culturally and contextually attuned delivery. This tool is now available for global implementation in postoperative surveillance pathways and to optimize efficient trial design and conduct.

Few existing high-quality studies have evaluated the diagnostic accuracy of telemedicine methods for remote diagnosis of SSI. A prospective cohort study published in 2022 raised a significant concern for under-detection of SSI using non-standardized methods. On meta-analysis, only four studies were identified with paired in-person and telephone follow-up for which diagnostic test accuracy statistics could be calculated^13^. Three studies were at high risk of bias and just one, the UK validation of the English language WHQ, was identified as being at low risk of bias^23^. This is why this instrument was chosen to update and adapt to use in this international study. These international data therefore play an important role in informing safe upscaling of methods for remote postoperative surveillance. Differences in performance for patients living in rural *versus* urban settings may reflect differences in items related to the treatment pathway for wound infection (for example seeking advice for a wound problem, readmission to hospital) and patients’ access to care in rural environments. Whilst the sensitivity may be marginally reduced, remote follow-up methods may improve the reach into these communities, improve diversity and representation, and reduce attrition bias.

There are several ways in which this WHQ instrument may be applied. First, it may be used in research studies to provide a diagnosis of SSI (that is binary outcome of SSI/no SSI) remotely, without the need for in-person review. Choice of cut-off SSI threshold score would need to consider a balance of sensitivity and specificity, and the consequences of missing or over diagnosing SSI. This has important implications for trial design and conduct. Trials regarding SSI need to be large and pragmatic, and a validated remote method for assessing SSI will reduce trial costs. Second, the WHQ may be used in clinical practice to triage patients into existing clinical care pathways, with those at very low risk of a SSI diagnosis being given reassurance and those at moderate or high risk of a SSI diagnosis being asked to return for outpatient assessment. This could be adopted by either primary or secondary care depending on the structure of the local health system, but will require further optimization during implementation. Other work in this area has suggested that triage using remote, digital methods is safe and feasible, and has cost savings^35^. Combining remote tools to detect SSI and other common postoperative complications could be an accessible and rapid step towards the digital future of surgery. However, further research is needed to explore the impact of remote assessment pathways on reducing delays to care and improving clinical outcomes.

The present study confirms that digital follow-up pathways in low-resource environments are feasible and resilient. This supports estimates of high access to mobile communications by the World Bank^36^. By moving to remote, telephone assessment, over 50% additional patients were able to be followed up who may otherwise have been lost to follow-up, substantially improving trial retention^29,37–39^. Intuitively, the time from surgery to attempted follow-up was strongly associated with the likelihood of successful contact. Certain groups were highlighted to be more challenging to reach. Patients with non-midline incisions may represent patients undergoing appendicectomy or cholecystectomy, who are likely to return to work soon after their operation and have limited physical opportunity and/or reflective motivation to complete follow-up^40^. An association between SSI diagnosis and reduced odds of successful contact highlights a potential risk for attrition bias. Specific efforts to improve retention in these groups should be co-developed by CEI partners in future trials.

Postoperative surveillance is burdensome in high-volume, low-resource settings, both for patients and health systems. Remote follow-up is likely to substantially reduce direct and indirect costs (for example time out of work, informal caregivers), for patients who may already be at risk of catastrophic expenditure as a result of their surgical episode^10^. Task shifting of wound assessment to more junior or non-clinical staff is likely to significantly improve efficiency and reduce the ‘footprint’ of research studies on local systems. Here, the WHQ was largely delivered by non-expert assessors, which both reduced the opportunity cost to the limited surgical workforce and built capacity in research skills and wound evaluation.

Video and photographic assessment of the healing surgical wound is a promising area of innovation that was not evaluated in the present study^41,42^. Assessment using the telephone WHQ was less accurate for ‘mild SSI’ (that is not needing reoperation) in a subgroup analysis and signs such as purulent fluid, wound opening, and greater than expected pain on palpation were sometimes missed by the WHQ in an exploratory analysis. The evidence base for adoption of this ‘enhanced’ remote assessment remains scarce, but it has been widely adopted during the SARS-CoV-2 pandemic^43,44^. The data of the present study show promise for the feasibility of video and photographic assessment in low-resource contexts, with 64.2% of patients having access to a mobile telephone with a camera (range by country: 11.1% in Rwanda to 80.6% in Mexico). Urgent evidence is required to better understand the safety and potential limitations of this practice.

The present study has several limitations. One, it is assumed that the reference test of in-person assessment could correctly detect when a wound infection had or had not occurred. Whilst in the FALCON trial there was a minimum training requirement for those involved in wound evaluation, false positives or false negatives at in-person review would affect the estimates of diagnostic accuracy upon administration of the WHQ and no fully objective reference test is available. Two, there was a theoretical risk of patients developing a new SSI between their WHQ completion and 30-day follow-up. This is clinically unlikely and not supported by existing cohort data^29^. Three, the WHQ was commonly performed with ad hoc translation by the questionnaire administrator. This may have decreased both the reproducibility and accuracy of the instrument, but reflected the diverse, real-world setting of delivery, and no significant difference was seen in discrimination when translation was performed ad hoc *versus* with a pre-translated questionnaire. Four, despite a careful quality assurance and training process, repeated measurements were not performed, so inter-rater or intra-rater reliability could not be evaluated. Five, the study was underpowered to explore differences in accuracy between countries or languages. Six, there was a risk of partial verification bias in only including patients with in-person FALCON follow-up in the diagnostic accuracy analysis, but this was addressed using inverse probability weighting. Seven, small changes to the published SAP were made; however, these were responsive to CEI and investigators’ priorities and are described transparently. Eight, participants were concurrently involved in an RCT in SSI reduction so may have been more conscious of symptoms of infection than a general population. Nine, acceptability of telephone follow-up was assessed at the end of the telephone WHQ and was not anonymized, so was at risk of social acceptability bias. It is also possible that patients with more SSI symptoms had more frequent healthcare interactions and were able to respond more accurately to the WHQ creating a verification bias. The study excluded patients who died before 30 days (29 patients), representing a competing risk when interpreting the generalizability of the results to a highest-risk group of patients. All three of these sources of bias had minimal impact on relative diagnostic ORs upon meta-epidemiological analysis. Finally, we did not assess accuracy of the WHQ in detecting SSI in non-abdominal surgery or the clinical impact of introducing pathways for remote wound surveillance into routine practice; this highlights important areas for further research.

However, with a robust protocol, run through the platform of a randomized trial and in accordance with best-practice guidelines, it provides high-quality evidence to support implementation of postoperative tele-surveillance. TALON also provides a proof of concept for international SWATs, which can now be used to explore other high-priority methodological challenges in other global health trials, including outcome assessment in other perioperative events.

## Collaborators

**NIHR Global Health Research Unit on Global Surgery**

James Glasbey; Adesoji Ademuyiwa; Alisha Bhatt; Bruce Biccard; Jane Blazeby; Peter Brocklehurst; Sohini Chakrabortee; JC Allen Ingabire; Francis Moïse Dossou; Irani Durán; Rohini Dutta; Dhruva Ghosh; Frank Gyamfi; Parvez Haque; Pollyanna Hardy; Mike Horton; Gabriella Hyman; Ritu Jain; Oluwaseun Ladipo-Ajayi; Ismail Lawani; Souliath Lawani; Mwayi Kachapila; Rachel Lillywhite; Rhiannon Macefield; Laura Magill; Janet Martin; Jonathan Mathers; Kenneth McLean; Punam Mistry; Rohin Mittal; Mark Monahan; Rachel Moore; Dion Morton; Moyo Ojo; Faustin Ntirenganya; Emmanuel Ofori; Rupert Pearse; Alberto Peón; Thomas Pinkney; Antonio Ramos de la Medina; Tubasiime Ronald; David Roman; Emmy Runingamugabo; Alice Sitch; Anita Slade; Stephen Tabiri; Donna Smith; Aneel Bhangu; James Glasbey; Alice Sitch; Anita Slade; Duc Khanh To; Aneel Bhangu; Pollyanna Hardy; Adesoji O Ademuyiwa; Lawani Ismail; Dhruva Ghosh; Antonio Ramos de la Medina; Rachel Moore; Faustin Ntirenganya; Stephen Tabiri; Emmy Runingamugabo; Simin Patrawala; Angela Prah; Christian Oko; Karolin Kroese; Ismaïl Lawani; Francis Moïse Dossou; Corinne Dzemta; Covalic Melic Bokossa Kandokponou; Souliath Lawani; Hulrich Behanzin; Cyrile Kpangon; Bernard Appiah Ofori; Stephen Tabiri; Abdul-Hafiz Saba; Gbana Limann; Daniel Kwesi Acquah; Shamudeen Mohammed Alhassan; Sheriff Mohammed; Owusu Abem Emmanuel; Yakubu Musah; Yenli Edwin; Sheba Kunfah; Yakubu Mustapha; Abantanga Atindaana Francis; Emmanuel Ayingayure; Gbana Limann; Forster Amponsah-Manu; Eric Agyemang; Vera Agyekum; Esther Adjei-Acquah; Emmanuel Yaw Twerefour; Barbra Koomson; Ruby Acheampong Boateng; Ato Oppong Acquah; Richard Ofosu-Akromah; Leslie Issa Adam-Zakariah; Nii Armah Adu-Aryee; Theodore Wordui; Coomson Christian Larbi; Akosa Appiah Enoch; Mensah Elijah; Kyeremeh Christian; Addo Gyambibi Kwame; Boakye Percy; Kontor Effah Bismark; Gyamfi Brian; Manu Ruth; Romeo Hussey; Samuel Dadzie; Akosua Dwamena Appiah; Grace Yeboah; Cynthia Yeboah; James Amoako; Regina Acquah; Naa Anyekaa Sowah; Atta Kusiwaa; Esther Asabre; Cletus Ballu; Charles Gyamfi Barimah; Frank Owusu; Clement Sie-Broni; Vivian Adobea; Prince Yeboah Owusu; Marshall Zume; Abdul-Hamid Labaran; Raphael Adu-Brobbey; Martin Tangnaa Morna; Samuel A. Debrah; Patrick Opoku Manu Maison; Michael Nortey; Donald Enti; Mabel Pokuah Amoako-Boateng; Anthony Baffour Appiah; Emmanuel Owusu Ofori; Richard Kpankpari; Benedict Boakye; Elizabert Mercy Quartson; Patience Koggoh; Anita Eseenam Agbeko; Frank Enoch Gyamfi; Joshua Arthur; Joseph Yorke; Christian Kofi Gyasi-Sarpong; Charles Dally; Agbenya Kobla Lovi; Michael Amoah; Boateng Nimako; Robert Sagoe; Anthony Davor; Fareeda Galley; Michael Adinku; Jonathan Boakye-Yiadom; Jane Acquaye; Juliana Appiah; Dorcas Otuo Acheampong; Iddrisu Haruna; Edward Amoah Boateng; Emmanuel Kafui Ayodeji; Samuel Tuffuor; Naa Kwarley; Yaa Tufuor; Ramatu Darling Abdulai; Fred Dankwah; Ralph Armah; Doris Ofosuhene; Dorcas Osei-Poku; Arkorful Ebenezer Temitope; Delali Akosua Gakpetor; Victoria Sena Gawu; Christopher Asare; Enoch Tackie; James Ankomah; Isaac Omane Nyarko; Zelda Robertson; Serbeh Godwin; Appiah Anthony Boakye; Godfred Fosu; Frank Assah-Adjei; Parvez Haque; Ritu Jain; Alisha Bhatt; Jyoti Dhiman; Rohini Dutta; Dhruva Ghosh; Esther Daniel; Priyadarshini K; Latha Madankumar; Rohin Mittal; Ida Nagomy; Soosan Prasad; Arpit Jacob Mathew; Danita Prakash; Priya Jacob; Jeremiah P Anachy; Amy Mathew; Josy Thomas; Philip V Alexander; Pradeep Zechariah; Neerav D Aruldas; Asif Mehraj; Hafsa Imtiyaz Ahmed; Rauf A Wani; Fazl Q Parray; Nisar A Chowdri; Antonio Ramos De la Medina; Laura Martinez Perez Maldonado; Diana S Gonzalez Vazquez; Iran I Durán Sánchez; Maria J Martínez Lara; Alejandra Nayen Sainz de la Fuente; Ana O Cortes Flores; Mariana E Barreto Gallo; Alejandro Gonzalez Ojeda; Monica E Jimenez Velasco; Luis Hernández Miguelena; Reyes J Cervantes Ortiz; Gonzalo I Hernandez Gonzalez; Marco Hurtado Romero; Rosa I Hernandez Krauss; Luis A Dominguez Sansores; Alejandro Cuevas Avendaño; Celina Cuellar Aguirre; Isaac Baltazar Gomez; Hector Ortiz Mejia; Alejandro González Ojeda; Oscar E Olvera Flores; Erick A González García de Rojas; Kevin J Pintor Belmontes; Francisco J Barbosa Camacho; Aldo Bernal Hernández; Laura Reyes Aguirre; Rubén E Morán Galaviz; Clotilde Fuentes Orozco; Wenceslao G Ángeles Bueno; Fernando S Ramirez Marbello; Diego E Luna Acevedo; Michel Hernández Valadez; Ana L Bogurin Arellano; Luis R Ramírez-González; Bertha G Guzmán Ramírez; Eduardo Valtierra Robles; Ramona I Rojas García; José V Pérez Navarro; Edgar J Cortes Torres; David R Dominguez Solano; Alberto N Peón; Roque D Lincona Menindez; Rozana Reyes Gamez; Maria C Paz Muñoz; Orimisan Belie; Victoria Adeleye; Adesoji Ademuyiwa; Oluwafunmilayo Adeniyi; Opeyemi Akinajo; David Akinboyewa; Felix Alakaloko; Oluwole Atoyebi; Olanrewaju Balogun; Christopher Bode; Olumide Elebute; Francis Ezenwankwo; Adesiyakan Adedotun; George Ihediwa; Jubril Kuku; Oluwaseun Ladipo-Ajayi; Ayomide Makanjuola; Samuel Nwokocha; Olubunmi Ogein; Rufus Ojewola; Abraham Oladimeji; Thomas Olajide; Iyabo Alasi; Oluwaseun Oluseye; Justina Seyi-Olajide; Adaiah Soibi-Harry; Emmanuel Williams; Agbulu Moses Vincent; Nnamdi Jonathan Duru; Kenneth Uche Onyekachi; Christiana Ashley; Chinelo Victoria Mgbemena; Moyosoluwa Ojo; Olowu Oluyemisi; Iyabode Ikuewunmi; Adeoluwa Adebunmi; Edet Glory Bassey; Ephraim Okwudiri Ohazurike; Olayide Michael Amao; Osunwusi Benedetto Oluwaseun; Emily Doris; Olutola Stephen; Christianah Gbenga-Oke; Olawunmi Olayioye; Olowu Oluyemisi; Kayode Oluremi; Esther Abunimye; Christianah Oyegbola; Olayade Kayode; Adeola Ayoola Orowale; Omolara M Williams; Olufunmilade A Omisanjo; Omolara M Faboya; Zainab O Imam; Olabode A Oshodi; Yusuf A Oshodi; Ayokunle A Ogunyemi; Olalekan T Ajai; Francisca C Nwaenyi; Adewale O Adisa; Adewale A Aderounmu; Funmilola O Wuraola; Oludayo Sowande; Lukman Olajide Abdur-Rahman; Jibril Oyekunle Bello; Hadijat Olaide Raji; Nurudeen Abiola Adeleke; Saheed Abolade Lawal; Rafiat Tinuola Afolabi; Abdulwahab Lawal; Okechukwu Hyginus Ekwunife; Ochomma Amobi Egwuonwu; Chisom Faith Uche; Abubakar Bala AB Muhammad; Saminu S Muhammad; Idris Usman IU Takai; Mohammed AS Aliyu Salele; Onyekachi G Ukata; Mahmoud Kawu MK Magashi; Lawal Barau LB Abdullahi; Bello Abodunde BA Muideen; Khadija A Ado; Lofty-John Chukwuemeka LJC Anyawu; Samson Olori; Samuel A Sani; Olabisi O Osagie; Ndubuisi Mbajiekwe; Oseremen Aisuodionoe-Shadrach; Godwin O Akaba; Lazarus Ameh; Lazarus Ameh; Francis o Adebayo; Martins Uanikhoba; Felix O Ogbo; Nancy O Tabuanu; Taiwo A Lawal; Rukiyat A Abdus-Salam; Akinlabi E Ajao; Augustine O Takure; Omobolaji O Ayandipo; Hyginus O Ekwuazi; Olukayode Abayomi; Olatunji O Lawal; Solomon Olagunju; Kelvin I Egbuchulem; Sikiru Adekola Adebayo; Peter Elemile; Usang E Usang; Joseph E Udosen; Expo E Edet; Akan W Inyang; Edima M Olory; Gabriel U Udie; Godwin O Chiejina; Adams D Marwa; Faith J Iseh; Sunday A Ogbeche; Mary O Isa; Uchechukwu O Ezomike; Sebastian O Ekenze; Matthew I Eze; Emmanuel O Izuka; Jude K Ede; Vincent C Enemuo; Okezie M Mbadiwe; Ngozi G Mbah; Alphonsine Imanishimwe; Sosthene Habumuremyi; Faustin Ntirenganya; JC Allen Ingabire; Isaie Ncogoza; Emmanuel Munyaneza; Jean de Dieu Haragirimana; Christian Jean Urimubabo; Violette Mukanyange; Jeannette Nyirahabimana; Emmanuel Mutabazi; Christophe Mpirimbanyi; Olivier Mwenedata; Hope Lydia Maniraguha; Emelyne Izabiriza; Moses Dusabe; Job Zirikana; Francine Uwizeyimana; Josiane Mutuyimana; Elisee Rwagahirima; Alphonsine Imanishimwe; Ronald Tubasiime; Aphrodis Munyaneza; Sosthene Habumuremyi; Salathiel Kanyarukiko; Gibert Ndegamiye; Francine Mukaneza; Jean Claude Uwimana; Pierrine Nyirangeri; Deborah Mukantibaziyaremye; Aime Dieudonne Hirwa; Salomee Mbonimpaye; Piolette Muroruhirwe; Christine Mukakomite; Elysee Kabanda; Rachel Moore; Ncamsile Anthea Nhlabathi; Maria Fourtounas; Mary Augusta Adams; Gabriella Hyman; Hlengiwe Samkelisiwe Nxumalo; Nnosa Sentholang; Mmule Evelyn Sethoana; Mpho Nosipho Mathe; Zain Ally; Margot Flint; Bruce Biccard; Adesoji O Ademuyiwa; Adewale O. Adisa; Aneel Bhangu; Peter Brocklehurst; Sohini Chakrabortee; Pollyanna Hardy; Ewen Harrison; JC Allen Ingabire; Parvez D Haque; Lawani Ismail; James Glasbey; Dhruva Ghosh; Frank Enoch Gyamfi; Elizabeth Li; Rachel Lillywhite; Antonio Ramos de la Medina; Rachel Moore; Laura Magill; Dion Morton; Dmitri Nepogodiev; Faustin Ntirenganya; Thomas Pinkney; Omar Omar; Joana Simoes; Donna Smith; Stephen Tabiri; Adesoji O Ademuyiwa; Lawani Ismail; Dhruva Ghosh; Antonio Ramos de la Medina; Rachel Moore; Faustin Ntirenganya; Stephen Tabiri; Adesoji Ademuyiwa; Aneel Bhangu; Felicity Brant; Peter Brocklehurst; Sohini Chakrabortee; Dhruva Ghosh; James Glasbey; Pollyanna Hardy; Ewen Harrison; Emily Heritage; Lawani Ismail; Karolin Kroese; Carmela Lapitan; Rachel Lillywhite; David Lissauer; Laura Magill; Antonio Ramos de la Medina; Punam Mistry; Mark Monahan; Rachel Moore; Dion Morton; Dmitri Nepogodiev; Faustin Ntirenganya; Omar Omar; Thomas Pinkney; Tracy Roberts; Donna Smith; Stephen Tabiri; Neil Winkles; Pollyanna Hardy; Omar Omar; Emmy Runigamugabo; Azmina Verjee; Pierre Sodonougbo; Pamphile Assouto; Michel Fiogbe; Houenoukpo Koco; Serge Metchinhoungbe; Hodonou Sogbo; Hulrich Behanzin; Djifid Morel Seto; Yannick Tandje; Sosthène Kangni; Cyrile Kpangon; Marcelin Akpla; Hugues Herve Chobli; Blaise Kovohouande; Gérard Agboton; Rene Ahossi; Raoul Baderha Ngabo; Nathan Bisimwa; Covalic Melic Bokossa Kandokponou; Mireille Dokponou; Francis Moïse Dossou; Corinne Dzemta; Antoine Gaou; Roland Goudou; Emmanuel Hedefoun; Sunday Houtoukpe; Felix Kamga; Eric Kiki-Migan; Souliath Lawani; Ismaïl Lawani; René Loko; Afissatou Moutaïrou; Pencome Ogouyemi; Fouad Soumanou; Pia Tamadaho; Mack-Arthur Zounon; Luke Aniakwo Adagrah; Bin Baaba Alhaji Alhassan; Mabel Pokuah Amoako-Boateng; Anthony Baffour Appiah; Alvin Asante-Asamani; Benedict Boakye; Samuel A Debrah. Donald Enti; Rahman Adebisi Ganiyu; Patience Koggoh; Richard Kpankpari; Isabella Naa M. Opandoh; Meshach Agyemang Manu; Maison Patrick Opoku Manu; Samuel Mensah; Martin Tangnaa Morna; John Nkrumah; Michael Nortey; Emmanuel Owusu Ofori; Elizaberth Mercy Quartson; Esther Adjei-Acquah; Vera Agyekum; Eric Agyemang; Rebecca Adjeibah Akesseh; Forster Amponsah-Manu; Richard Ofosu-Akromah; Ato Oppong Acquah; Leslie Issa Adam-Zakariah; Esther Asabre; Ruby Acheampong Boateng; Barbara Koomson; Ataa Kusiwaa; Emmanuel Yaw Twerefour; James Ankomah; Frank Assah-Adjei; Anthony Appiah Boakye; Godfred Fosu; Godwin Serbeh; Kofi Yeboah Gyan; Isaac Omane Nyarko; Zelda Robertson; Ralph Armah; Christopher Asare; Delali Akosua Gakpetor; Victoria Sena Gawu; Ambe Obbeng; Doris Ofosuhene; Dorcas Osei-Poku; Diana Puozaa; Enoch Tackie; Arkorful Ebenezer Temitope; Regina Acquah; James Amoako; Akosua Dwamena Appiah; Mark Aseti; Charles Banka; Samuel Dadzie; Derick Essien; Frank Enoch Gyamfi; Romeo Hussey; Jemima Kwarteng; Naa Anyekaa Sowah; Grace Yeboah; Cynthia Yeboah; Kwame Gyambibi Addo; Enoch Appiah Akosa; Percy Boakye; Christian Larbi Coompson; Brian Gyamfi; Bismark Effah Kontor; Christian Kyeremeh; Ruth Manu; Elijah Mensah; Friko Ibrahim Solae; Gideon Kwasi Toffah; Dorcas Otuo Acheampong; Jane Acquaye; Michael Adinku; Kwabena Agbedinu; Anita Eseenam Agbeko; Emmanuel Gyimah Amankwa; Michael Amoah; George Amoah; Juliana Appiah; Joshua Arthur; Alex Ayim; Emmanuel Kafui Ayodeji; Jonathan Boakye-Yiadom; Edward Amoah Boateng; Charles Dally; Anthony Davor; Christian Kofi Gyasi-Sarpong; Naabo Nuhu Noel Hamidu; Iddrisu Haruna; Naa Kwarley; Agbenya Kobla Lovi; Boateng Nimako; Bertina Beauty Nyadu; Dominic Opoku; Anita Osabutey; Robert Sagoe; Samuel Tuffour; Yaa Tufour; Francis Akwaw Yamoah; Abiboye Cheduko Yefieye; Joseph Yorke; Nii Armah Adu-Aryee; Faisal Adjei; Erica Akoto; Elikem Ametefe; Joachim Kwaku Amoako; Godsway Solomon Attepor; George Darko Brown; Benjamin Fenu; Philemon Kwame Kumassah; David Olatayo Olayiwola; Theodore Wordui; Nelson Agboadoh; Fatao Abubakari; Cletus Ballu; Charles Gyamfi Barimah; Guy Casskey Boateng; Prosper Tonwisi Luri; Abraham Titigah; Frank Owusu; Raphael Adu-Brobbey; Christian Larbi Coompson; Abdul-Hamid Labaran; Junior Atta Owusu; Vivian Adobea; Amos Bennin; Fred Dankwah; Stanley Doe; Ruth Sarfo Kantanka; Ephraim Kobby; Kennedy Kofi Korankye Hanson Larnyor; Edwin Osei; Prince Yeboah Owusu; Clement Ayum Sie-Broni; Marshall Zume; Francis Atindaana Abantanga; Darling Ramatu Abdulai; Daniel Kwesi Acquah; Emmanuel Ayingayure; Imoro Osman; Sheba Kunfah; Gbana Limann; Shamudeen Alhassan Mohammed; Sheriff Mohammed; Yakubu Musah; Bernard Ofori; Emmanuel Abem Owusu; Abdul-Hafiz Saba; Anwar Sadat Seidu; Stephen Tabiri; Mustapha Yakubu; Edwin Mwintiereh Taang Yenli; Arun Gautham; Alice Hepzibah; Grace Mary; Deepak Singh; Dimple Bhatti; William Bhatti; Karan Bir; Swati Daniel; Tapasya Dhar; Jyoti Dhiman; Dhruva Ghosh; Sunita Goyal; Ankush; Goyal; Monika Hans; Parvez Haque; Samuel Konda; Anil Luther; Amit Mahajan; Shalini Makkar; Kavita Mandrelle; Vishal Michael; Partho Mukherjee; Reuben Rajappa; Prashant Singh; Atul Suroy; Ravinder Thind; Alen Thomas; Arti Tuli; Sreejith Veetil; Esther Daniel Mark Jesudason; Priyadarshini K; Latha Madankumar; Rohin Mittal; Ida Nagomy; Rajesh Selvakumar; Bharat Shankar; Moonish Sivakumar; Rajeevan Sridhar; Cecil Thomas; Devabalan Titus; Manisha Aggarwal; Parth Dhamija; Himani Gupta; Vinoth Kanna; Ashwani Kumar; Gurtaj Singh; Philip Alexander; Josy Thomas; Pradeep Zechariah; Amos Dasari; Priya Jacob; Elizabeth Kurien; Arpit Mathew; Danita Prakash; Anju Susan; Rose Varghese; Rahul Alpheus; Ashish Choudhrie; Hemanth Kumar; Nitin Peters; Subrat Raul; Rajeev Sharma; Rakesh Vakil; Wenceslao Ángeles Bueno; Francisco Barbosa Camacho; Aldo Bernal Hernández; Ana Bogurin Arellano; Edgar Cortes Torres; Clotilde Fuentes Orozco; Erick González García de Rojas; Alejandro González Ojeda; Bertha Guzmán Ramírez; Michel Hernández Valadez; Diego Luna Acevedo; Rubén Morán Galaviz; Oscar Olvera Flores; José Pérez Navarro; Kevin Pintor Belmontes; Fernando Ramirez Marbello; Luis Ramírez-González; Laura Reyes Aguirre; Ramona Rojas García; Eduardo Valtierra Robles; Reyes Cervantes Ortiz; Gonzalo Hernandez Gonzalez; Rosa Hernandez Krauss; Luis Hernández Miguelena; Marco Hurtado Romero; Isaac Baltazar Gomez; Celina Cuellar Aguirre; Alejandro Cuevas Avendaño; Luis Dominguez Sansores; Hector Ortiz Mejia; Laura Urdapilleta Gomez del Campo; Claudia Caballero Cerdan; David Dominguez Solano; Rafael Toriz Garcia; Mariana Barreto Gallo; Ana Cortes Flores; Alejandro Gonzalez Ojeda; Monica Jimenez Velasco; Rozana Reyes Gamez; Roque Lincona Menindez; Alberto Navarrete Peón; Maria Paz Muñoz; Irán Irani Durán Sánchez; Diana Samantha González Vázquez; María José Martínez Lara; Laura Martinez Perez Maldonado; Alejandra Nayen Sainz de la Fuente; Antonio Ramos De la Medina; Lawal Abdullahi; Khadija Ado; Mohammed Aliyu; Lofty-John Anyanwu; Mahmoud Magashi; Abubakar Muhammad; Saminu Muhammad; Bello Muideen; Idris Takai; Onyekachi Ukata; Opeoluwa Adesanya; David Awonuga; Olushola Fasiku; Chidiebere Ogo; Moruf Abdulsalam; Abimbola Adeniran; Olalekan Ajai; Olukemi Akande; Kazeem Atobatele; Grace Eke; Omolara Faboya; Zainab Imam; Esther Momson; Francisca Nwaenyi; Ayokunle Ogunyemi; Mobolaji Oludara; Olufunmilade Omisanjo; Olabode Oshodi; Yusuf Oshodi; Yemisi Oyewole; Omotade Salami; Omolara Williams; Victoria Adeleye; Adesoji Ademuyiwa; Oluwafunmilayo Adeniyi; Opeyemi Akinajo; David Akinboyewa; Iyabo Alasi; Felix Alakaloko; Oluwole Atoyebi; Olanrewaju Balogun; Orimisan Belie; Christopher Bode; Andrew Ekwesianya; Olumide Elebute; Francis Ezenwankwo; Adedeji Fatuga; George Ihediwa; Adesola Jimoh; Jubril Kuku; Oluwaseun LadipoAjayi; Ayomide Makanjuola; Olayanju Mokwenyei; Samuel Nwokocha; Olubunmi Ogein; Rufus Ojewola; Abraham Oladimeji; Thomas Olajide; Oluwaseun Oluseye; Justina Seyi-Olajide; Adaiah Soibi-Harry; Aloy Ugwu; Emmanuel Williams; Ochomma Egwuonwu; Okechukwu Ekwunife; Victor Modekwe; Chukwuemeka Okoro; Chisom Uche; Kenneth Ugwuanyi; Chuka Ugwunne; Akeem Adeleke; Wilson Adenikinju; Olumide Adeniyi; Akinfolarin Adepiti; Adewale Aderounmu; Abdulhafiz Adesunkanmi; Adewale Adisa; Samuel Ajekwu; Olusegun Ajenjfuja; Jerrie Akindojutimi; Akinbolaji Akinkuolie; Olusegun Alatise; Olubukola Allen; Lukmon Amosu; Micheal Archibong; Olukayode Arowolo; Deborah Ayantona; Ademola Ayinde; Olusegun Badejoko; Tajudeen Badmus; Amarachukwu Etonyeaku; Emeka Igbodike; Omotade Ijarotimi; Adedayo Lawal; Fayowole Nana; Tunde Oduanafolabi; Olalekan Olasehinde; Olaniyi Olayemi; Stephen Omitinde; Owolabi Oni; Chigozie Onyeze; Ernest Orji; Adewale Rotimi; Abdulkadir Salako; Olufemi Solaja; Oluwaseun Sowemimo; Ademola Talabi; Mohammed Tajudeen; Funmilola Wuraola; Francis Adebayo; Oseremen Aisuodionoe-Shadrach; Godwin Akaba; Lazarus Ameh; Ndubuisi Mbajiekwe; Felix Ogbo; Samson Olori; Olabisi Osagie; Abu Sadiq; Samuel Sani; Nancy Tabuanu; Martins Uanikhoba; Godwin Chiejina; Ekpo Edet; Akan Inyang; Mary Isa; Faith Iseh; Adams Marwa; Sunday Ogbeche; Edima Olory; Gabriel Udie; Joseph Udosen; Usang Usang; Olukayode Abayomi; Rukiyat Abdus-Salam; Sikiru Adebayo; Akinlabi Ajao; Olanrewaju Amusat; Omobolaji Ayandipo; Kelvin Egbuchulem; Hyginus Ekwuazi; Peter Elemile; Taiwo Lawal; Olatunji Lawal; Solomon Olagunju; Peter Osuala; Bamidele Suleman; Augustine Takure; Lukman Abdur-Rahman; Nurudeen Adeleke; Muideen Adesola; Rafiat Afolabi; Sulaiman Agodirin; Isiaka Aremu; Jibril Bello; Saheed Lawal; Abdulwahab Lawal; Hadijat Raji; Olayinka Sayomi; Asimiyu Shittu; Jude Ede; Sebastian Ekenze; Vincent Enemuo; Matthew Eze; Uchechukwu Ezomike; Emmanuel Izuka; Okezie Mbadiwe; Ngozi Mbah; Uba Ezinne; Matthew Francis; Iweha Ikechukwu; Okoi Nnyonno; Philemon Okoro; Igwe Patrick; John Raphael; Oriji Vaduneme; Abhulimen Victor; Salathiel Kanyarukiko; Francine Mukaneza; Deborah Mukantibaziyaremye; Aphrodis Munyaneza; Gibert Ndegamiye; Ronald Tubasiime; Moses Dusabe; Emelyne Izabiriza; Hope Lydia Maniraguha; Christophe Mpirimbanyi; Josiane Mutuyimana; Olivier Mwenedata; Elisee Rwagahirima; Francine Uwizeyimana; Job Zirikana; Aime Dieudonne Hirwa; Elysee Kabanda; Salomee Mbonimpaye; Christine Mukakomite; Piolette Muroruhirwe; Georges Bucyibaruta; Gisele Juru Bunogerane; Sosthene Habumuremyi; Jean de Dieu Haragirimana; Alphonsine Imanishimwe; JC Allen Ingabire; Violette Mukanyange; Emmanuel Munyaneza; Emmanuel Mutabazi; Isaie Ncogoza; Faustin Ntirenganya; Jeannette Nyirahabimana; Christian Urimubabo; Mary Augusta Adams; Richard Crawford; Chikwendu Jeffrey Ede; Maria Fourtounas; Gabriella Hyman; Zafar Khan; Morapedi Kwati; Mpho Nosipho Mathe; Rachel Moore; Ncamsile Anthea Nhlabathi; Hlengiwe Samkelisiwe Nxumalo; Paddy Pattinson; Nnosa Sentholang; Mmule Evelyn Sethoana; Maria Elizabeth Stassen; Laura Thornley; Paul Wondoh; Cheryl Birtles; Mathete Ivy; Cynthia Mbavhalelo; Zain Ally; Abdus-sami Adewunmi; Jonathan Cook; David Jayne; Soren Laurberg; Julia Brown; Simon Cousens; Neil Smart.

## Supplementary Material

znad446_Supplementary_Data

## Data Availability

Anonymized data are available upon request to the TALON Study Management Group and successful completion of a Data Sharing Agreement.
