# Erratum to: Accuracy of the Wound Healing Questionnaire in the diagnosis of surgical-site infection after abdominal surgery in low- and middle-income countries

**DOI:** 10.1093/bjs/znae069

**Published:** 2024-03-09

This is an erratum to: NIHR Global Health Research Unit on Global Surgery. Accuracy of the Wound Healing Questionnaire in the diagnosis of surgical-site infection after abdominal surgery in low- and middle-income countries. *British Journal of Surgery*, Volume 111, Issue 2, February 2024, znad446, https://doi.org/10.1093/bjs/znad446

In the originally published version of this article, the abstract was omitted due to an error that occurred during the production process.

The Publisher apologizes for this.

The article has been corrected.

