# Supplementary material for: Accuracy of the Wound Healing Questionnaire in the diagnosis of surgical-site infection after abdominal surgery in low- and middle-income countries
Source: Br J Surg. 2024 Feb 26;111(2):znad446. doi: 10.1093/bjs/znad446 (PMC10895408; doi:10.1093/bjs/znad446)
Supplement: znad446_Supplementary_Data [file znad446_supplementary_data.zip › TALON appendix V5.0 28.05.23.docx]

**Supplementary materials: Accuracy of the Wound Healing Questionnaire in diagnosis of surgical site infection after abdominal surgery in low and middle-income countries**

NIHR Global Health Research Unit on Global Surgery

Correspondence to:

Mr James Glasbey MBBCh BSC PGCert MRCS, NIHR Doctoral Research Fellow in Global Surgery, NIHR Global Health Research Unit on Global Surgery, University of Birmingham, Institute of Translational Medicine, Mindelsohn Way, Birmingham, B15 2TH, Email: [j.glasbey@bham.ac.uk](mailto:j.glasbey@bham.ac.uk)

Table of contents

[Appendix A. Collaborating authors (PubMed citable)](#_Toc139052849)

[Appendix B. Full definition of reference test](#_Toc139052850)

[Appendix C. Summary of adaptation of Wound Healing Questionnaire](#_Toc139052851)

[Appendix D. Adapted Wound Healing Questionnaire used in TALON study](#_Toc139052852)

[Appendix E. Monitoring and quality assurance of WHQ delivery](#_Toc139052853)

[Appendix F. Methodological adaptation to the SARS-CoV-2 pandemic](#_Toc139052854)

[Appendix G. Protocol sample size considerations](#_Toc139052855)

[Appendix H. Changes to published Statistical Analysis Plan](#_Toc139052856)

[Appendix I. Co-produced toolkit for optimising postoperative telephone follow-up](#_Toc139052857)

[Supplementary table 1. Baseline demographics by country (N=1196)](#_Toc139052858)

[Supplementary table 2. Comparison of patients included in TALON study within a trial and FALCON trial overall 30](#_Toc139052859)

[Supplementary table 3. Feasibility outcomes by country (N=1196) 31](#_Toc139052860)

[Supplementary table 4. Factors associated with successful telephone contact in a multivariable model (Figure 2). 32](#_Toc139052861)

[Supplementary table 5. Differences in baseline demographics between patients contactable by telephone that had in-person FALCON trial follow-up and telephone FALCON trial follow-up (N=1088). 33](#_Toc139052862)

[Supplementary table 6. Cross-tabulation of patients WHQ score and whether or not they received a diagnosis of SSI at the in-person assessment 30-days after surgery 34](#_Toc139052863)

[Supplementary table 7. Misclassification in patients with a WHQ score of zero (N=147) 35](#_Toc139052864)

[Supplementary table 8. Full summary of diagnostic test accuracy characteristics overall and across subgroups 36](#_Toc139052865)

[Supplementary table 9. Diagnostic accuracy of the WHQ score across different cut-points to ‘rule in’ or ‘rule out’ SSI 37](#_Toc139052866)

[Supplementary figure 1. Proportion of patients with successful telephone contact with increasing time from surgery (days) 38](#_Toc139052867)

[Supplementary figure 2. Proportion of patients with in-person FALCON trial versus telephone only follow-up (N=1088) 39](#_Toc139052868)

[Supplementary figure 3. Timing of telephone WHQ administration](#_Toc139052869)

[Supplementary figure 4 (Panel). Wound Healing Questionnaire score versus reference test diagnosis of SSI (N=388)](#_Toc139052870)

[Supplementary figure 5. Proportion of patients with SSI diagnosis in reference test at each WHQ point score level (per-protocol analysis)](#_Toc139052871)

Appendix A. Collaborating authors (PubMed citable)

***Writing group***

James Glasbey (UK), Adesoji Ademuyiwa (Nigeria), Alisha Bhatt (India), Bruce Biccard (South Africa), Jane Blazeby (UK), Peter Brocklehurst (UK), Sohini Chakrabortee (UK), JC Allen Ingabire (Rwanda), Francis Moïse Dossou (Benin), Irani Durán (Mexico), Rohini Dutta (India), Dhruva Ghosh (India), Frank Gyamfi (Ghana), Parvez Haque (India), Pollyanna Hardy (UK), Mike Horton (UK), Gabriella Hyman (South Africa), Ritu Jain (India), Oluwaseun Ladipo-Ajayi (Nigeria), Ismail Lawani (Benin), Souliath Lawani (Benin), Mwayi Kachapila (UK), Rachel Lillywhite (UK), Rhiannon Macefield (UK), Laura Magill (UK), Janet Martin (Canada), Jonathan Mathers (UK), Kenneth McLean (UK), Punam Mistry (UK), Rohin Mittal (India), Mark Monahan (UK), Rachel Moore (South Africa), Dion Morton (UK), Moyo Ojo (Nigeria), Faustin Ntirenganya (Rwanda), Emmanuel Ofori (Ghana), Rupert Pearse (UK), Alberto Peón (Mexico), Thomas Pinkney (UK), Antonio Ramos de la Medina (Mexico), Tubasiime Ronald (Rwanda), David Roman (Mexico), Emmy Runingamugabo (Rwanda), Alice Sitch (UK), Anita Slade (UK), Stephen Tabiri (Ghana), Donna Smith (UK), Aneel Bhangu (UK).

***Statistical analysis***

James Glasbey, Alice Sitch, Anita Slade, Duc Khanh To, Aneel Bhangu, Pollyanna Hardy

***National hub lead investigators***

Adesoji O Ademuyiwa, Lawani Ismail, Dhruva Ghosh, Antonio Ramos de la Medina, Rachel Moore, Faustin Ntirenganya, Stephen Tabiri

***Community engagement and involvement partners***

Emmy Runingamugabo (Rwanda), Simin Patrawala (India), Angela Prah (Ghana), Christian Oko (Nigeria), Karolin Kroese (UK)

**TALON collaborators**

***Benin***

Ismaïl Lawani, Francis Moïse Dossou*, Corinne Dzemta, Covalic Melic Bokossa Kandokponou, Souliath LAWANI (Centre Hospitalier Universitaire et Départemental Ouémé-Plateau (CHUDOP), Porto-Novo)

Hulrich Behanzin* (Hopital de Zone de Menontin, Contonou)

Cyrile Kpangon* (Centre Hospitalier Universitaire de Zone de Suru Lere (SLERE), Contonou)

***Ghana***

Bernard Appiah Ofori, Stephen Tabiri*, Abdul-Hafiz Saba, Gbana Limann, Daniel Kwesi Acquah, Shamudeen Mohammed Alhassan, Sheriff Mohammed, Owusu Abem Emmanuel, Yakubu Musah, Yenli Edwin, Sheba Kunfah, Yakubu Mustapha, Abantanga Atindaana Francis, Emmanuel Ayingayure, Gbana Limann (Tamale Teaching Hospital, Tamale)

Forster Amponsah-Manu, Eric Agyemang, Vera Agyekum, Esther Adjei-Acquah, Emmanuel Yaw Twerefour, Barbra Koomson, Ruby Acheampong Boateng, Ato Oppong Acquah, Richard Ofosu-Akromah, Leslie Issa Adam-Zakariah (Eastern Regional Hospital, Koforidua)

Nii Armah Adu-Aryee, Theodore Wordui (Korle-Bu Teaching Hospital, Accra)

Coomson Christian Larbi, Akosa Appiah Enoch, Mensah Elijah, Kyeremeh Christian, Addo Gyambibi Kwame, Boakye Percy, Kontor Effah Bismark, Gyamfi Brian, Manu Ruth (Techiman Holy Family Hospital (THFH), Techniman)

Romeo Hussey, Samuel Dadzie, Akosua Dwamena Appiah, Grace Yeboah, Cynthia Yeboah, James Amoako, Regina Acquah, Naa Anyekaa Sowah (Berekum Holy Family Hospital (BHFH), Berekum)

Atta Kusiwaa, Esther Asabre (Effia-Nkwanta Regional Hospital (ENRH), Takoradi)

Cletus Ballu, Charles Gyamfi Barimah (Salaga District Hospital, Salaga)

Frank Owusu (St. Patrick's Hospital, Offinso)

Clement Sie-Broni, Vivian Adobea, Prince Yeboah Owusu, Marshall Zume, Abdul-Hamid Labaran, Raphael Adu-Brobbey (Sunyani Regional Hospital (SRH), Sunyan)

Martin Tangnaa Morna, Samuel A. Debrah, Patrick Opoku Manu Maison, Michael Nortey, Donald Enti, Mabel Pokuah Amoako-Boateng, Anthony Baffour Appiah, Emmanuel Owusu Ofori, Richard Kpankpari, Benedict Boakye, Elizabert Mercy Quartson, Patience Koggoh (Cape Coast Teaching Hospital, Cape Coast)

Anita Eseenam Agbeko, Frank Enoch Gyamfi, Joshua Arthur, Joseph Yorke, Christian Kofi Gyasi-Sarpong, Charles Dally, Agbenya Kobla Lovi, Michael Amoah, Boateng Nimako, Robert Sagoe, Anthony Davor, Fareeda Galley, Michael Adinku, Jonathan Boakye-Yiadom, Jane Acquaye, Juliana Appiah, Dorcas Otuo Acheampong, Iddrisu Haruna, Edward Amoah Boateng, Emmanuel Kafui Ayodeji, Samuel Tuffuor, Naa Kwarley, Yaa Tufuor (Komfo Anokye Teaching Hospital (KATH), Kumasi)

Ramatu Darling Abdulai, Fred Dankwah, Ralph Armah, Doris Ofosuhene, Dorcas Osei-Poku, Arkorful Ebenezer Temitope, Delali Akosua Gakpetor, Victoria Sena Gawu, Christopher Asare, Enoch Tackie ((Greater) Accra Regional Hospital (ARH), Accra)

James Ankomah, Isaac Omane Nyarko, Zelda Robertson, Serbeh Godwin, Appiah Anthony Boakye, Godfred Fosu, Frank Assah-Adjei (Goaso Municipal Hospital, Goaso)

***India***

Christian Medical College, Ludhiana: Parvez Haque*, Ritu Jain*, Alisha Bhatt, Jyoti Dhiman, Rohini Dutta, Dhruva Ghosh

Christian Medical College, Vellore: Esther Daniel, Priyadarshini K, Latha Madankumar, Rohin Mittal*, Ida Nagomy, Soosan Prasad

Madhipura Christian Hospital, Madhipura: Arpit Jacob Mathew*, Danita Prakash, Priya Jacob, Jeremiah P Anachy, Amy Mathew

Lady Willingdon Hospital, Manali: Josy Thomas, Philip V Alexander*, Pradeep Zechariah, Neerav D Aruldas

Sher-i-Kashmir Institute Of Medical Sciences, Srinagar: Asif Mehraj*,Hafsa Imtiyaz Ahmed, Rauf A Wani, Fazl Q Parray, Nisar A Chowdri

***Mexico***

Antonio Ramos De la Medina*, Laura Martinez Perez Maldonado, Diana S Gonzalez Vazquez, Iran I Durán Sánchez, Maria J Martínez Lara, Alejandra Nayen Sainz de la Fuente (Hospital Español Veracruz, Veracruz)

Ana O Cortes Flores*, Mariana E Barreto Gallo, Alejandro Gonzalez Ojeda, Monica E Jimenez Velasco (Hospital San Javier, Guadalajara)

Luis Hernández Miguelena*, Reyes J Cervantes Ortiz, Gonzalo I Hernandez Gonzalez, Marco Hurtado Romero, Rosa I Hernandez Krauss (Hospital Regional, Veracruz)

Luis A Dominguez Sansores*, Alejandro Cuevas Avendaño, Celina Cuellar Aguirre, Isaac Baltazar Gomez, Hector Ortiz Mejia (Hospital General de Boca Del Rio, Boca del Rio)

Alejandro González Ojeda*, Oscar E Olvera Flores, Erick A González García de Rojas, Kevin J Pintor Belmontes, Francisco J Barbosa Camacho, Aldo Bernal Hernández, Laura Reyes Aguirre, Rubén E Morán Galaviz, Clotilde Fuentes Orozco, Wenceslao G Ángeles Bueno, Fernando S Ramirez Marbello, Diego E Luna Acevedo, Michel Hernández Valadez, Ana L Bogurin Arellano, Luis R Ramírez-González, Bertha G Guzmán Ramírez, Eduardo Valtierra Robles, Ramona I Rojas García, José V Pérez Navarro, Edgar J Cortes Torres (Hospital De Especialidades, CMNO, Guadalajara)

David R Dominguez Solano* (Hospital Regional de Xalapa, Xalapa)

Alberto N Peón*, Roque D Lincona Menindez, Rozana Reyes Gamez, Maria C Paz Muñoz, (Hospital Espanol Pachuca, Puebla)

***Nigeria***

Lagos University Teaching Hospital, Lagos (Hub):Orimisan Belie, Victoria Adeleye, Adesoji Ademuyiwa*, Oluwafunmilayo Adeniyi, Opeyemi Akinajo, David Akinboyewa, Felix Alakaloko, Oluwole Atoyebi, Olanrewaju Balogun, Christopher Bode, Olumide Elebute, Francis Ezenwankwo, Adesiyakan Adedotun, George Ihediwa, Jubril Kuku, Oluwaseun Ladipo-Ajayi, Ayomide Makanjuola, Samuel Nwokocha, Olubunmi Ogein, Rufus Ojewola, Abraham Oladimeji, Thomas Olajide, Iyabo Alasi, Oluwaseun Oluseye, Justina Seyi-Olajide, Adaiah Soibi-Harry, Emmanuel Williams, Agbulu Moses Vincent, Nnamdi Jonathan Duru, Kenneth Uche Onyekachi, Christiana Ashley, Chinelo Victoria Mgbemena, Moyosoluwa Ojo, Olowu Oluyemisi, Iyabode Ikuewunmi, Adeoluwa Adebunmi, Edet Glory Bassey, Ephraim Okwudiri Ohazurike, Olayide Michael Amao, Osunwusi Benedetto Oluwaseun, Emily Doris, Olutola Stephen, Christianah Gbenga-Oke, Olawunmi Olayioye, Olowu Oluyemisi, Kayode Oluremi, Esther Abunimye, Christianah Oyegbola, Olayade Kayode, Adeola Ayoola Orowale,

Omolara M Williams*, Olufunmilade A Omisanjo, Omolara M Faboya, Zainab O Imam, Olabode A Oshodi, Yusuf A Oshodi, Ayokunle A Ogunyemi, Olalekan T Ajai, Francisca C Nwaenyi (Lagos State University Teaching Hospital (LASUTH))

Adewale O Adisa*, Adewale A Aderounmu, Funmilola O Wuraola, Oludayo Sowande (Obafemi Awolowo University Teaching Hospital, Ile-Ife (IFE))

Lukman Olajide Abdur-Rahman*, Jibril Oyekunle Bello, HADIJAT OLAIDE RAJI, Nurudeen Abiola Adeleke, Saheed Abolade Lawal, Rafiat Tinuola Afolabi, Abdulwahab Lawal (University of Ilorin Teaching Hospital (UITH), Ilorin)

Okechukwu Hyginus Ekwunife*, Ochomma Amobi Egwuonwu, Chisom Faith Uche (Nnamdi Azikiwe University Teaching Hospital (NAUTH), Nnewi)

Abubakar Bala AB Muhammad, Saminu S Muhammad, Idris Usman IU Takai, Mohammed AS Aliyu Salele, Onyekachi G Ukata, Mahmoud Kawu MK Magashi, Lawal Barau LB Abdullahi, Bello Abodunde BA Muideen, Khadija A Ado, Lofty-John Chukwuemeka LJC Anyawu* (Aminu Kano Teaching Hospital, Kano)

Samson Olori*, Samuel A Sani, Olabisi O Osagie, Ndubuisi Mbajiekwe, Oseremen Aisuodionoe-Shadrach, Godwin O Akaba, Lazarus Ameh, Lazarus Ameh, Francis o Adebayo, Martins Uanikhoba, Felix o Ogbo, Nancy O Tabuanu (University of Abuja Teaching Hospital, Abuja)

Taiwo A Lawal*, Rukiyat A Abdus-Salam, Akinlabi E Ajao, Augustine O Takure, Omobolaji O Ayandipo, Hyginus O Ekwuazi, Olukayode Abayomi, Olatunji O Lawal, Solomon Olagunju, Kelvin I Egbuchulem, Sikiru Adekola Adebayo, Peter Elemile (University College hospital, Ibadan)

Usang E Usang*, Joseph E Udosen, Expo E Edet, Akan W Inyang, Edima M Olory, Gabriel U Udie, Godwin O Chiejina, Adams D Marwa, Faith J Iseh, Sunday A Ogbeche, Mary O Isa (University of Calabar Teaching Hospital, Calabar)

Uchechukwu O Ezomike, Sebastian O Ekenze*, Matthew I Eze, Emmanuel O Izuka, Jude K Ede, Vincent C Enemuo, Okezie M Mbadiwe, Ngozi G Mbah (University of Nigeria Teaching Hospital (UNTH) Enugu)

***Rwanda***

Alphonsine Imanishimwe, Sosthene Habumuremyi, Faustin Ntirenganya*,JC Allen Ingabire, Isaie Ncogoza, Emmanuel Munyaneza, Jean de Dieu Haragirimana, Christian Jean Urimubabo, Violette Mukanyange, Jeannette Nyirahabimana, Emmanuel Mutabazi (Rwanda University Teaching Hospital of Kigali (CHUK), Kigali)

Christophe Mpirimbanyi*, Olivier Mwenedata, Hope Lydia Maniraguha, Emelyne Izabiriza, Moses Dusabe, Job Zirikana, Francine Uwizeyimana, Josiane Mutuyimana, Elisee Rwagahirima (Kibungo Referral Hospital (KIBUN), Ngoma District)

Alphonsine Imanishimwe, Ronald Tubasiime*, Aphrodis Munyaneza, Sosthene Habumuremyi, Salathiel Kanyarukiko, Gibert Ndegamiye, Francine Mukaneza, Jean Claude Uwimana, Pierrine Nyirangeri, Deborah Mukantibaziyaremye (Kibogora Hospital (KIBO), Kirambo)

Aime Dieudonne Hirwa*, Salomee Mbonimpaye, Piolette Muroruhirwe, Christine Mukakomite, Elysee Kabanda (Ruhengeri Referral Hospital, Musanze District)

***South Africa***

Rachel Moore, Ncamsile Anthea Nhlabathi, Maria Fourtounas, Mary Augusta Adams, Gabriella Hyman*, Hlengiwe Samkelisiwe Nxumalo, Nnosa Sentholang, Mmule Evelyn Sethoana, Mpho Nosipho Mathe (Chris Hani Baragwanath Hospital, Johannesburg)

Zain Ally* (Helen Joseph Hospital, Johannesburg)

Margot Flint, Bruce Biccard (Groote Schuur Hospital, Cape Town)

**FALCON Trial Investigators**

***FALCON writing group***

Adesoji O Ademuyiwa, Adewale O. Adisa, Aneel Bhangu, Peter Brocklehurst, Sohini Chakrabortee, Pollyanna Hardy, Ewen Harrison, JC Allen Ingabire, Parvez D Haque, Lawani Ismail, James Glasbey, Dhruva Ghosh, Frank Enoch Gyamfi, Elizabeth Li, Rachel Lillywhite, Antonio Ramos de la Medina, Rachel Moore, Laura Magill, Dion Morton, Dmitri Nepogodiev, Faustin Ntirenganya, Thomas Pinkney, Omar Omar, Joana Simoes, Donna Smith, Stephen Tabiri

***Hub leads***

Adesoji O Ademuyiwa, Lawani Ismail, Dhruva Ghosh, Antonio Ramos de la Medina, Rachel Moore, Faustin Ntirenganya, Stephen Tabiri

***Central Trial Management Group***

Adesoji Ademuyiwa, Aneel Bhangu, Felicity Brant, Peter Brocklehurst, Sohini Chakrabortee, Dhruva Ghosh, James Glasbey, Pollyanna Hardy, Ewen Harrison, Emily Heritage, Lawani Ismail, Karolin Kroese, Carmela Lapitan, Rachel Lillywhite, David Lissauer, Laura Magill, Antonio Ramos de la Medina, Punam Mistry, Mark Monahan, Rachel Moore, Dion Morton, Dmitri Nepogodiev, Faustin Ntirenganya, Omar Omar, Thomas Pinkney, Tracy Roberts, Donna Smith, Stephen Tabiri, Neil Winkles.

***Statistical analysis***

Pollyanna Hardy, Omar Omar

***Patient representatives***

Emmy Runigamugabo, Azmina Verjee

***FALCON collaborators*** *(alphabetical by country and surname; *denotes the hospital Principle Investigator):*

***Benin***

Clinique Universitaire d’Acceuil des Urgences - Centre National Hospitalier Universitaire Hubert Koutoucou MAGA, Cotonou: Pierre Sodonougbo, Pamphile Assouto*, Michel Fiogbe, Houenoukpo Koco, Serge Metchinhoungbe, Hodonou Sogbo

Hopital de Menontin, Cotonou: Hulrich Behanzin*, Djifid Morel Seto, Yannick Tandje Hopital de Zone de Suru Lere, Cotonou: Sosthène Kangni, Cyrile Kpangon*, Marcelin Akpla, Hugues Herve Chobli, Blaise Kovohouande

Centre Hospitalier Universitaire et Départemental de l’Ouémé-Plateau, Cotonou (Hub): Gérard Agboton, Rene Ahossi, Raoul Baderha Ngabo, Nathan Bisimwa, Covalic Melic Bokossa Kandokponou, Mireille Dokponou, Francis Moïse Dossou, Corinne Dzemta, Antoine Gaou, Roland Goudou, Emmanuel Hedefoun, Sunday Houtoukpe, Felix Kamga, Eric Kiki-Migan, Souliath Lawani, Ismaïl Lawani*, René Loko, Afissatou Moutaïrou, Pencome Ogouyemi, Fouad Soumanou, Pia Tamadaho, Mack-Arthur Zounon

***Ghana***

Cape Coast Teaching Hospital, Cape Coast: Luke Aniakwo Adagrah, Bin Baaba Alhaji Alhassan, Mabel Pokuah Amoako-Boateng, Anthony Baffour Appiah, Alvin Asante-Asamani, Benedict Boakye, Samuel A Debrah. Donald Enti, Rahman Adebisi Ganiyu, Patience Koggoh, Richard Kpankpari, Isabella Naa M. Opandoh, Meshach Agyemang Manu, Maison Patrick Opoku Manu, Samuel Mensah, Martin Tangnaa Morna*, John Nkrumah, Michael Nortey, Emmanuel Owusu Ofori, Elizaberth Mercy Quartson

Eastern Regional Hospital, Koforidua: Esther Adjei-Acquah, Vera Agyekum, Eric Agyemang, Rebecca Adjeibah Akesseh, Forster Amponsah-Manu*, Richard Ofosu-Akromah

Effia-Nkwanta Hospital, Sekondi-Takoradi: Ato Oppong Acquah, Leslie Issa Adam-Zakariah*, Esther Asabre, Ruby Acheampong Boateng, Barbara Koomson, Ataa Kusiwaa, Emmanuel Yaw Twerefour

Goaso Municipal Hospital, Goaso: James Ankomah*, Frank Assah-Adjei, Anthony Appiah Boakye, Godfred Fosu, Godwin Serbeh, Kofi Yeboah Gyan, Isaac Omane Nyarko, Zelda Robertson

Greater Accra Regional Hospital, Accra: Ralph Armah*, Christopher Asare, Delali Akosua Gakpetor, Victoria Sena Gawu, Ambe Obbeng, Doris Ofosuhene, Dorcas Osei-Poku, Diana Puozaa, Enoch Tackie, Arkorful Ebenezer Temitope

Holy Family Hospital, Berekum: Regina Acquah, James Amoako, Akosua Dwamena Appiah, Mark Aseti, Charles Banka, Samuel Dadzie, Derick Essien, Frank Enoch Gyamfi*, Romeo Hussey, Jemima Kwarteng, Naa Anyekaa Sowah, Grace Yeboah, Cynthia Yeboah

Holy Family Hospital, Techiman: Kwame Gyambibi Addo, Enoch Appiah Akosa, Percy Boakye, Christian Larbi Coompson*, Brian Gyamfi, Bismark Effah Kontor, Christian Kyeremeh, Ruth Manu, Elijah Mensah, Friko Ibrahim Solae, Gideon Kwasi Toffah

Komfo Anokye Teaching Hospital, Kumasi: Dorcas Otuo Acheampong, Jane Acquaye, Michael Adinku, Kwabena Agbedinu, Anita Eseenam Agbeko*, Emmanuel Gyimah Amankwa, Michael Amoah, George Amoah, Juliana Appiah, Joshua Arthur, Alex Ayim, Emmanuel Kafui Ayodeji, Jonathan Boakye-Yiadom, Edward Amoah Boateng, Charles Dally, Anthony Davor, Christian Kofi Gyasi-Sarpong, Naabo Nuhu Noel Hamidu, Iddrisu Haruna, Naa Kwarley, Agbenya Kobla Lovi, Boateng Nimako, Bertina Beauty Nyadu, Dominic Opoku, Anita Osabutey, Robert Sagoe, Samuel Tuffour, Yaa Tufour, Francis Akwaw Yamoah, Abiboye Cheduko Yefieye, Joseph Yorke

Korle Bu Teaching Hospital, Korle Bu: Nii Armah Adu-Aryee*, Faisal Adjei, Erica Akoto, Elikem Ametefe, Joachim Kwaku Amoako, Godsway Solomon Attepor, George Darko Brown, Benjamin Fenu, Philemon Kwame Kumassah, David Olatayo Olayiwola, Theodore Wordui, Nelson Agboadoh

Salaga District Hospital, Tamale: Fatao Abubakari, Cletus Ballu, Charles Gyamfi Barimah, Guy Casskey Boateng, Prosper Tonwisi Luri* Sandema District Hospital, Sandema: Abraham Titigah*

St. Patrick's Hospital, Offinso: Frank Owusu*

St Theresa’s Hospital, Nkoranza: Raphael Adu-Brobbey, Christian Larbi Coompson*, Abdul-Hamid Labaran, Junior Atta Owusu

Sunyani Regional Hospital, Sunyani: Vivian Adobea, Amos Bennin, Fred Dankwah*, Stanley Doe, Ruth Sarfo Kantanka, Ephraim Kobby, Kennedy Kofi Korankye Hanson Larnyor, Edwin Osei, Prince Yeboah Owusu, Clement Ayum Sie-Broni, Marshall Zume

Tamale Teaching Hospital, Tamale (Hub): Francis Atindaana Abantanga, Darling Ramatu Abdulai, Daniel Kwesi Acquah, Emmanuel Ayingayure, Imoro Osman, Sheba Kunfah, Gbana Limann, Shamudeen Alhassan Mohammed, Sheriff Mohammed, Yakubu Musah, Bernard Ofori, Emmanuel Abem Owusu, Abdul-Hafiz Saba, Anwar Sadat Seidu, Stephen Tabiri*, Mustapha Yakubu, Edwin Mwintiereh Taang Yenli

***India***

Chinchpada Christian Hospital, Chinchpada: Arun Gautham, Alice Hepzibah, Grace Mary, Deepak Singh

Christian Medical College, Ludhiana (Hub): Dimple Bhatti, William Bhatti, Karan Bir, Swati Daniel, Tapasya Dhar, Jyoti Dhiman, Dhruva Ghosh*, Sunita Goyal, Ankush, Goyal, Monika Hans, Parvez Haque*, Samuel Konda, Anil Luther, Amit Mahajan, Shalini Makkar, Kavita Mandrelle*, Vishal Michael, Partho Mukherjee, Reuben Rajappa, Prashant Singh, Atul Suroy, Ravinder Thind, Alen Thomas, Arti Tuli, Sreejith Veetil

Christian Medical College, Vellore: Esther Daniel Mark Jesudason, Priyadarshini K, Latha Madankumar, Rohin Mittal*, Ida Nagomy, Rajesh Selvakumar, Bharat Shankar, Moonish Sivakumar, Rajeevan Sridhar, Cecil Thomas, Devabalan Titus

Government Medical College, Patiala: Manisha Aggarwal, Parth Dhamija, Himani Gupta, Vinoth Kanna, Ashwani Kumar*, Gurtaj Singh Lady Willingdon Hospital, Manali: Philip Alexander*, Josy Thomas, Pradeep Zechariah

Madhipura Christian Hospital, Madhipura: Amos Dasari, Priya Jacob, Elizabeth Kurien, Arpit Mathew*, Danita Prakash, Anju Susan, Rose Varghese

Padhar Hospital, Betul: Rahul Alpheus, Ashish Choudhrie*

Post Graduate Institute Of Medical Education And Research, Chandigarh: Hemanth Kumar, Nitin Peters*

St. Stephens' Hospital, Delhi: Subrat Raul*, Rajeev Sharma, Rakesh Vakil

***Mexico***

Centro Medico Nacional de Occidente, Guadalajara: Wenceslao Ángeles Bueno, Francisco Barbosa Camacho, Aldo Bernal Hernández, Ana Bogurin Arellano, Edgar Cortes Torres, Clotilde Fuentes Orozco, Erick González García de Rojas, Alejandro González Ojeda*, Bertha Guzmán Ramírez, Michel Hernández Valadez, Diego Luna Acevedo, Rubén Morán Galaviz, Oscar Olvera Flores, José Pérez Navarro, Kevin Pintor Belmontes, Fernando Ramirez Marbello, Luis Ramírez-González, Laura Reyes Aguirre, Ramona Rojas García, Eduardo Valtierra Robles

Hospital de Alta Especialidad de Veracruz, Veracruz: Reyes Cervantes Ortiz, Gonzalo Hernandez Gonzalez, Rosa Hernandez Krauss, Luis Hernández Miguelena*, Marco Hurtado Romero

Hospital General de Boca del Rio, Boca del Rio: Isaac Baltazar Gomez, Celina Cuellar Aguirre, Alejandro Cuevas Avendaño, Luis Dominguez Sansores*, Hector Ortiz Mejia, Laura Urdapilleta Gomez del Campo

Hospital Regional de Xalapa, Xalapa: Claudia Caballero Cerdan, David Dominguez Solano*, Rafael Toriz Garcia

Hospital San Javier, Guadalajara: Mariana Barreto Gallo, Ana Cortes Flores*, Alejandro Gonzalez Ojeda, Monica Jimenez Velasco Sociedad Española de Beneficencia A.C, Pachuca: Rozana Reyes Gamez, Roque Lincona Menindez, Alberto Navarrete Peón*, Maria Paz Muñoz

Sociedad Española de Beneficencia, Veracruz (Hub): Irán Irani Durán Sánchez, Diana Samantha González Vázquez, María José Martínez Lara, Laura Martinez Perez Maldonado, Alejandra Nayen Sainz de la Fuente, Antonio Ramos De la Medina*

***Nigeria***

Aminu Kano Teaching Hospital, Kano: Lawal Abdullahi, Khadija Ado, Mohammed Aliyu, Lofty-John Anyanwu*, Mahmoud Magashi, Abubakar Muhammad, Saminu Muhammad, Bello Muideen, Idris Takai, Onyekachi Ukata

Federal Medical Centre, Abeokuta: Opeoluwa Adesanya*, David Awonuga, Olushola Fasiku, Chidiebere Ogo

Lagos State University Teaching Hospital, Lagos: Moruf Abdulsalam, Abimbola Adeniran, Olalekan Ajai, Olukemi Akande, Kazeem Atobatele, Grace Eke, Omolara Faboya, Zainab Imam, Esther Momson, Francisca Nwaenyi, Ayokunle Ogunyemi, Mobolaji Oludara, Olufunmilade Omisanjo, Olabode Oshodi, Yusuf Oshodi, Yemisi Oyewole, Omotade Salami, Omolara Williams*

Lagos University Teaching Hospital, Lagos (Hub): Victoria Adeleye, Adesoji Ademuyiwa*, Oluwafunmilayo Adeniyi, Opeyemi Akinajo, David Akinboyewa, Iyabo Alasi, Felix Alakaloko, Oluwole Atoyebi, Olanrewaju Balogun, Orimisan Belie, Christopher Bode, Andrew Ekwesianya, Olumide Elebute, Francis Ezenwankwo, Adedeji Fatuga, George Ihediwa, Adesola Jimoh, Jubril Kuku, Oluwaseun LadipoAjayi, Ayomide Makanjuola, Olayanju Mokwenyei, Samuel Nwokocha, Olubunmi Ogein, Rufus Ojewola, Abraham Oladimeji, Thomas Olajide, Oluwaseun Oluseye, Justina Seyi-Olajide, Adaiah Soibi-Harry, Aloy Ugwu, Emmanuel Williams

Nnamdi Azikwe University Teaching Hospital, Nnobi: Ochomma Egwuonwu, Okechukwu Ekwunife*, Victor Modekwe, Chukwuemeka Okoro, Chisom Uche, Kenneth Ugwuanyi, Chuka Ugwunne

Obafemi Awolowo University Teaching Hospitals, Ile-Ife: Akeem Adeleke, Wilson Adenikinju, Olumide Adeniyi, Akinfolarin Adepiti, Adewale Aderounmu, Abdulhafiz Adesunkanmi, Adewale Adisa*, Samuel Ajekwu, Olusegun Ajenjfuja, Jerrie Akindojutimi, Akinbolaji Akinkuolie, Olusegun Alatise, Olubukola Allen, Lukmon Amosu, Micheal Archibong, Olukayode Arowolo, Deborah Ayantona, Ademola Ayinde, Olusegun Badejoko, Tajudeen Badmus, Amarachukwu Etonyeaku, Emeka Igbodike, Omotade Ijarotimi, Adedayo Lawal, Fayowole Nana, Tunde Oduanafolabi, Olalekan Olasehinde, Olaniyi Olayemi, Stephen Omitinde, Owolabi Oni, Chigozie Onyeze, Ernest Orji, Adewale Rotimi, Abdulkadir Salako, Olufemi Solaja, Oluwaseun Sowemimo, Ademola Talabi, Mohammed Tajudeen, Funmilola Wuraola

University of Abuja Teaching Hospital, Abuja: Francis Adebayo, Oseremen Aisuodionoe-Shadrach, Godwin Akaba, Lazarus Ameh, Ndubuisi Mbajiekwe, Felix Ogbo, Samson Olori*, Olabisi Osagie, Abu Sadiq, Samuel Sani, Nancy Tabuanu, Martins Uanikhoba

University of Calabar, Teaching Hospital, Calabar: Godwin Chiejina, Ekpo Edet, Akan Inyang, Mary Isa, Faith Iseh, Adams Marwa, Sunday Ogbeche, Edima Olory, Gabriel Udie, Joseph Udosen, Usang Usang*

University College Hospital, Ibadan: Olukayode Abayomi, Rukiyat Abdus-Salam, Sikiru Adebayo, Akinlabi Ajao, Olanrewaju Amusat, Omobolaji Ayandipo, Kelvin Egbuchulem, Hyginus Ekwuazi, Peter Elemile, Taiwo Lawal*, Olatunji Lawal, Solomon Olagunju, Peter Osuala, Bamidele Suleman, Augustine Takure

University of Ilorin Teaching Hospital, Ilorin: Lukman Abdur-Rahman*, Nurudeen Adeleke, Muideen Adesola, Rafiat Afolabi, Sulaiman Agodirin, Isiaka Aremu, Jibril Bello, Saheed Lawal, Abdulwahab Lawal, Hadijat Raji, Olayinka Sayomi, Asimiyu Shittu

University of Nigeria Teaching Hospital, Enugu: Jude Ede, Sebastian Ekenze, Vincent Enemuo, Matthew Eze, Uchechukwu Ezomike, Emmanuel Izuka, Okezie Mbadiwe, Ngozi Mbah

University of Port Harcourt Teaching Hospital, Port Harcourt: Uba Ezinne, Matthew Francis, Iweha Ikechukwu, Okoi Nnyonno, Philemon Okoro*, Igwe Patrick, John Raphael, Oriji Vaduneme, Abhulimen Victor

***Rwanda***

Kibogora Hospital, Nyamasheke: Salathiel Kanyarukiko, Francine Mukaneza, Deborah Mukantibaziyaremye, Aphrodis Munyaneza, Gibert Ndegamiye, Ronald Tubasiime*

Kibungo Referral Hospital, Ngoma: Moses Dusabe, Emelyne Izabiriza, Hope Lydia Maniraguha, Christophe Mpirimbanyi*, Josiane Mutuyimana, Olivier Mwenedata, Elisee Rwagahirima, Francine Uwizeyimana, Job Zirikana

Ruhengeri Referral Hospital, Musanze: Aime Dieudonne Hirwa*, Elysee Kabanda, Salomee Mbonimpaye, Christine Mukakomite, Piolette Muroruhirwe

University Teaching Hospital Kigali, Kigali: Georges Bucyibaruta, Gisele Juru Bunogerane, Sosthene Habumuremyi, Jean de Dieu Haragirimana, Alphonsine Imanishimwe, JC Allen Ingabire, Violette Mukanyange, Emmanuel Munyaneza, Emmanuel Mutabazi, Isaie Ncogoza, Faustin Ntirenganya*, Jeannette Nyirahabimana, Christian Urimubabo

***South Africa***

Chris Hani Baragwanath Hospital, Johannesburg: Mary Augusta Adams, Richard Crawford, Chikwendu Jeffrey Ede, Maria Fourtounas, Gabriella Hyman, Zafar Khan, Morapedi Kwati, Mpho Nosipho Mathe, Rachel Moore*, Ncamsile Anthea Nhlabathi, Hlengiwe Samkelisiwe Nxumalo, Paddy Pattinson, Nnosa Sentholang, Mmule Evelyn Sethoana, Maria Elizabeth Stassen, Laura Thornley, Paul Wondoh Edenvale Hospital, Johannesburg: Cheryl Birtles, Mathete Ivy, Cynthia Mbavhalelo*

Helen Joseph Hospital, Johannesburg: Zain Ally* Sebokeng Hospital, Johannesburg: Abdus-sami Adewunmi*

***Independent Data Monitoring and Ethics Committee***

Jonathan Cook, David Jayne, Soren Laurberg

***Independent Trial Steering Committee (alphabetical)***

Julia Brown, Simon Cousens, Neil Smart

Appendix B. Full definition of reference test

The ‘gold standard’ reference diagnostic test for surgical site infection (SSI) during the 30-days after surgery for evaluation in this study is in-person review according to US Centres for Disease Control Criteria (3). The following definition was used in the host trial (FALCON) to identify deep incisional or superficial incisional SSIs:

- The infection must occur within 30-days of the index operation

AND

- The infection must involve the skin, subcutaneous, muscular, or fascial layers of the incision

AND

- The patient must have at least one of the following:
  - Purulent drainage from the wound
  - Organisms are detected from a wound swab
  - Wound opened spontaneously or by a clinician AND, at the surgical wound, the patient has at least one of: pain or tenderness; localised swelling; redness; heat; systemic fever (>38°C).
  - Diagnosis of SSI by a clinician or on imaging

The assessment will be made by an appropriately trained clinician (surgeon, trainee or nurse).

Appendix C. Summary of adaptation of Wound Healing Questionnaire

| **Item  number** | **Original item** | **Original response categories** | **Adapted item** | **Adapted response categories** |
| --- | --- | --- | --- | --- |
| 1 | Was there redness spreading away from the wound? | 1 = Not at all; 2 = A little; 3 = Quite a bit; 4 = A lot. | Was there redness (or shining of the skin) spreading away from the wound? | 1 = Not at all; 2 = A little; 3 = A lot. |
| 2 | Was the area around the wound warmer than the surrounding skin? | 1 = Not at all; 2 = A little; 3 = Quite a bit; 4 = A lot. | - | 1 = Not at all; 2 = A little; 3 = A lot. |
| 3 | Has any part of the wound leaked clear fluid? | 1 = Not at all; 2 = A little; 3 = Quite a bit; 4 = A lot. | Has any part of the wound leaked thin clear fluid? | 1 = Not at all; 2 = A little; 3 = A lot. |
| 4 | Has any part of the wound leaked blood-stained fluid? | 1 = Not at all; 2 = A little; 3 = Quite a bit; 4 = A lot. | - | 1 = Not at all; 2 = A little; 3 = A lot. |
| 5 | Has any part of the wound leaked thick and yellow or green fluid? | 1 = Not at all; 2 = A little; 3 = Quite a bit; 4 = A lot. | - | 1 = Not at all; 2 = A little; 3 = A lot. |
| 6 | Have the edges of any part of the wound separated or gaped open of their accord? | 1 = Not at all; 2 = A little; 3 = Quite a bit; 4 = A lot. | - | 1 = Not at all; 2 = A little; 3 = A lot. |
| 7 | If the wound edges opened, did the deeper tissue also separate? | 1 = Not at all; 2 = A little; 3 = Quite a bit; 4 = A lot. | If the wound edges opened, did the flesh beneath the skin or the inside sutures also separate? | 1 = Not at all; 2 = A little; 3 = A lot. |
| 8 | Has the area around the wound become swollen? | 1 = Not at all; 2 = A little; 3 = Quite a bit; 4 = A lot. | - | 1 = Not at all; 2 = A little; 3 = A lot. |
| 9 | Has the wound been smelly? | 1 = Not at all; 2 = A little; 3 = Quite a bit; 4 = A lot. | - | 1 = Not at all; 2 = A little; 3 = A lot. |
| 10 | Has the wound been painful to touch? | 1 = Not at all; 2 = A little; 3 = Quite a bit; 4 = A lot. | Has the wound been painful to touch? | 1 = Not at all; 2 = A little; 3 = A lot. |
| 11 | Have you had, or felt like you have had, a raised temperature or fever (>38oC)? | 1 = Not at all; 2 = A little; 3 = Quite a bit; 4 = A lot. | Have you had, or felt like you have had, a raised temperature or fever? | 1 = No; 2 = Yes |
| 12 | Have you sought advice because of a problem with your wound, other than at a planned follow-up appointment? | 1 = No; 2 = Yes | - | - |
| 13 | Has anything been put on the skin to cover the wound? (dressing) | 1 = No; 2 = Yes | - | - |
| 14 | Have you been back into hospital for a problem with your wound? | 1 = No; 2 = Yes | - | - |
| 15 | Have you been given antibiotics for a problem with your wound? | 1 = No; 2 = Yes | Have you been given medicines (antibiotics) for a problem with your wound? | - |
| 16 | Have the edges of your wound been deliberately separated by a doctor or nurse? | 1 = No; 2 = Yes | Have the edges of your wound been separated by a doctor or nurse? | - |
| 17 | Has your wound been scraped or cut to remove any unwanted tissue? | 1 = No; 2 = Yes | - | - |
| 18 | Has your wound been drained? (drainage of pus or an abscess) | 1 = No; 2 = Yes | Has thick, yellow, or green fluid (pus) been drained from your wound by a doctor or nurse? | - |
| 19 | Have you had an operation under general anaesthetic for treatment of a problem with your wound? | 1 = No; 2 = Yes | Have you had to go back to the operating room for treatment of a problem with your wound? | - |

Appendix D. Adapted Wound Healing Questionnaire used in TALON study

**Adapted Wound Healing Questionnaire**

**For questions with tick boxes, please tick one box per question.**

The Wound Healing Questionnaire should be completed between 27-30 days after the patient’s operation over the telephone, for patients that will undergo 30-day assessment as part of the FALCON trial. The Wound Healing Questionnaire should not be completed by the same person that will complete the standard 30-day FALCON Follow-up Form.

|  | | | FALCON Trial Number | | | | | | | | |  | | |  | | | | | | |  | | | |  | | |  | | | | |  | |
| --- | --- | --- | --- | --- | --- | --- | --- | --- | --- | --- | --- | --- | --- | --- | --- | --- | --- | --- | --- | --- | --- | --- | --- | --- | --- | --- | --- | --- | --- | --- | --- | --- | --- | --- | --- |
|  |  |  | Centre name | | | | | | | | | ____________________________ | | | | | | | | | | | | | | | | | | | | | | | |
|  |  |  | Patient name | | | | | | | | | ____________________________ | | | | | | | | | | | | | | | | | | | | | | | |
| **Patient status** | | | | | | | | | | | | | | | | | | | | | | | | | | | | | | | | | | | |
| Has the patient died? | | | | - Yes (***please stop at Patient Status***) | | | | | | | | | | | | | | | | | | - No (continue to Follow-up details) | | | | | | | | | | | | | |
| If patient died, date of death | | | | d | | | d | m | | m | y | | | y | | | y | | y | | | | | ***If patient died, an SAE form must be completed*** | | | | | | | | | | | |
| If patient died, main cause of death | | | | _________________________________________________ | | | | | | | | | | | | | | | | | | | | | | | | | | | | | | | |
| **Follow-up pathway** | | | | | | | | | | | | | | | | | | | | | | | | | | | | | | | | | | | |
| Attempts made to connect with patient | | | | - 1 attempt | | | | | | | | | | | | | | | | | | - 2-3 attempts | | | | | | | | | | | | | |
|  |  |  |  | - 4-5 attempts | | | | | | | | | | | | | | | | | | - >5 attempts | | | | | | | | | | | | | |
| Were you able to contact the patient by telephone? | | | | - Yes (please continue) | | | | | | | | | | | | | | | | | | - No (***please stop here***) | | | | | | | | | | | | | |
| If telephone contact was made, date of contact | | | | d | | | d | m | | m | y | | | y | | | y | | y | | | | |  | | | | | | | | | | | |
| *Ask the patient:*  What type of phone are you using for this call? | | | | - Landline phone | | | | | | | | | | | | | | | | - Mobile phone (without a camera) - Commercial call centre | | | | | | | | | | | | | | | |
|  |  |  |  | - Mobile phone (with a camera) | | | | | | | | | | | | | | | |  |  |  |  |  |  |  |  |  |  |  |  |  |  |  |  |
| *Ask the patient:*  Who owns the phone that you're speaking to me on? | | | | - Patient themselves | | | | | | | | | | | | | | | | - Friend or relative | | | | | | | | | | | | | | | |
|  |  |  |  | - Healthcare worker | | | | | | | | | | | | | | | | - Other (please specify):   _______________________ | | | | | | | | | | | | | | | |
| *Ask the patient:* Do you live in an urban (mostly city or town) or rural (mostly countryside) area? | | | | - Urban | | | | | | | | | | | | | | | | - Rural | | | | | | | | | | | | | | | |
| *Ask the patient:*  What is the highest level of education that you have achieved? | | | | - High/secondary school or above | | | | | | | | | | | | | | | | - Did not complete first/ primary school or no formal education | | | | | | | | | | | | | | | |
|  |  |  |  | - First/primary school level | | | | | | | | | | | | | | | |  |  |  |  |  |  |  |  |  |  |  |  |  |  |  |  |
| What language did the patient use to respond to the questionnaire? | | | | - English | | | | | | | | | | | | | | | | - Other (please specify):   _______________________ | | | | | | | | | | | | | | | |
| *If other*: Was the formal translated Questionnaire used? | | | | - Yes, formal questionnaire | | | | | | | | | | | | | | | | - No, translated by questionnaire administrator | | | | | | | | | | | | | | | |
|  |  |  |  | - No, using formal translator | | | | | | | | | | | | | | | |  |  |  |  |  |  |  |  |  |  |  |  |  |  |  |  |
| **Wound Healing Questionnaire** | | | | | | | | | | | | | | | | | | | | | | | | | | | | | | | | | | | |
| **Please read the following statement to the patient:**  We are interested in knowing how the cut(s) of your skin (called your wound(s)) have healed since you left hospital after your surgery. It is fine to ask someone else to help answer some of the questions, for example if you cannot easily see your wound(s). If you have more than one wound, please answer the questions thinking about just one wound. This should be either the wound with which you have had concerns about how it was healing, or the longest wound if there have been no specific concerns. We would like you to think about the wounds on your skin. Some of the questions I am about to ask you relate to some problems that may occur with wound healing. Please note, many people do not experience these problems after having surgery.  **Since you left hospital after having surgery…** | | | | | | | | | | | | | | | | | | | | | | | | | | | | | | | | | | | |
| Was there redness (or shining of the skin) spreading away from the wound? | | | | | | | | | - Not at all | | | | | | | | | | | | - A little | | | | | | | | | | - A lot | | | | |
| Was the area around the wound warmer than the surrounding skin? | | | | | | | | | - Not at all | | | | | | | | | | | | - A little | | | | | | | | | | - A lot | | | | |
| Has any part of the wound leaked thin, clear fluid? | | | | | | | | | - Not at all | | | | | | | | | | | | - A little | | | | | | | | | | - A lot | | | | |
| Has any part of the wound leaked blood-stained fluid? | | | | | | | | | - Not at all | | | | | | | | | | | | - A little | | | | | | | | | | - A lot | | | | |
| Has any part of the wound leaked thick and yellow or green fluid? | | | | | | | | | - Not at all | | | | | | | | | | | | - A little | | | | | | | | | | - A lot | | | | |
| Have the edges of any part of the wound separated or gaped open of their accord? | | | | | | | | | - Not at all | | | | | | | | | | | | - A little | | | | | | | | | | - A lot | | | | |
| *If the wound edges opened:* Did the flesh beneath the skin or the inside sutures also separate? | | | | | | | | | - Not at all | | | | | | | | | | | | - A little | | | | | | | | | | - A lot | | | | |
| Has the area around the wound become swollen? | | | | | | | | | - Not at all | | | | | | | | | | | | - A little | | | | | | | | | | - A lot | | | | |
| Has the wound been smelly? | | | | | | | | | - Not at all | | | | | | | | | | | | - A little | | | | | | | | | | - A lot | | | | |
| Has the wound been painful to touch? | | | | | | | | | - Not at all | | | | | | | | | | | | - A little | | | | | | | | | | - A lot | | | | |
| **Since you left hospital after having surgery…** | | | | | | | | | | | | | | | | | | | | | | | | | | | | | | | | | | | |
| Have you had, or felt like you have had, a raised temperature or fever? | | | | | | | | | | | | | | | | | | | | | | - Yes | | | | | | | | - No | | | | | |
| Have you sought advice because of a problem with your wound, other than at a planned follow-up appointment? | | | | | | | | | | | | | | | | | | | | | | - Yes | | | | | | | | - No | | | | | |
| Has anything been put on the skin to cover the wound? (dressing) | | | | | | | | | | | | | | | | | | | | | | - Yes | | | | | | | | - No | | | | | |
| Have you been back into hospital for a problem with your wound? | | | | | | | | | | | | | | | | | | | | | | - Yes | | | | | | | | - No | | | | | |
| Have you been given medicines (antibiotics) for a problem with your wound? | | | | | | | | | | | | | | | | | | | | | | - Yes | | | | | | | | - No | | | | | |
| Have the edges of your wound been separated by a doctor or nurse? | | | | | | | | | | | | | | | | | | | | | | - Yes | | | | | | | | - No | | | | | |
| Has your wound been scraped or cut to remove any unwanted flesh? | | | | | | | | | | | | | | | | | | | | | | - Yes | | | | | | | | - No | | | | | |
| Has thick, yellow or green fluid (pus) been drained from your wound by a doctor or nurse? | | | | | | | | | | | | | | | | | | | | | | - Yes | | | | | | | | - No | | | | | |
| Have you had to go back to the operating room for treatment of a problem with your wound? | | | | | | | | | | | | | | | | | | | | | | - Yes | | | | | | | | - No | | | | | |
| *Ask the patient:* How happy were you with having your follow-up over the telephone? | | | | | | - Very satisfied | | | | | | | | | | - Satisfied | | | | | | | | | | | - Neither satisfied nor dissatisfied | | | | | | | | |
|  |  |  |  |  |  | - Very unsatisfied | | | | | | | | | | - Unsatisfied | | | | | | | | | | |  |  |  |  |  |  |  |  |  |
| **Before you end the call,** inform the patient that **this telephone questionnaire will not replace the 30-day in-person wound assessment** required as part of the FALCON trial, and **they must still have their 30-day** FALCON **follow-up appointment**. | | | | | | | | | | | | | | | | | | | | | | | | | | | | | | | | | | | |
| Time taken to complete telephone questionnaire | | | | | | - <10 minutes | | | | | | | | | | | | - 11-20 minutes | | | | | | | | | | | | | | | | | |
|  |  |  |  |  |  | - 21-30 minutes | | | | | | | | | | | | - >30 minutes | | | | | | | | | | | | | | | | | |
| Please add any further comments or details of the telephone follow-up here: | | | | | | | | | | | | | | | | | | | | | | | | | | | | | | | | | | | |
| **Form completed by** | | | | | | | | | | | | | | | | | | | | | | | | | | | | | | | | | | | |
| Job role | - Surgeon | - Other doctor | | | - Nurse | | | | | | | | - Other (please specify): _____________ | | | | | | | | | | | | | | | | | | | | | | |
| Print full name |  | | | | | | | | | | | | | | | | | | | | | | | | | | | | | | | | | | |
| Signature |  | | | Date form completed | | | | | | | | | | | | | d | | | | | | d | | m | m | | y | | | | y | y | | y |

Please note this questionnaire is licensed from Oxford University Innovation (OUI) outcomes group. Please contact OUI to obtain a free license to use this questionnaire for research purposes.

Appendix E. Monitoring and quality assurance of WHQ delivery

A monitoring call was performed after the local researcher had completed the WHQ for 5 to 10 patients for quality assurance. Where recordings were available during the pilot, these were reviewed by a member of the SMG fluent in the target language. A WhatsApp group was also created for all site investigators participating in each country to share early experience and best practice during the pilot phase.

Appendix F. Methodological adaptation to the SARS-CoV-2 pandemic

This study ran over the emergence of the SARS-CoV-2 pandemic. This had several operational consequences. Firstly, sites were asked to extend their recruitment where this was possible, in case fewer patients were able to return for in-person FALCON trial follow-up. Secondly, some centres chose to administer the WHQ consecutive patients that were farther away from their date of surgery (i.e., with a longer interval between FALCON trial follow-up and WHQ administration), handled in sensitivity analyses for the primary outcome measure. Thirdly, sites were allowed to pause and restart recruitment to TALON if needed, so long as when the site was ‘live’ consecutive patients had attempted WHQ follow-up.

Appendix G. Protocol sample size considerations (29)

|  | | | | | **Precision around test accuracy measures** | |
| --- | --- | --- | --- | --- | --- | --- |
| **Patients recruited to FALCON trial** | **Patients retained in FALCON trial follow-up** | **In-person FALCON trial follow-up** | **Patients with SSI**^∅^ | **Patients without SSI**^♦^ | **Sensitivity (95% C.I.)** | **Specificity (95% C.I.)** |
| 714 | 607 | 516 | 108 | 408 | 0.92  (0.85-0.96) | 0.95  (0.93-0.97) |
| 571 | 485 | 412 | 87 | 325 | 0.92  (0.84-0.97) | 0.95  (0.92-0.97) |
| 428 | 364 | 309 | 65 | 244 | 0.92  (0.83-0.97) | 0.95  (0.92-0.97) |
| 285 | 242 | 206 | 43 | 163 | 0.92  (0.81-0.99) | 0.95  (0.91-0.98) |

^∅^0.21 * number in-person FALCON trial follow-up. ^♦^0.79 * number in-person FALCON trial follow-up. Estimates around sensitivity and specificity were derived from a UK validation study of the WHQ (37)

Appendix H. Changes to published Statistical Analysis Plan

Some small, iterative changes were required to the published statistical analysis plan (available at: <https://globalsurg.org/resources/phd-research-projects/talon/>) related to the SARS-CoV-2 pandemic, the sample size of proposed comparator groups, and patterns observed in the data. There were no major changes to the primary comparison groups, definition of outcomes, primary analysis methods or handling of missing data.

These included:

1. The number of events and non-events was too small per country to justify per-country analyses. As such:
   1. Cut-off WHQ point scores were presented overall and not by county
   2. Subgroup analyses were presented by country income group rather than by individual country
2. Multiple comparisons of patient characteristics and outcomes between urban versus rural patient home location were too extensive for a single peer-reviewed publication. We presented a subgroup analysis of the model discrimination by urban versus rural settings, and will explore patient home location further in future work.
3. We did not anticipate a significant proportion of the cohort to be outside of the protocol timing window for administration of the WHQ. Due to the SARS-CoV-2 pandemic, we relaxed the pre-specified time window to allow flexibility for overburdened site investigators and a time of system strain, and presented the primary analysis per-protocol, with a sensitivity analysis including all patients including those out-of-protocol.
4. We did not anticipate a large proportion of the cohort to not receive the index test (in-person FALCON trial follow-up), but face-to-face follow-up was deemed not to be safe or feasible in many settings during the SARS-CoV-2 pandemic. We therefore introduced an inverse probability weighted sensitivity analysis to account for a risk of verification bias.
5. In response to investigator and CEI partner feedback, we added two exploratory subgroup analyses: (1) formal versus adhoc translation of the WHQ and (2) mild SSI only (i.e., no reoperation)

Appendix I. Co-produced toolkit for optimising postoperative telephone follow-up


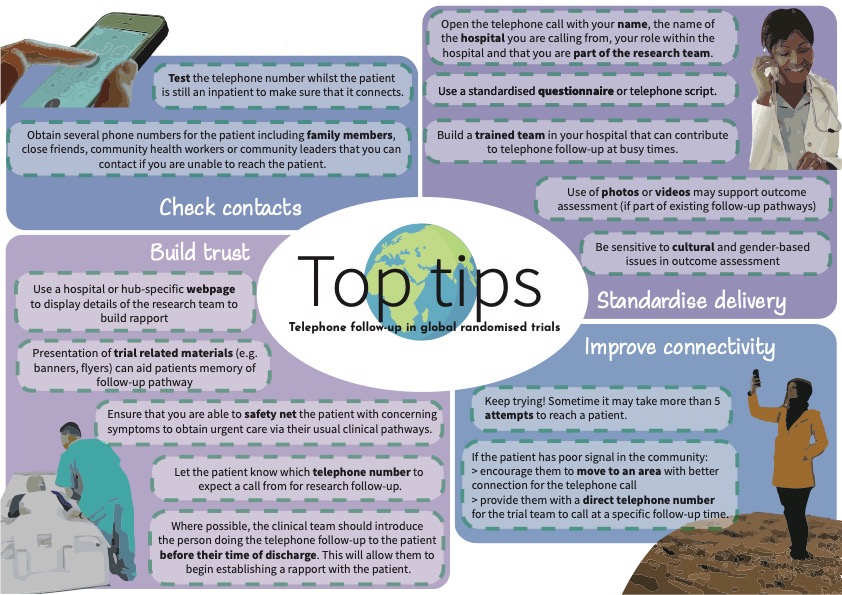


Download the full toolkit presentation at: <https://bit.ly/TALONtips>

Supplementary table 1. Baseline demographics by country (N=1196)

|  |  | **Country** | | | | | | |  |
| --- | --- | --- | --- | --- | --- | --- | --- | --- | --- |
| **Factor** | **Levels** | **Ghana N=532** | **South Africa N=60** | **India N=120** | **Benin N=103** | **Mexico N=216** | **Rwanda N=54** | **Nigeria N=111** | **Total N=1196** |
| FALCON trial follow-up | In-person | 199 (37.4) | 27 (45.0) | 76 (63.3) | 82 (79.6) | 129 (59.7) | 35 (64.8) | 40 (36.0) | 588 (49.2) |
|  | Telephone only | 333 (62.6) | 33 (55.0) | 44 (36.7) | 21 (20.4) | 87 (40.3) | 19 (35.2) | 71 (64.0) | 608 (50.8) |
| Timing of  telephone WHQ | Per protocol | 517 (97.2) | 53 (88.3) | 29 (24.2) | 100 (97.1) | 17 (7.9) | 54 (100.0) | 108 (97.3) | 878 (73.4) |
|  | Outside of protocol | 2 (0.4) | 0 (0.0) | 91 (75.8) | 0 (0.0) | 112 (51.9) | 0 (0.0) | 0 (0.0) | 205 (17.1) |
|  | (Missing) | 13 (2.4) | 7 (11.7) | 0 (0.0) | 3 (2.9) | 87 (40.3) | 0 (0.0) | 3 (2.7) | 113 (9.4) |
| Home location | Urban | 299 (56.2) | 52 (86.7) | 70 (58.3) | 92 (89.3) | 98 (45.4) | 8 (14.8) | 88 (79.3) | 707 (59.1) |
|  | Rural | 221 (41.5) | 2 (3.3) | 49 (40.8) | 8 (7.8) | 31 (14.4) | 46 (85.2) | 23 (20.7) | 380 (31.8) |
|  | (Missing) | 12 (2.3) | 6 (10.0) | 1 (0.8) | 3 (2.9) | 87 (40.3) | 0 (0.0) | 0 (0.0) | 109 (9.1) |
| Age (years) | <18 | 68 (12.8) | 0 (0.0) | 2 (1.7) | 0 (0.0) | 2 (0.9) | 1 (1.9) | 21 (18.9) | 94 (7.9) |
|  | 18-39 | 261 (49.1) | 32 (53.3) | 80 (66.7) | 85 (82.5) | 137 (63.4) | 32 (59.3) | 43 (38.7) | 670 (56.0) |
|  | 40-59 | 140 (26.3) | 20 (33.3) | 26 (21.7) | 13 (12.6) | 56 (25.9) | 10 (18.5) | 29 (26.1) | 294 (24.6) |
|  | 60-79 | 56 (10.5) | 8 (13.3) | 12 (10.0) | 5 (4.9) | 17 (7.9) | 9 (16.7) | 15 (13.5) | 122 (10.2) |
|  | ≥80 | 7 (1.3) | 0 (0.0) | 0 (0.0) | 0 (0.0) | 4 (1.9) | 2 (3.7) | 3 (2.7) | 16 (1.3) |
| Sex | Male | 350 (65.8) | 37 (61.7) | 32 (26.7) | 57 (55.3) | 39 (18.1) | 36 (66.7) | 64 (57.7) | 615 (51.4) |
|  | Female | 182 (34.2) | 23 (38.3) | 88 (73.3) | 46 (44.7) | 177 (81.9) | 18 (33.3) | 47 (42.3) | 581 (48.6) |
| Level of  education | Below high school level | 337 (64.9) | 15 (27.8) | 64 (54.2) | 29 (29.0) | 24 (18.6) | 42 (77.8) | 46 (41.4) | 557 (51.3) |
|  | High school or above | 182 (35.1) | 39 (72.2) | 54 (45.8) | 71 (71.0) | 105 (81.4) | 12 (22.2) | 65 (58.6) | 528 (48.7) |
| Known diabetes | Yes | 11 (2.1) | 2 (3.3) | 7 (5.8) | 1 (1.0) | 16 (7.4) | 1 (1.9) | 2 (1.8) | 40 (3.3) |
|  | No | 521 (97.9) | 58 (96.7) | 113 (94.2) | 102 (99.0) | 200 (92.6) | 53 (98.1) | 109 (98.2) | 1156 (96.7) |
| HIV status | Known negative | 42 (7.9) | 11 (18.3) | 114 (95.0) | 4 (3.9) | 63 (29.2) | 40 (74.1) | 70 (63.1) | 344 (28.8) |
|  | Known positive | 4 (0.8) | 14 (23.3) | 1 (0.8) | 0 (0.0) | 3 (1.4) | 1 (1.9) | 0 (0.0) | 23 (1.9) |
|  | Status not known | 486 (91.4) | 35 (58.3) | 5 (4.2) | 99 (96.1) | 150 (69.4) | 13 (24.1) | 41 (36.9) | 829 (69.3) |
| Smoking status | Never smoked | 504 (94.7) | 40 (66.7) | 113 (94.2) | 100 (97.1) | 187 (86.6) | 46 (85.2) | 99 (89.2) | 1089 (91.1) |
|  | Ex-smoker | 16 (3.0) | 11 (18.3) | 6 (5.0) | 0 (0.0) | 24 (11.1) | 5 (9.3) | 4 (3.6) | 66 (5.5) |
|  | Current smoker | 12 (2.3) | 9 (15.0) | 1 (0.8) | 3 (2.9) | 5 (2.3) | 3 (5.6) | 8 (7.2) | 41 (3.4) |
| Urgency | Elective (planned) | 27 (5.1) | 8 (13.3) | 38 (31.7) | 0 (0.0) | 171 (79.2) | 3 (5.6) | 21 (18.9) | 268 (22.4) |
|  | Emergency (unplanned) | 505 (94.9) | 52 (86.7) | 82 (68.3) | 103 (100.0) | 45 (20.8) | 51 (94.4) | 90 (81.1) | 928 (77.6) |
| Indication | Malignant disease | 23 (4.3) | 5 (8.3) | 20 (16.7) | 2 (1.9) | 13 (6.0) | 2 (3.7) | 20 (18.0) | 85 (7.1) |
|  | Benign disease | 485 (91.2) | 39 (65.0) | 31 (25.8) | 100 (97.1) | 123 (56.9) | 46 (85.2) | 83 (74.8) | 907 (75.8) |
|  | Trauma | 18 (3.4) | 13 (21.7) | 0 (0.0) | 1 (1.0) | 1 (0.5) | 5 (9.3) | 6 (5.4) | 44 (3.7) |
|  | Obstetric | 5 (0.9) | 3 (5.0) | 69 (57.5) | 0 (0.0) | 79 (36.6) | 1 (1.9) | 2 (1.8) | 159 (13.3) |
|  | (Missing) | 1 (0.2) | 0 (0.0) | 0 (0.0) | 0 (0.0) | 0 (0.0) | 0 (0.0) | 0 (0.0) | 1 (0.1) |
| Operation  location | Foregut | 183 (34.4) | 11 (18.3) | 12 (10.0) | 8 (7.8) | 70 (32.4) | 9 (16.7) | 22 (19.8) | 315 (26.3) |
|  | Hindgut | 45 (8.5) | 9 (15.0) | 21 (17.5) | 2 (1.9) | 11 (5.1) | 3 (5.6) | 21 (18.9) | 112 (9.4) |
|  | Appendix | 166 (31.2) | 19 (31.7) | 6 (5.0) | 88 (85.4) | 14 (6.5) | 11 (20.4) | 28 (25.2) | 332 (27.8) |
|  | Urogenital | 9 (1.7) | 1 (1.7) | 71 (59.2) | 0 (0.0) | 110 (50.9) | 4 (7.4) | 9 (8.1) | 204 (17.1) |
|  | Other | 128 (24.1) | 19 (31.7) | 10 (8.3) | 5 (4.9) | 11 (5.1) | 27 (50.0) | 28 (25.2) | 228 (19.1) |
|  | (Missing) | 1 (0.2) | 1 (1.7) | 0 (0.0) | 0 (0.0) | 0 (0.0) | 0 (0.0) | 3 (2.7) | 5 (0.4) |
| ASA grade | Grade I | 320 (60.2) | 30 (50.0) | 43 (35.8) | 79 (76.7) | 58 (26.9) | 17 (31.5) | 13 (11.7) | 560 (46.8) |
|  | Grade II | 158 (29.7) | 20 (33.3) | 69 (57.5) | 22 (21.4) | 145 (67.1) | 20 (37.0) | 36 (32.4) | 470 (39.3) |
|  | Grade III | 50 (9.4) | 8 (13.3) | 6 (5.0) | 2 (1.9) | 11 (5.1) | 17 (31.5) | 56 (50.5) | 150 (12.5) |
|  | Grade IV/V | 3 (0.6) | 2 (3.3) | 2 (1.7) | 0 (0.0) | 2 (0.9) | 0 (0.0) | 6 (5.4) | 15 (1.3) |
|  | (Missing) | 1 (0.2) | 0 (0.0) | 0 (0.0) | 0 (0.0) | 0 (0.0) | 0 (0.0) | 0 (0.0) | 1 (0.1) |
| WHO checklist | Yes | 500 (94.0) | 57 (95.0) | 120 (100.0) | 102 (99.0) | 210 (97.2) | 53 (98.1) | 69 (62.2) | 1111 (92.9) |
|  | No | 32 (6.0) | 3 (5.0) | 0 (0.0) | 1 (1.0) | 6 (2.8) | 1 (1.9) | 42 (37.8) | 85 (7.1) |
| Operation grade | Intermediate/Minor | 185 (35.8) | 20 (34.5) | 8 (6.7) | 88 (85.4) | 18 (8.4) | 12 (24.5) | 29 (26.9) | 360 (30.8) |
|  | Major | 332 (64.2) | 38 (65.5) | 111 (93.3) | 15 (14.6) | 197 (91.6) | 37 (75.5) | 79 (73.1) | 809 (69.2) |
| Contamination | Clean/Clean-contaminated | 75 (14.1) | 14 (23.3) | 89 (74.2) | 14 (13.6) | 200 (92.6) | 1 (1.9) | 14 (12.6) | 407 (34.0) |
|  | Contaminated | 225 (42.3) | 30 (50.0) | 26 (21.7) | 40 (38.8) | 14 (6.5) | 26 (48.1) | 52 (46.8) | 413 (34.5) |
|  | Dirty | 230 (43.2) | 16 (26.7) | 5 (4.2) | 49 (47.6) | 2 (0.9) | 27 (50.0) | 45 (40.5) | 374 (31.3) |
|  | (Missing) | 2 (0.4) | 0 (0.0) | 0 (0.0) | 0 (0.0) | 0 (0.0) | 0 (0.0) | 0 (0.0) | 2 (0.2) |
| Approach | Open midline | 435 (81.8) | 48 (80.0) | 41 (34.2) | 34 (33.0) | 43 (19.9) | 49 (90.7) | 83 (74.8) | 733 (61.3) |
|  | Open non-midline | 96 (18.0) | 11 (18.3) | 74 (61.7) | 69 (67.0) | 169 (78.2) | 5 (9.3) | 28 (25.2) | 452 (37.8) |
|  | Laparoscopic attempted | 0 (0.0) | 1 (1.7) | 5 (4.2) | 0 (0.0) | 4 (1.9) | 0 (0.0) | 0 (0.0) | 10 (0.8) |
|  | (Missing) | 1 (0.2) | 0 (0.0) | 0 (0.0) | 0 (0.0) | 0 (0.0) | 0 (0.0) | 0 (0.0) | 1 (0.1) |
| Stoma formation | Yes | 15 (2.8) | 4 (6.7) | 17 (14.2) | 0 (0.0) | 10 (4.6) | 6 (11.1) | 7 (6.3) | 59 (4.9) |
|  | No | 514 (96.6) | 55 (91.7) | 102 (85.0) | 103 (100.0) | 205 (94.9) | 47 (87.0) | 102 (91.9) | 1128 (94.3) |
|  | (Missing) | 3 (0.6) | 1 (1.7) | 1 (0.8) | 0 (0.0) | 1 (0.5) | 1 (1.9) | 2 (1.8) | 9 (0.8) |

*FALCON, a stratified, pragmatic, multi-centre, 2x2 factorial trial testing two measures (skin preparation and antimicrobial sutures) to reduce superficial or deep skin infection following abdominal surgery in seven low- and middle-income countries (NCT03700749) (2). WHQ = Wound Healing Questionnaire. HIV = Human immunodeficiency virus.

Supplementary table 2. Comparison of patients included in TALON study within a trial and FALCON trial overall

| **Factor** | **Levels** | **Included in TALON study within a trial**  **N=1088** | **Included in FALCON study overall**  **N=5788** |
| --- | --- | --- | --- |
| Country | Ghana | 520 (47.8) | 1424 (24.6) |
|  | South Africa | 54 (5.0) | 177 (3.1) |
|  | India | 120 (11.0) | 800 (13.8) |
|  | Benin | 100 (9.2) | 145 (2.5) |
|  | Mexico | 129 (11.9) | 238 (4.1) |
|  | Rwanda | 54 (5.0) | 839 (14.5) |
|  | Nigeria | 111 (10.2) | 2165 (37.4) |
| Age (years) | <18 | 90 (8.3) | 811 (14.0) |
|  | 18-39 | 606 (55.7) | 3183 (55.0) |
|  | 40-59 | 262 (24.1) | 1186 (20.5) |
|  | 60-79 | 115 (10.6) | 534 (9.2) |
|  | ≥80 | 15 (1.4) | 74 (1.3) |
| Sex | Male | 582 (53.5) | 2200 (38.4) |
|  | Female | 506 (46.5) | 3534 (61.6) |
| Known diabetes | Yes | 33 (3.0) | 217 (3.8) |
|  | No | 1055 (97.0) | 5513 (96.2) |
| HIV status | Known negative | 319 (29.3) | 3207 (55.9) |
|  | Known positive | 21 (1.9) | 128 (2.2) |
|  | Status not known | 748 (68.8) | 2399 (41.8) |
| Smoking status | Never smoked | 996 (91.5) | 5344 (93.2) |
|  | Ex-smoker | 53 (4.9) | 220 (3.8) |
|  | Current smoker | 39 (3.6) | 168 (2.9) |
| Urgency | Elective (planned) | 197 (18.1) | 1915 (33.1) |
|  | Emergency (unplanned) | 891 (81.9) | 3873 (66.9) |
| Indication | Malignant disease | 80 (7.4) | 442 (7.8) |
|  | Benign disease | 834 (76.7) | 3326 (59.0) |
|  | Trauma | 40 (3.7) | 442 (7.8) |
|  | Obstetric | 133 (12.2) | 1702 (30.2) |
|  | (Missing) | 1 (0.1) | 1 (0.0) |
| Operation location | Foregut | 272 (25.0) | 1084 (18.7) |
|  | Hindgut | 106 (9.7) | 529 (9.1) |
|  | Appendix | 324 (29.8) | 933 (16.1) |
|  | Urogenital | 162 (14.9) | 2255 (39.0) |
|  | Other | 219 (20.1) | 834 (14.4) |
|  | (Missing) | 5 (0.5) | 153 (2.6) |
| ASA grade | Grade I | 519 (47.7) | 2540 (45.0) |
|  | Grade II | 415 (38.1) | 2121 (37.6) |
|  | Grade III | 142 (13.1) | 788 (14.0) |
|  | Grade IV/V | 11 (1.0) | 196 (3.4) |
|  | (Missing) | 1 (0.1) | 3 (0.0) |
| WHO Checklist | Yes | 1006 (92.5) | 680 (12.0) |
|  | No | 82 (7.5) | 4965 (88.0) |
| Operation grade | Intermediate/Minor | 350 (33.0) | 1023 (18.4) |
|  | Major | 711 (67.0) | 4534 (81.6) |
| Contamination | Clean/Clean-contaminated | 322 (29.6) | 3123 (55.4) |
|  | Contaminated | 399 (36.7) | 1235 (21.9) |
|  | Dirty | 365 (33.5) | 1282 (22.7) |
|  | (Missing) | 2 (0.2) | 3 (0.0) |
| Approach | Open midline | 707 (65.0) | 2764 (49.0) |
|  | Open non-midline | 371 (34.1) | 2827 (50.1) |
|  | Laparoscopic attempted | 9 (0.8) | 51 (0.8) |
|  | (Missing) | 1 (0.1) | 1 (0.1) |
| Stoma formation | Yes | 55 (5.1) | 290 (5.1) |
|  | No | 1025 (94.2) | 5318 (94.8) |
|  | (Missing) | 8 (0.7) | 12 (0.1) |

Supplementary table 3. Feasibility outcomes by country (N=1196)

|  |  | **Country** | | | | | | |  |  |
| --- | --- | --- | --- | --- | --- | --- | --- | --- | --- | --- |
| **Factor** | **Levels** | **Ghana N=532** | **South Africa N=60** | **India N=120** | **Benin N=103** | **Mexico N=216** | **Rwanda N=54** | **Nigeria N=111** | **Total N=1196** | **P-value** |
| Successful telephone contact | No | 12 (2.3) | 6 (10.0) | 0 (0.0) | 3 (2.9) | 87 (40.3) | 0 (0.0) | 0 (0.0) | 108 (9.0) | <0.001 |
|  | Yes | 520 (97.7) | 54 (90.0) | 120 (100.0) | 100 (97.1) | 129 (59.7) | 54 (100.0) | 111 (100.0) | 1088 (91.0) |  |
| Attempts at contact | 1 attempt | 145 (49.8) | 25 (41.7) | 28 (68.3) | 0 (0.0) | 10 (62.5) | 12 (22.2) | 47 (48.0) | 267 (47.7) | <0.001 |
|  | 2-3 attempts | 93 (32.0) | 22 (36.7) | 8 (19.5) | 0 (0.0) | 1 (6.2) | 29 (53.7) | 32 (32.7) | 185 (33.0) |  |
|  | 4-5 attempts | 26 (8.9) | 6 (10.0) | 5 (12.2) | 0 (0.0) | 0 (0.0) | 11 (20.4) | 14 (14.3) | 62 (11.1) |  |
|  | >5 attempts | 27 (9.3) | 7 (11.7) | 0 (0.0) | 0 (0.0) | 5 (31.2) | 2 (3.7) | 5 (5.1) | 46 (8.2) |  |
|  | (Missing)* | 241 | 0 | 79 | 103 | 200 | 0 | 13 | 636 |  |
| Patient satisfaction^$^ | Very Satisfied | 243 (84.1) | 42 (77.8) | 13 (28.9) | 0 (0.0) | 1 (9.1) | 39 (72.2) | 55 (56.7) | 393 (71.5) | <0.001 |
|  | Satisfied | 45 (15.6) | 9 (16.7) | 32 (71.1) | 0 (0.0) | 10 (90.9) | 15 (27.8) | 41 (42.3) | 152 (27.6) |  |
|  | Neutral | 1 (0.3) | 3 (5.6) | 0 (0.0) | 0 (0.0) | 0 (0.0) | 0 (0.0) | 1 (1.0) | 5 (0.9) |  |
|  | Unsatisfied | 0 (0.0) | 0 (0.0) | 0 (0.0) | 0 (0.0) | 0 (0.0) | 0 (0.0) | 0 (0.0) | 0 (0.0) |  |
|  | Very unsatisfied | 0 (0.0) | 0 (0.0) | 0 (0.0) | 0 (0.0) | 0 (0.0) | 0 (0.0) | 0 (0.0) | 0 (0.0) |  |
|  | (Missing)* | 243 | 6 | 75 | 103 | 205 | 0 | 14 | 646 |  |

*Question added after pilot phase in response to Community Engagement and Involvement group feedback, so not available for patients recruited in pilot phase. ^$^ Represents patient’s self-reported satisfaction with telephone administration of the WHQ.

Supplementary table 4. Factors associated with successful telephone contact in a multivariable model (Figure 2).

|  |  | **Successful telephone contact** | |  |  |
| --- | --- | --- | --- | --- | --- |
| **Factor** | **Levels** | **No** | **Yes** | **OR (univariable)** | **OR (multivariable)** |
| Timing of WHQ | Per protocol | 25 (2.8) | 881 (97.2) | - | - |
|  | Out of protocol | 83 (28.8) | 205 (71.2) | 0.07 (0.04-0.11, p<0.001) | ***0.11 (0.05-0.24, p<0.001)*** |
| *Patient factors* | | | | | |
| Age | <18 years | 4 (4.3) | 90 (95.7) | - | - |
|  | 18-39 | 64 (9.6) | 606 (90.4) | 0.42 (0.13-1.05, p=0.101) | 1.39 (0.37-4.14, p=0.589) |
|  | 40-59 | 32 (10.9) | 262 (89.1) | 0.36 (0.11-0.95, p=0.063) | 1.36 (0.34-4.41, p=0.630) |
|  | 60-79 | 7 (5.7) | 115 (94.3) | 0.73 (0.19-2.49, p=0.624) | 3.14 (0.64-14.56, p=0.144) |
|  | ≥80 | 1 (6.2) | 15 (93.8) | 0.67 (0.09-13.51, p=0.725) | 4.59 (0.34-128.73, p=0.283) |
| Sex | Male | 33 (5.4) | 582 (94.6) | - | - |
|  | Female | 75 (12.9) | 506 (87.1) | 0.38 (0.25-0.58, p<0.001) | 1.97 (0.98-4.11, p=0.062) |
| Urgency | Elective (planned) | 71 (26.5) | 197 (73.5) | - | - |
|  | Emergency (unplanned) | 37 (4.0) | 891 (96.0) | 8.68 (5.70-13.41, p<0.001) | 1.89 (0.99-3.65, p=0.055) |
| Indication | Malignant disease | 5 (5.9) | 80 (94.1) | - | - |
|  | Benign disease | 73 (8.0) | 834 (92.0) | 0.71 (0.25-1.65, p=0.480) | 0.86 (0.23-2.81, p=0.815) |
|  | Trauma | 4 (9.1) | 40 (90.9) | 0.63 (0.16-2.64, p=0.501) | 0.31 (0.05-1.77, p=0.177) |
|  | Obstetric | 26 (16.4) | 133 (83.6) | 0.32 (0.10-0.80, p=0.025) | 3.38 (0.66-15.95, p=0.130) |
| ASA grade | Grade I | 41 (7.3) | 519 (92.7) | - | - |
|  | Grade II | 55 (11.7) | 415 (88.3) | 0.60 (0.39-0.91, p=0.017) | 1.13 (0.66-1.94, p=0.655) |
|  | Grade III | 8 (5.3) | 142 (94.7) | 1.40 (0.68-3.29, p=0.396) | 0.86 (0.34-2.37, p=0.757) |
|  | Grade IV/V | 4 (26.7) | 11 (73.3) | 0.22 (0.07-0.81, p=0.012) | 0.27 (0.06-1.45, p=0.106) |
| *Operation factors* | | | | | |
| Operation grade | Intermediate/Minor | 10 (2.8) | 350 (97.2) | - | - |
|  | Major | 98 (12.1) | 711 (87.9) | 0.21 (0.10-0.38, p<0.001) | 1.32 (0.17-7.11, p=0.761) |
| Operation location | Foregut | 43 (13.7) | 272 (86.3) | - | - |
|  | Hindgut | 6 (5.4) | 106 (94.6) | 2.79 (1.24-7.49, p=0.023) | 1.83 (0.59-6.58, p=0.321) |
|  | Appendix | 8 (2.4) | 324 (97.6) | 6.40 (3.12-14.91, p<0.001) | 3.67 (0.40-24.79, p=0.204) |
|  | Urogenital | 42 (20.6) | 162 (79.4) | 0.61 (0.38-0.97, p=0.038) | 1.01 (0.42-2.47, p=0.981) |
|  | Other | 9 (3.9) | 219 (96.1) | 3.85 (1.92-8.58, p<0.001) | 1.61 (0.63-4.52, p=0.339) |
| Contamination | Clean/Clean-contaminated | 85 (20.9) | 322 (79.1) | - | - |
|  | Contaminated | 14 (3.4) | 399 (96.6) | 7.52 (4.33-14.05, p<0.001) | 2.07 (0.89-4.88, p=0.091) |
|  | Dirty | 9 (2.4) | 365 (97.6) | 10.71 (5.59-23.19, p<0.001) | 2.17 (0.78-6.23, p=0.140) |
| Approach | Open midline | 26 (3.5) | 707 (96.5) | - | - |
|  | Open non-midline | 81 (17.9) | 371 (82.1) | 0.17 (0.10-0.26, p<0.001) | ***0.36 (0.17-0.73, p=0.005)*** |
|  | Laparoscopic attempted | 1 (10.0) | 9 (90.0) | 0.33 (0.06-6.22, p=0.303) | 2.50 (0.33-52.24, p=0.438) |
| Surgical site infection status | | | | | |
| SSI diagnosis (reference test) | No | 90 (9.1) | 897 (90.9) | - | - |
|  | Yes | 18 (8.6) | 191 (91.4) | 1.06 (0.64-1.86, p=0.817) | ***0.42 (0.20-0.92, p=0.026)*** |
| Systemic symptoms of SSI | No | 99 (9.1) | 986 (90.9) | - | - |
|  | Yes | 9 (8.1) | 102 (91.9) | 1.14 (0.59-2.48, p=0.722) | 0.95 (0.33-2.99, p=0.934) |
| Reoperation for SSI | No | 104 (9.1) | 1041 (90.9) | - | - |
|  | Yes | 4 (8.2) | 45 (91.8) | 1.12 (0.45-3.78, p=0.826) | 0.90 (0.24-4.15, p=0.889) |

Number in dataframe = 1196, Number in model = 1162, Missing = 34, AIC = 500, C-statistic = 0.917

Supplementary table 5. Differences in baseline demographics between patients contactable by telephone that had in-person FALCON trial follow-up and telephone FALCON trial follow-up (N=1088).

| **Factor** | **Levels** | **In-person FALCON trial follow-up**  **N=531** | **Telephone FALCON trial follow-up only**  **N=557** | **Total**  **N=1088** | **P-value** |
| --- | --- | --- | --- | --- | --- |
| Country | Ghana | 195 (36.7) | 325 (58.3) | 520 (47.8) | <0.001 |
|  | South Africa | 25 (4.7) | 29 (5.2) | 54 (5.0) |  |
|  | India | 76 (14.3) | 44 (7.9) | 120 (11.0) |  |
|  | Benin | 80 (15.1) | 20 (3.6) | 100 (9.2) |  |
|  | Mexico | 80 (15.1) | 49 (8.8) | 129 (11.9) |  |
|  | Rwanda | 35 (6.6) | 19 (3.4) | 54 (5.0) |  |
|  | Nigeria | 40 (7.5) | 71 (12.7) | 111 (10.2) |  |
| Timing of telephone WHQ | Per protocol | 388 (73.1) | 490 (88.0) | 878 (80.7) | <0.001 |
|  | Outside of protocol | 141 (26.6) | 64 (11.5) | 205 (18.8) |  |
|  | (Missing) | 2 (0.4) | 3 (0.5) | 5 (0.5) |  |
| Home location | Urban | 367 (69.1) | 340 (61.0) | 707 (65.0) | 0.006 |
|  | Rural | 163 (30.7) | 217 (39.0) | 380 (34.9) |  |
|  | (Missing) | 1 (0.2) | 0 (0.0) | 1 (0.1) |  |
| Age (years) | <18 | 42 (7.9) | 48 (8.6) | 90 (8.3) | 0.27 |
|  | 18-39 | 313 (58.9) | 293 (52.6) | 606 (55.7) |  |
|  | 40-59 | 121 (22.8) | 141 (25.3) | 262 (24.1) |  |
|  | 60-79 | 48 (9.0) | 67 (12.0) | 115 (10.6) |  |
|  | ≥80 | 7 (1.3) | 8 (1.4) | 15 (1.4) |  |
| Sex | Male | 255 (48.0) | 327 (58.7) | 582 (53.5) | <0.001 |
|  | Female | 276 (52.0) | 230 (41.3) | 506 (46.5) |  |
| Level of education | Below high school level | 247 (46.8) | 310 (55.7) | 557 (51.3) | 0.004 |
|  | High school or above | 281 (53.2) | 247 (44.3) | 528 (48.7) |  |
| Known diabetes | Yes | 16 (3.0) | 17 (3.1) | 33 (3.0) | 1 |
|  | No | 515 (97.0) | 540 (96.9) | 1055 (97.0) |  |
| HIV status | Known negative | 197 (37.1) | 122 (21.9) | 319 (29.3) | <0.001 |
|  | Known positive | 13 (2.4) | 8 (1.4) | 21 (1.9) |  |
|  | Status not known | 321 (60.5) | 427 (76.7) | 748 (68.8) |  |
| Smoking status | Never smoked | 482 (90.8) | 514 (92.3) | 996 (91.5) | 0.431 |
|  | Ex-smoker | 26 (4.9) | 27 (4.8) | 53 (4.9) |  |
|  | Current smoker | 23 (4.3) | 16 (2.9) | 39 (3.6) |  |
| Urgency | Elective (planned) | 101 (19.0) | 96 (17.2) | 197 (18.1) | 0.493 |
|  | Emergency (unplanned) | 430 (81.0) | 461 (82.8) | 891 (81.9) |  |
| Indication | Malignant disease | 28 (5.3) | 52 (9.3) | 80 (7.4) | <0.001 |
|  | Benign disease | 388 (73.1) | 446 (80.1) | 834 (76.7) |  |
|  | Trauma | 15 (2.8) | 25 (4.5) | 40 (3.7) |  |
|  | Obstetric | 100 (18.8) | 33 (5.9) | 133 (12.2) |  |
|  | (Missing) | 0 (0.0) | 1 (0.2) | 1 (0.1) |  |
| Operation location | Foregut | 127 (23.9) | 145 (26.0) | 272 (25.0) | <0.001 |
|  | Hindgut | 46 (8.7) | 60 (10.8) | 106 (9.7) |  |
|  | Appendix | 161 (30.3) | 163 (29.3) | 324 (29.8) |  |
|  | Urogenital | 117 (22.0) | 45 (8.1) | 162 (14.9) |  |
|  | Other | 77 (14.5) | 142 (25.5) | 219 (20.1) |  |
|  | (Missing) | 3 (0.6) | 2 (0.4) | 5 (0.5) |  |
| ASA grade | Grade I | 266 (50.1) | 253 (45.4) | 519 (47.7) | 0.032 |
|  | Grade II | 204 (38.4) | 211 (37.9) | 415 (38.1) |  |
|  | Grade III | 54 (10.2) | 88 (15.8) | 142 (13.1) |  |
|  | Grade IV/V | 7 (1.3) | 4 (0.7) | 11 (1.0) |  |
|  | (Missing) | 0 (0.0) | 1 (0.2) | 1 (0.1) |  |
| WHO Checklist | Yes | 507 (95.5) | 499 (89.6) | 1006 (92.5) | <0.001 |
|  | No | 24 (4.5) | 58 (10.4) | 82 (7.5) |  |
| Operation grade | Intermediate/Minor | 166 (32.0) | 184 (33.9) | 350 (33.0) | 0.568 |
|  | Major | 352 (68.0) | 359 (66.1) | 711 (67.0) |  |
| Contamination | Clean/Clean-contaminated | 195 (36.7) | 127 (22.8) | 322 (29.6) | <0.001 |
|  | Contaminated | 166 (31.3) | 233 (41.8) | 399 (36.7) |  |
|  | Dirty | 170 (32.0) | 195 (35.0) | 365 (33.5) |  |
|  | (Missing) | 0 (0.0) | 2 (0.4) | 2 (0.2) |  |
| Approach | Open midline | 289 (54.4) | 418 (75.0) | 707 (65.0) | <0.001 |
|  | Open non-midline | 237 (44.6) | 134 (24.1) | 371 (34.1) |  |
|  | Laparoscopic attempted | 5 (0.9) | 4 (0.7) | 9 (0.8) |  |
|  | (Missing) | 0 (0.0) | 1 (0.2) | 1 (0.1) |  |
| Stoma formation | Yes | 23 (4.3) | 32 (5.7) | 55 (5.1) | 0.355 |
|  | No | 504 (94.9) | 521 (93.5) | 1025 (94.2) |  |
|  | (Missing) | 4 (0.8) | 4 (0.7) | 8 (0.7) |  |

Supplementary table 6. Cross-tabulation of patients WHQ score and whether or not they received a diagnosis of SSI at the in-person assessment 30-days after surgery

1. **Per-protocol analysis (N=388)**

|  | **Global Wound Healing Questionnaire score (index test)** | | | | | | | | | | | | | | | | | | | | | | | | |
| --- | --- | --- | --- | --- | --- | --- | --- | --- | --- | --- | --- | --- | --- | --- | --- | --- | --- | --- | --- | --- | --- | --- | --- | --- | --- |
| **Reference**  **test** | **0** | **1** | **2** | **3** | **4** | **5** | **6** | **7** | **8** | **9** | **10** | **11** | **12** | **13** | **14** | **15** | **16** | **17** | **18** | **19** | **20** | **21** | **22** | **23** | **24** |
| No SSI | 140 | 62 | 44 | 19 | 11 | 2 | 2 | 2 | 5 | 2 | 0 | 0 | 1 | 0 | 1 | 0 | 0 | 0 | 0 | 0 | 0 | 0 | 0 | 0 | 0 |
| Yes SSI | 7 | 8 | 8 | 6 | 5 | 9 | 7 | 3 | 8 | 5 | 4 | 5 | 4 | 4 | 4 | 2 | 3 | 2 | 1 | 0 | 0 | 0 | 0 | 0 | 2 |

1. **Sensitivity analysis including out of protocol (N=531)**

|  | **Global Wound Healing Questionnaire score (index test)** | | | | | | | | | | | | | | | | | | | | | | | | |
| --- | --- | --- | --- | --- | --- | --- | --- | --- | --- | --- | --- | --- | --- | --- | --- | --- | --- | --- | --- | --- | --- | --- | --- | --- | --- |
| **Reference test** | **0** | **1** | **2** | **3** | **4** | **5** | **6** | **7** | **8** | **9** | **10** | **11** | **12** | **13** | **14** | **15** | **16** | **17** | **18** | **19** | **20** | **21** | **22** | **23** | **24** |
| No SSI | 180 | 93 | 72 | 32 | 16 | 6 | 3 | 8 | 7 | 3 | 1 | 0 | 1 | 2 | 2 | 0 | 0 | 0 | 1 | 0 | 0 | 0 | 0 | 0 | 0 |
| Yes SSI | 9 | 8 | 10 | 7 | 5 | 9 | 7 | 3 | 8 | 5 | 6 | 5 | 4 | 4 | 4 | 2 | 3 | 2 | 1 | 0 | 0 | 0 | 0 | 0 | 2 |

Supplementary table 7. Misclassification in patients with a WHQ score of zero (N=147)

|  |  | **Reference test result** | |  |
| --- | --- | --- | --- | --- |
| **Component of in-person FALCON trial follow-up** | **Response levels** | **No SSI N=140** | **Yes SSI N=7** | **Total** |
| Was there redness of the wound? | Yes | 0 (0.0) | ***2 (28.6)*** | 2 (1.4) |
|  | No | 140 (100.0) | 5 (71.4) | 145 (98.6) |
| Did patient have heat of the wound? | Yes | 0 (0.0) | ***3 (42.9)*** | 3 (2.0) |
|  | No | 140 (100.0) | 4 (57.1) | 144 (98.0) |
| Did patient have purulent drainage from the wound? | Yes | 0 (0.0) | ***6 (85.7)*** | 6 (4.1) |
|  | No | 140 (100.0) | 1 (14.3) | 141 (95.9) |
| Was abdominal wound opening present (spontaneously opened or by clinician)? | Yes | 1 (0.7) | ***5 (71.4)*** | 6 (4.1) |
|  | No | 139 (99.3) | 2 (28.6) | 141 (95.9) |
| Was there localised swelling around the wound? | Yes | 0 (0.0) | ***4 (57.1)*** | 4 (2.7) |
|  | No | 140 (100.0) | 3 (42.9) | 143 (97.3) |
| Was there pain or tenderness at the wound? | Yes | 12 (8.6) | ***6 (85.7)*** | 18 (12.2) |
|  | No | 128 (91.4) | 1 (14.3) | 129 (87.8) |
| Did patient have systemic fever (greater than 38 degrees Celsius)? | Yes | 0 (0.0) | ***2 (28.6)*** | 2 (1.4) |
|  | No | 140 (100.0) | 5 (71.4) | 145 (98.6) |
| Has patient been re-admitted? | Yes, not SSI related | 2 (1.4) | *1 (14.3)* | 3 (2.0) |
|  | Yes, for treatment of SSI | 0 (0.0) | 0 (0.0) | 0 (0.0) |
|  | No | 138 (98.6) | 6 (85.7) | 144 (98.0) |
| Has patient been re-operated on? | Yes, not SSI related | 1 (0.7) | *1 (14.3)* | 2 (1.4) |
|  | Yes, for treatment of SSI | 0 (0.0) | 0 (0.0) | 0 (0.0) |
|  | No | 139 (99.3) | 6 (85.7) | 145 (98.6) |
| Was SSI diagnosed by clinician or on imaging? | Yes | 0 (0.0) | ***6 (85.7)*** | 6 (4.1) |
|  | No | 140 (100.0) | 1 (14.3) | 141 (95.9) |

Supplementary table 8. Full summary of diagnostic test accuracy characteristics overall and across subgroups

| **Patient group** | **N=** | **SSI prevalence**^$^  **N=, (%)** | **AUROC** | **Accuracy** | **Sensitivity** | **Specificity** | **PLR** | **NLR** | **PPV** | **NPV** | **WHQ score cut-off^€^** |
| --- | --- | --- | --- | --- | --- | --- | --- | --- | --- | --- | --- |
| **Overall*** | **388** | **97 (25.0%)** | **0.869**  **(0.824-0.914)** | **0.858**  **(0.858-0.859)** | **0.701**  **(0.610-0.792)** | **0.911**  **(0.878-0.9430** | **7.846**  **(5.317-11.579)** | **0.328**  **(0.242-0.446)** | **0.723**  **(0.633-0.814)** | **0.901**  **(0.867-0.935)** | **3.5** |
| *Sensitivity analyses* | | | | | | | | | | | |
| Including out-of-protocol | 531 | 104 (19.6%) | 0.836  (0.788-0.883) | 0.842  (0.841-0.842) | 0.673  (0.583-0.763) | 0.883  (0.852-0.913) | 5.748  (4.289-7.704) | 0.370  (0.280-0.489) | 0.583  (0.495-0.672) | 0.917  (0.891-0.944) | 3.5 |
| No discharge SSI | 300 | 32 (10.1%) | 0.863  (0.790-0.937) | 0.923  (0.923-0.924) | 0.625  (0.457-0.793) | 0.959  (0.935-0.983) | 15.227  (8.046-28.818) | 0.391  (0.250-0.612) | 0.645  (0.477-0.814) | 0.955  (0.931-0.980) | 4.5 |
| *Subgroup analyses* | | | | | | | | | | | |
| Patient home location | | | | | | | | | | | |
| *Urban* | 266 | 66 (24.8%) | 0.886  (0.836-0.937) | 0.838  (0.837-0.839) | 0.818  (0.725-0.911) | 0.845  (0.795-0.895) | 5.279  (3.746-7.438) | 0.215  (0.129-0.360) | 0.635  (0.533-0.738) | 0.934  (0.897-0.970) | 2.5 |
| *Rural* | 122 | 31 (25.4%) | 0.818  (0.721-0.914) | 0.877  (0.875-0.879) | 0.613  (0.441-0.784) | 0.967  (0.930-1.004) | 18.591  (5.902-58.564) | 0.400  (0.257-0.624) | 0.864  (0.720-1.007) | 0.880  (0.816-0.944) | 4.5 |
| Country income settings | | | | | | | | | | | |
| *Upper-middle* | 39 | 10 (25.6%) | 0.888  (0.741-1.000) | 0.846  (0.840-0.853) | 0.900  (0.714-1.086) | 0.828  (0.690-0.965) | 5.220  (2.290-11.896) | 0.121  (0.019-0.782) | 0.643  (0.392-0.894) | 0.960  (0.883-1.037) | 2.5 |
| *Lower-middle* | 314 | 74 (23.6%) | 0.868  (0.817-0.918) | 0.866  (0.866-0.867) | 0.689  (0.584-0.795) | 0.921  (0.887-0.955) | 8.706  (5.508-13.760) | 0.338  (0.240-0.475) | 0.729  (0.624-0.833) | 0.906  (0.869-0.942) | 3.5 |
| *Low* | 35 | 13 (37.1%) | 0.892  (0.748-1.000) | 0.829  (0.837-0.837) | 0.923  (0.778-1.068) | 0.773  (0.598-0.948) | 4.062  (1.850-8.916) | 0.100  (0.015-0.663) | 0.706  (0.489-0.922) | 0.944  (0.839-1.050) | 1.5 |
| Patient age group | | | | | | | | | | | |
| *>60 years* | 43 | 7 (16.3%) | 0.867  (0.729-1.000) | 0.721  (0.712-0.730) | 0.857  (0.589-1.116) | 0.694  (0.544-0.845) | 2.805  (1.574-5.000) | 0.206  (0.033-1.279) | 0.353  (0.126-0.580) | 0.962  (0.888-1.035) | 1.5 |
| *<60 years* | 345 | 90 (26.1%) | 0.869  (0.821-0.916) | 0.852  (0.851-0.853) | 0.722  (0.630-0.815) | 0.898  (0.861-0.935) | 7.083  (4.814-10.422) | 0.309  (0.221-0.433) | 0.714  (0.621-0.807) | 0.902  (0.865-0.938) | 3.5 |
| Urgency of surgery | | | | | | | | | | | |
| *Emergency* | 364 | 95 (26.1%) | 0.871  (0.826-0.916) | 0.830  (0.829-0.830) | 0.758  (0.672-0.844) | 0.855  (0.813-0.897) | 5.228  (3.828-7.139) | 9.283  (0.198-0.406) | 0.649  (0.560-0.737) | 0.909  (0.874-0.945) | 2.5 |
| *Elective* | 21 | 2 (8.3%) | 0.966  (0.895-1.000) | 0.958  (0.955-0.962) | 1.000  (1.000-1.000) | 0.955  (0.868-1.042) | 22.000  (3.242-149.303) | 0.000  (0.000-0.000) | 0.667  (0.133-1.200) | 1.000  (1.000-1.000) | 4.5 |
| Questionnaire translation | | | | | | | | | | | |
| *Formal translation* | 184 | 36 (19.6%) | 0.875  (0.803-0.946) | 0.913  (0.912-0.914) | 0.611  (0.452-0.770) | 0.986  (0.968-1.005) | 45.222  (11.141-183.565) | 0.394  (0.262-0.594) | 0.917  (0.806-1.027) | 0.912  (0.869-0.956) | 4.5 |
| *Ad hoc translation^£^* | 178 | 50 (28.1%) | 0.866  (0.805-0.927) | 0.798  (0.796-0.796) | 0.840  (0.738-0.942) | 0.781  (0.710-0.853) | 3.840 (2.709-5.444) | 0.205  (0.108-0.389) | 0.600  (0.485-0.715) | 0.926  (0.877-0.975) | 2.5 |
| Severity of SSI | | | | | | | | | | | |
| *No re-operation (mild SSI only)* | 360 | 73 (20.3%) | 0.855  (0.801-0.908) | 0.822  (0.821-0.823) | 0.726  (0.624-0.828) | 0.847  (0.805-0.888) | 4.736  (3.486-6.433) | 0.324  (0.222-0.472) | 0.546  (0.447-0.645) | 0.924  (0.892-0.956) | 2.5 |

SSI = Surgical Site Infection. AUROC = Area under the receiver operating characteristic curve, used as an overall measure of discrimination. PLR=Positive likelihood ratio. NLR=Negative likelihood ratio. PPV=Positive predictive value. NPV=Negative predictive value. *Overall analysis included only patients with per-protocol WHQ administration. *^£^*Includes Ad hoc, translated from English by questionnaire administrator and Ad hoc, translated from English with formal translator. ^$^events = Surgical site infection recorded using reference test of 30-day in-person FALCON trial follow-up. ^€^Cut-off scores defined using Youden’s index, in which we maximize the sum of sensitivity and specificity in the cohort of interest. Implementation of the WHQ should be supported by clinical decision making using cut-point scores in *Table 4*.

Supplementary table 9. Diagnostic accuracy of the WHQ score across different cut-points to ‘rule in’ or ‘rule out’ SSI

| **WHQ score**  **cut-off** | **True prevalence*** | **Diagnostic accuracy** | **Sensitivity** | **Specificity** | **Positive likelihood  ratio** | **Negative likelihood  ratio** | **Positive predictive  value (PPV)** | **Negative predictive  value (NPV)** |
| --- | --- | --- | --- | --- | --- | --- | --- | --- |
| **'Rule in' SSI** |  | | | | | | | |
| ≥1 | 0.621 (0.571-0.670) | 0.593 (0.542-0.642) | 0.373 (0.312-0.438) | 0.952 (0.904-0.981) | 7.842 (3.737-16.457) | 0.658 (0.593-0.730) | 0.928 (0.857-0.970) | 0.481 (0.422-0.540) |
| ≥2 | 0.441 (0.391-0.492) | 0.732 (0.685-0.775) | 0.480 (0.403-0.557) | 0.931 (0.889-0.961) | 6.937 (4.155-11.583) | 0.559 (0.482-0.649) | 0.845 (0.758-0.911) | 0.694 (0.638-0.747) |
| ≥3 | 0.307 (0.261-0.355) | 0.825 (0.783-0.861) | 0.622 (0.528-0.709) | 0.914 (0.874-0.945) | 7.273 (4.802-11.016) | 0.414 (0.327-0.522) | 0.763 (0.666-0.843) | 0.845 (0.799-0.885) |
| ≥4 | 0.242 (0.200-0.288) | 0.858 (0.820-0.891) | 0.723 (0.622-0.811) | 0.901 (0.861-0.933) | 7.334 (5.079-10.590) | 0.307 (0.221-0.426) | 0.701 (0.600-0.790) | 0.911 (0.872-0.941) |
| ≥5 | 0.201 (0.162-0.244) | 0.874 (0.836-0.905) | 0.808 (0.703-0.888) | 0.890 (0.850-0.923) | 7.364 (5.267-10.296) | 0.216 (0.137-0.341) | 0.649 (0.546-0.744) | 0.948 (0.916-0.971) |
| ≥6 | 0.173 (0.136-0.214) | 0.856 (0.817-0.889) | 0.806 (0.691-0.892) | 0.866 (0.824-0.901) | 6.017 (4.449-8.137) | 0.224 (0.137-0.366) | 0.557 (0.452-0.658) | 0.955 (0.925-0.976) |
| ≥7 | 0.149 (0.116-0.189) | 0.843 (0.803-0.878) | 0.810 (0.686-0.901) | 0.848 (0.805-0.885) | 5.348 (4.026-7.105) | 0.224 (0.131-0.381) | 0.485 (0.382-0.588) | 0.962 (0.933-0.981) |
| ≥8 | 0.137 (0.104-0.175) | 0.840 (0.800-0.875) | 0.830 (0.702-0.919) | 0.842 (0.798-0.879) | 5.247 (3.984-6.911) | 0.202 (0.111-0.366) | 0.454 (0.352-0.558) | 0.969 (0.942-0.986) |
| ≥9 | 0.103 (0.075-0.138) | 0.832 (0.792-0.868) | 0.900 (0.763-0.972) | 0.825 (0.781-0.863) | 5.134 (3.998-6.594) | 0.121 (0.048-0.308) | 0.371 (0.275-0.475) | 0.986 (0.965-0.996) |
| ≥10 | 0.085 (0.059-0.117) | 0.825 (0.783-0.861) | 0.939 (0.798-0.993) | 0.814 (0.770-0.853) | 5.053 (3.997-6.387) | 0.074 (0.019-0.286) | 0.320 (0.229-0.422) | 0.993 (0.975-0.999) |
| **'Rule out' SSI** |  | | | | | | | |
| ≤1 | 0.559 (0.508-0.609) | 0.268 (0.225-0.315) | 0.069 (0.039-0.111) | 0.520 (0.443-0.597) | 0.144 (0.086-0.241) | 1.789 (1.542-2.075) | 0.155 (0.089-0.242) | 0.306 (0.253-0.362) |
| ≤2 | 0.693 (0.645-0.739) | 0.175 (0.139-0.217) | 0.086 (0.055-0.126) | 0.378 (0.291-0.472) | 0.137 (0.091-0.208) | 2.418 (1.915-3.054) | 0.237 (0.157-0.334) | 0.155 (0.115-0.201) |
| ≤3 | 0.758 (0.712-0.800) | 0.142 (0.109-0.180) | 0.099 (0.067-0.139) | 0.277 (0.189-0.378) | 0.136 (0.094-0.197) | 3.259 (2.345-4.529) | 0.299 (0.210-0.400) | 0.089 (0.059-0.128) |
| ≤4 | 0.799 (0.756-0.838) | 0.126 (0.095-0.164) | 0.110 (0.077-0.150) | 0.192 (0.112-0.297) | 0.136 (0.097-0.190) | 4.630 (2.933-7.308) | 0.351 (0.256-0.454) | 0.052 (0.029-0.084) |
| ≤5 | 0.827 (0.786-0.864) | 0.144 (0.111-0.183) | 0.134 (0.099-0.176) | 0.194 (0.108-0.309) | 0.166 (0.123-0.225) | 4.463 (2.735-7.285) | 0.443 (0.342-0.548) | 0.045 (0.024-0.075) |
| ≤6 | 0.851 (0.811-0.884) | 0.157 (0.122-0.197) | 0.152 (0.115-0.195) | 0.190 (0.099-0.314) | 0.187 (0.141-0.248) | 4.474 (2.623-7.631) | 0.515 (0.412-0.618) | 0.038 (0.019-0.067) |
| ≤7 | 0.863 (0.825-0.896) | 0.160 (0.125-0.200) | 0.158 (0.121-0.202) | 0.170 (0.081-0.298) | 0.191 (0.145-0.251) | 4.957 (2.729-9.006) | 0.546 (0.442-0.648) | 0.031 (0.014-0.058) |
| ≤8 | 0.897 (0.862-0.925) | 0.168 (0.132-0.208) | 0.175 (0.137-0.219) | 0.100 (0.028-0.237) | 0.195 (0.152-0.250) | 8.247 (3.251-20.922) | 0.629 (0.525-0.725) | 0.014 (0.004-0.035) |
| ≤9 | 0.915 (0.883-0.941) | 0.175 (0.139-0.217) | 0.186 (0.147-0.230) | 0.061 (0.007-0.202) | 0.198 (0.157-0.250) | 13.432 (3.503-51.513) | 0.680 (0.578-0.771) | 0.007 (0.001-0.025) |
| ≤10 | 0.925 (0.894-0.949) | 0.186 (0.148-0.228) | 0.195 (0.155-0.240) | 0.069 (0.008-0.228) | 0.209 (0.166-0.264) | 11.673 (3.062-44.500) | 0.722 (0.621-0.808) | 0.007 (0.001-0.025) |

*Proportion of patients that would be classified as having an SSI event with this cut-point. All estimates provided with 95% confidence intervals in brackets.

Supplementary figure 1. Proportion of patients with successful telephone contact with increasing time from surgery (days)

**
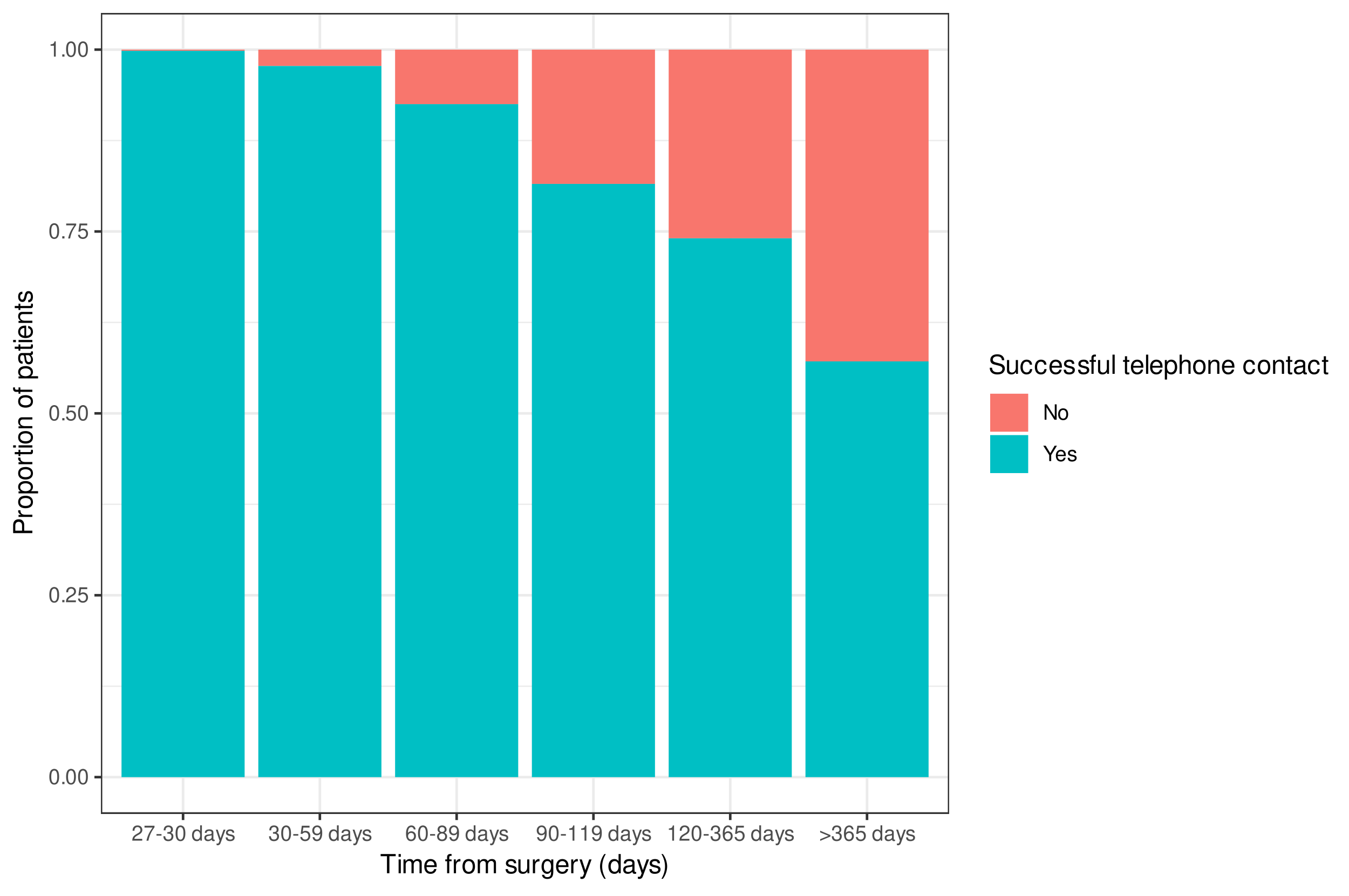
**

Supplementary figure 2. Proportion of patients with in-person FALCON trial versus telephone only follow-up (N=1088)

***
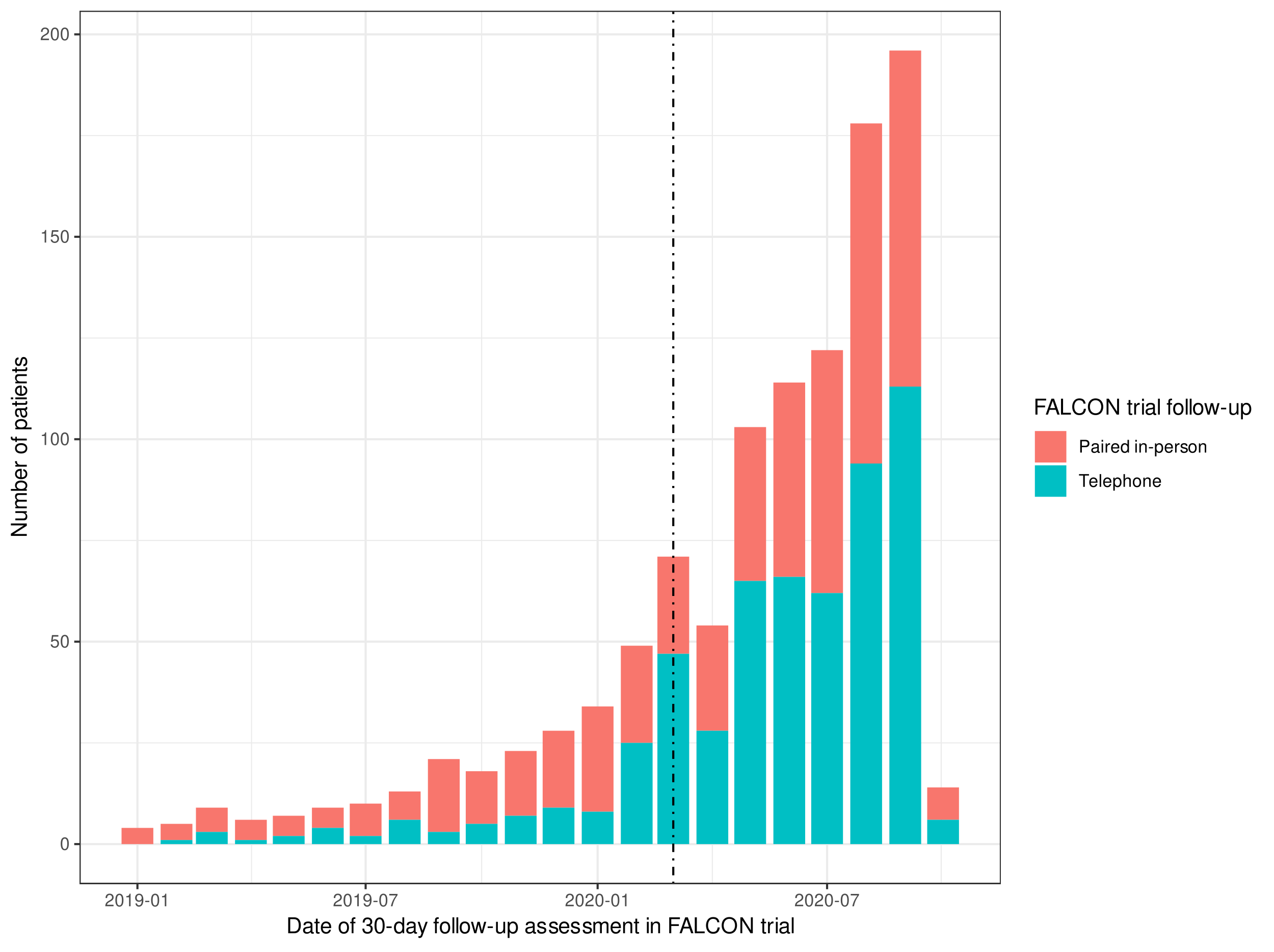
***

***
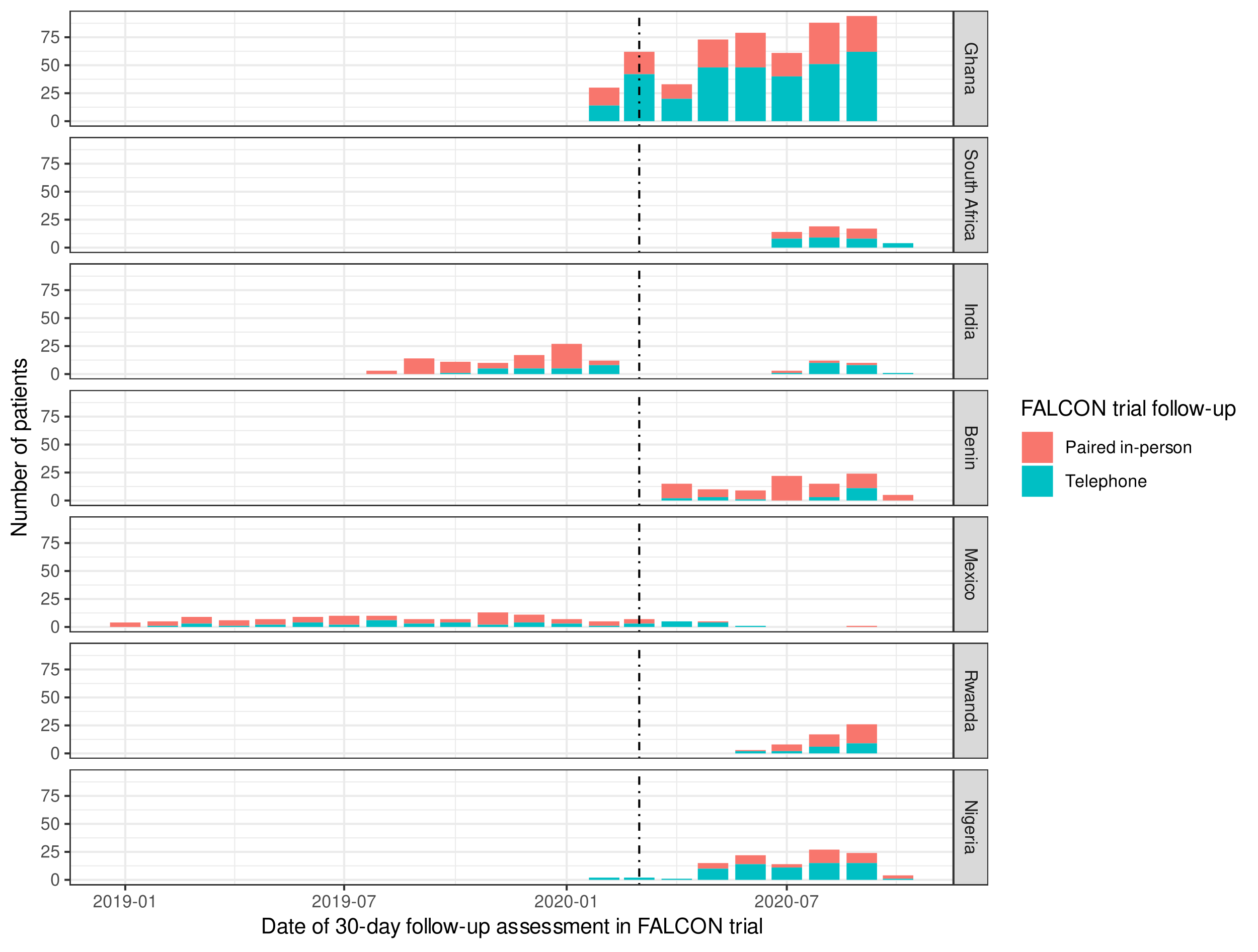
***

Grey line estimates the date of emergence of the first cases of SARS-CoV-2 outside of mainland china

Supplementary figure 3. Timing of telephone WHQ administration

**
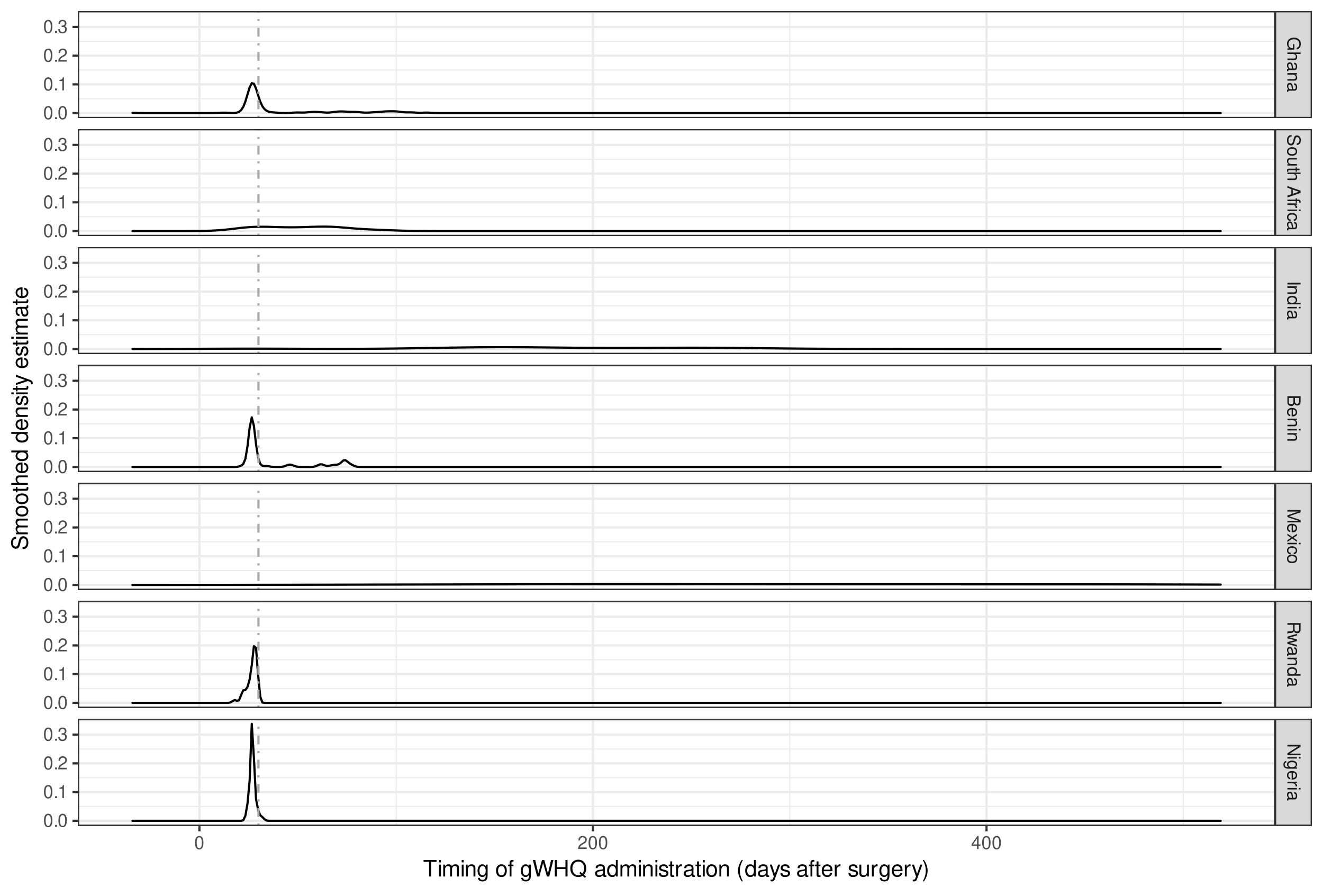
**

Grey line denotes the timing of in-person FALCON follow-up (30-days after surgery).

Supplementary figure 4 (Panel). Wound Healing Questionnaire score versus reference test diagnosis of SSI (N=388)

**
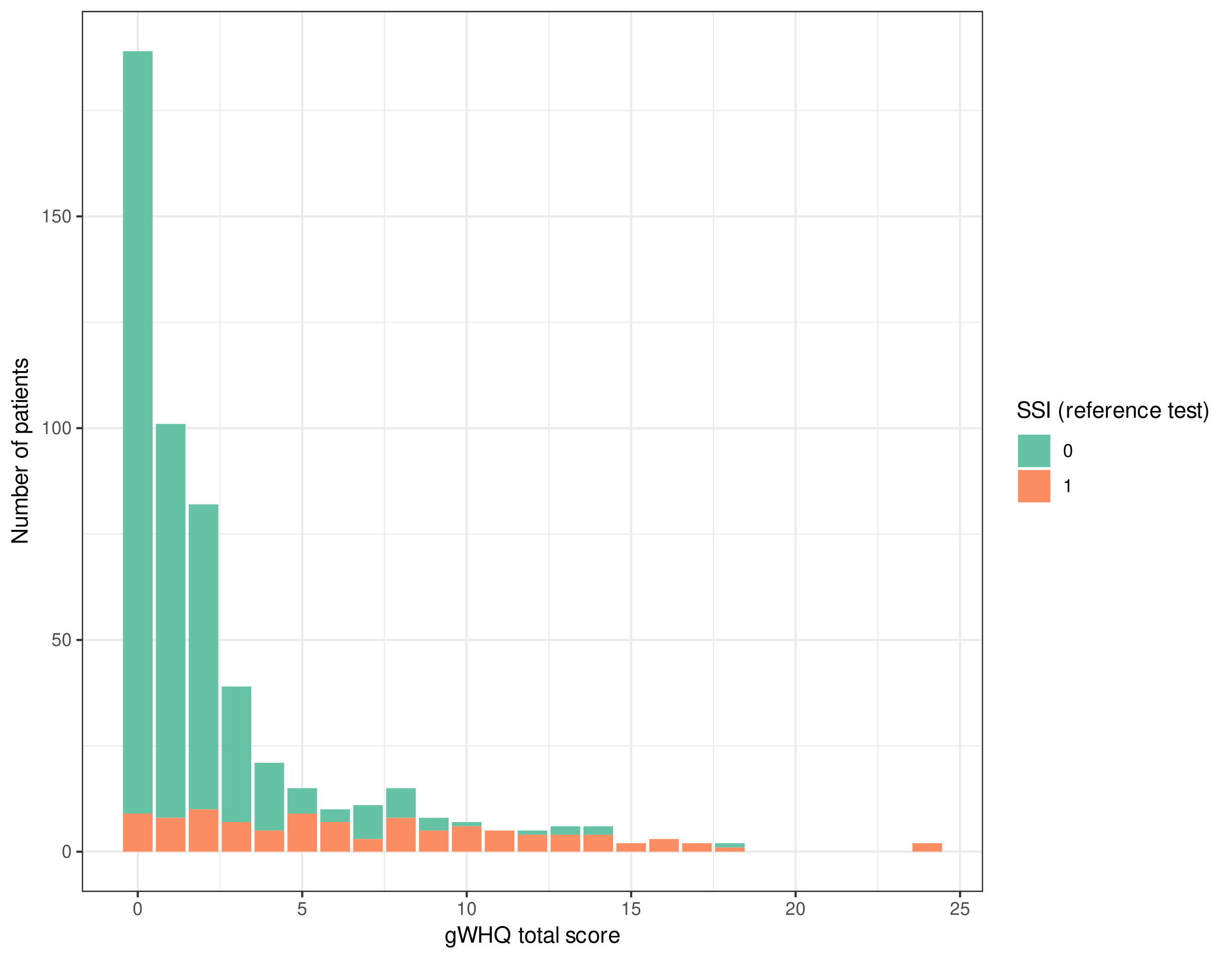
**

Graph represents per-protocol analysis (N=388)

**
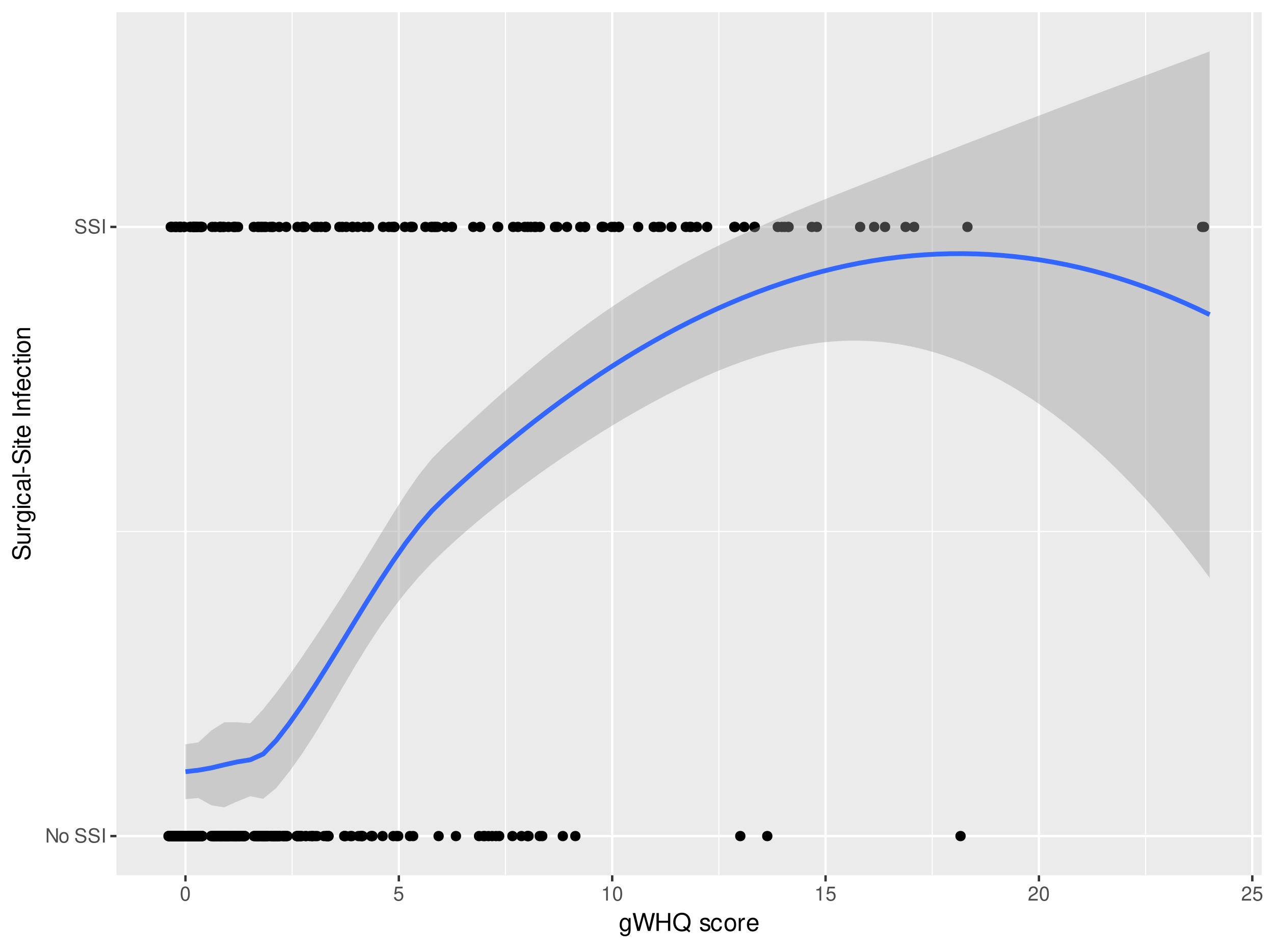

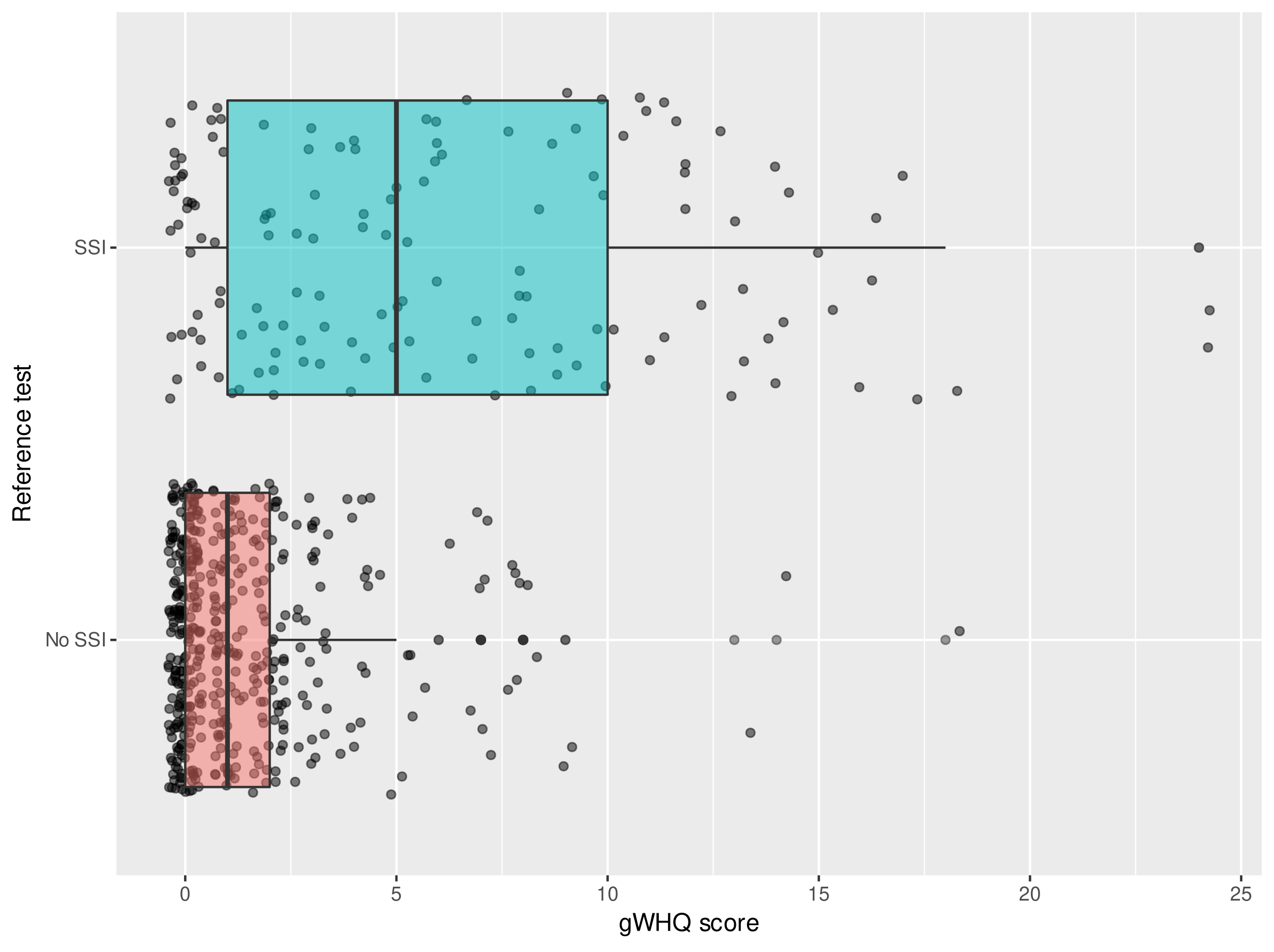
**

Supplementary figure 5. Proportion of patients with SSI diagnosis in reference test at each WHQ point score level (per-protocol analysis)

**
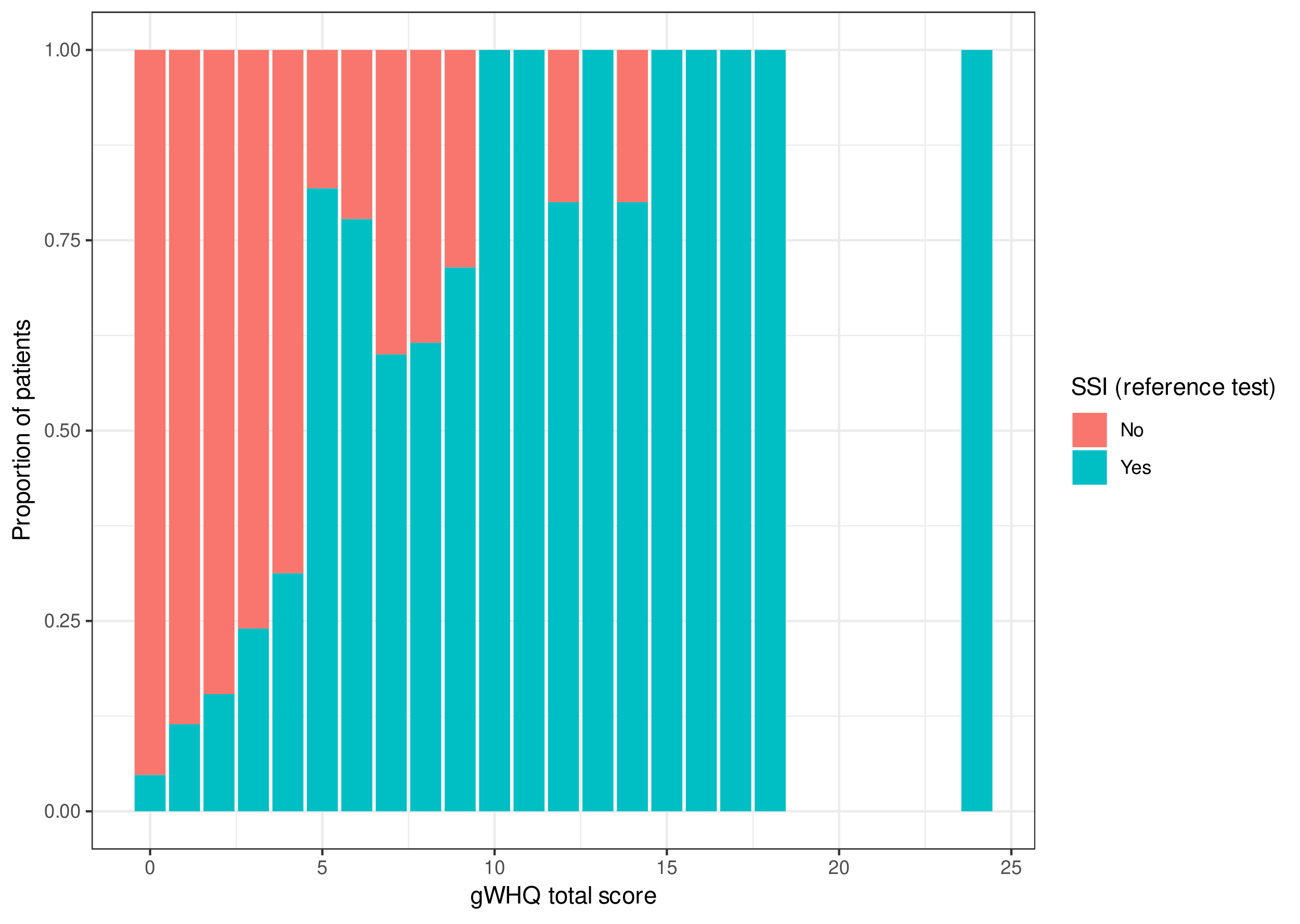
**
